# *De Novo* Glycan Annotation of Mass Spectrometry Data

**DOI:** 10.1021/jasms.5c00093

**Published:** 2025-07-03

**Authors:** Margot Bligh, Sebastian Silva-Solar, Linda Biehler, Christopher C. J. Fitzgerald, Conor J. Crawford, Mikkel Schultz-Johansen, Sofie Niggemeier, Peter H. Seeberger, Manuel Liebeke, Jan-Hendrik Hehemann

**Affiliations:** † 28267 Max Planck Institute for Marine Microbiology, 28359 Bremen, Germany; ‡ MARUM, Center for Marine Environmental Sciences, University of Bremen, 28359 Bremen, Germany; § 28321 Max Planck Institute for Colloids and Interfaces, 14476 Potsdam, Germany; ∥ Institute for Chemistry and Biochemistry, Freie Universität Berlin, 14195 Berlin, Germany; ⊥ Institute for Metabolomics, Christian-Albrecht-University Kiel, 24118 Kiel, Germany

**Keywords:** glycans, carbohydrates, mass spectrometry, R, annotation tool

## Abstract

Carbohydrates are fundamental molecules of life that are involved in virtually all biological processes. The chemical diversity of glycans—carbohydrate chains—enables diverse functions but also challenges analytics. Annotation of glycans in mass spectrometry (MS) data relies heavily on experimental databases or manual calculations, hindering the discovery of novel glycan compositions and structures. Here, we introduce GlycoAnnotateR—a package in the open-source programming language R—for *de novo* annotation of glycan compositions in MS data. GlycoAnnotateR calculates all possible monomer and modification combinations, which are then filtered against a defined set of chemical rules to provide biologically relevant compositions. The “glycoPredict” function can return compositions for oligosaccharides ranging from 1 to 22 monomers in length while accounting for four different modifications in under 10 min with less than 4 GB of random-access memory (RAM). Here, three case studies demonstrate the efficacy and versatility of GlycoAnnotateR: (1) accurate identification of mono- and oligosaccharide standards, (2) characterization of sulfated fucan oligosaccharides obtained by enzymatic digestion of fucoidan, a complex algal glycan, and (3) reproduction and expansion of glycan annotations for a published mouse lung MALDI-MS imaging data set previously annotated by NGlycDB. GlycoAnnotateR rapidly provides accurate annotations and complements existing R packages for MS data processing, enabling metabolomic and glycomic data integration. This combinatorial, rule-based approach enhances glycan annotation capabilities and supports hypothesis generation in glycoscience, expanding our ability to explore the chemical space of glycan diversity.

## Graphical abstract

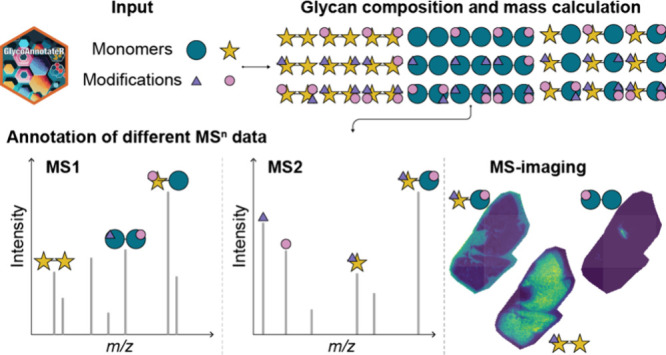

## Introduction

Carbohydrates are one of the fundamental molecules of life. Myriad biological functions—including energy storage, cell structure, signaling, communication and defense—rely on glycans,^1,2^ which are carbohydrate polymers. The carbon fixed from carbon dioxide via photosynthesis is incorporated into carbohydrates, meaning most organic carbon on Earth is funneled through a carbohydrate step.^3^ Glycosylation, the attachment of carbohydrate chains to proteins, is the most complex and common post-translational modification.^4^ The importance of glycans in human health and disease has been increasingly recognized over the last two decades, with over 100 human disorders associated with major defects in glycosylation pathways.^2^ These diverse functions are enabled through the chemical diversity of carbohydrates, which far outweighs that of DNA or proteins.^2,5^

Glycan diversity and other analytical challenges mean that high-throughput, accessible sequencing of carbohydrates remains unattainable to date. As in proteomics, mass spectrometry (MS) is the most advanced technique in glycomics, offering high sensitivity and accuracy.^6–8^ Glycans are typically ionized by electrospray ionization (ESI)^9^ or matrix assisted laser desorption ionization (MALDI).^10^ In combination with chromatography or orthogonal techniques such as ion mobility, or with certain fragmentation techniques, MS provides the ability to separate and identify isomeric carbohydrates.^6–8,11,12^

Glycan annotation is the processing of assigning glycan identities to peaks in mass spectra. Glycan composition—i.e., the number of different monomers and modifications in a glycan structure—is the most basic level of annotation, and can be achieved by comparison of observed and theoretical *m*/*z* values. For example, four desialylated N-glycans derived from bovine fibrinogen, a blood glycoprotein, were identified as constituting five hexose monomers and four N-acetylhexosamine monomers based on MS data by Fellenberg et al.; *m*/*z* values of 821.304–821.306 were observed, which deviate from the theoretical *m*/*z* value of the [M+2H]^2+^ adduct by 0.1–2.5 ppm.^13^ The four isomers were separated by porous graphitized carbon (PGC) liquid chromatography (LC). In the same study, MS/MS resolved the sequence of the monomers in each of these branched glycan structures, as well as the structures of five additional branched glycans.^13^ For example, a fragment ion with an *m*/*z* value of 366.129 was annotated as a dimer constituting one hexose and one N-acetylhexosamine (dehydrated, [M + H]^+^ ion). The intensity of this ion was diagnostic for a terminal Hex-HexNAc sequence on the glycan branches. Higher order structural information such as the stereochemistry of carbohydrate hydroxyl groups and glycosidic linkages, and the position of modifications and linkages may require inclusion of additional techniques beyond MS, such as NMR.^13^ The practical relevance of glycan annotation is manifest in the structure–function relationships of glycans; staying with the fibrinogen example, the level of sialylation of fibrinogen N-glycans impacts polymerization of blood clots, and therefore deciphering glycan structures by means of mass spectrometry has clinical implications.^14^

Automated glycan annotation currently depends primarily on experimental databases. Database matching is proposed to be sufficient for the analysis of specific glycomes that have been intensively studied,^8^ such as the serum N-glycome and the human milk glycome. The term glycome is the glycan equivalent of the proteome, and refers to the complete set of glycan structures present in a defined biological sample. However, despite the many technological advances in glycobiology over the last decades,^15^ achieving complete annotated glycan libraries for the many other glycomes of interest to biomedical, industrial, environmental and other fields remains a monumental task. As glycan structures are not encoded directly in DNA, but rather processively synthesized by a series of glycosyltransferases, glycosidases and other transferase enzymes in reactions regulated by the metabolic state of the cell and other factors, it is not currently possible to predict a glycome based only on the genome or proteome of an organism.^15^ While the nonredundant (nr) nucleotide and protein databases hosted by NCBI currently contain 97 and 595 million sequences respectively, the three main glycan structure databases GlyConnect,^16^ KEGG GLYCAN^17^ and GlyTouCan currently comprise 5,609, 11,538 and 250,837 glycan structures, respectively. The differences in the sizes of these glycan databases reflect their level of curation (high to none). These databases are patently insufficient to cover glycan diversity; for context, 45 human glycosyltransferases could theoretically synthesize more than 1.1 million N-glycan structures of 15 monomers or less.^18^ Thus, annotating glycans relying on experimental databases means that many glycans are likely to be overlooked. Identification of novel glycan compositions and structures is crucial to progress not only in glycomics but life science research in general given the prevalence of glycosylation modifications across life. For example, in 2021 it was discovered using click chemistry that small noncoding RNAs carry sialylated glycans, despite no previous link between RNA and glycans in nature.^19^ Mass spectrometry enabled identification of the glycans on RNA in this study. Easy to use and free tools for annotation of novel glycan compositions in MS data will be of importance for the growth of glycomics in medical and other life sciences.

Current options for annotation of glycans in MS data are also difficult to incorporate into pipelines with open-source tools and provide relatively limited output, hindering untargeted glycan analyses. The semiautomatic GlycoWorkBench is a widely used tool to assist interpretation of glycan mass spectra,^20^ but it is a stand-alone application, and is no longer maintained. GlycoWorkBench was part of the EuroCarb initiative that is no longer funded. Therefore, other EuroCarb web-based tools such as Glyco-peakFinder^21^ are not available. GlycoMod is a tool to annotate a short list of glycan masses,^22^ but a limited number of options (e.g., adduct types) can be queried at one time and the output has low interoperability. Promising techniques based on deep learning are developing^23^ but currently remain challenged by the sparsity of glycobiology data sets.^24^ In practice, annotation of unknown and novel glycans, especially those not part of glycoproteins^25^ such as marine algal glycans, currently relies heavily on manual calculations, which quickly becomes a prohibitively lengthy task for complex mixtures. A broadly applicable, simple, open-source tool for annotation of glycan compositions that can be incorporated into existing metabolomics workflows, for example XCMS- or CARDINAL-based pipelines in R,^26,27^ is needed.

To this end we created ‘GlycoAnnotateR’ (https://github.com/margotbligh/GlycoAnnotateR), an R package for *de novo* annotation of glycan compositions in MS data. It has three main modules: (i) calculation of possible compositions given constraining parameters (glycoPredict); (ii) annotation of putative oligosaccharide ions in MS data by comparison with theoretical *m*/*z* values of calculated compositions (glycoAnnotate); and (iii) retrieval of MS/MS peaks in XCMS objects with annotated precursor ions (glycoMS2Extract), and annotation of products ions based on the precursor annotation(s) (glycoMS2Annotate). Calculation of glycan compositions was enabled by derivation of a simple combinatorial equation to describe compositional possibilities, and construction of a set of chemical rules to filter output to meaningful possibilities. Our inclusive approach ensures signals from as many different glycans as possible are recruited, and therefore enables definition of a space nearing the potential glycome. Three use cases presented here show the utility and versatility of GlycoAnnotateR.

## Experimental Section

Vendor-specific MS data files were converted to mzML format using the ProteoWizard tool “msconvert” v3.0.20239 with a “peakPicking” filter,^28^ unless specified otherwise. Detailed information on chemical rules, oligosaccharide synthesis, and sample and data processing for the three use cases is provided in the Supporting Information.

### GlycoAnnotateR

The base code for GlycoAnnotateR was written in Python, which is called in the R package via reticulate.^29^ In the “glycoPredict” function, compositions are first “calculated” according to eq 1, then filtered by the number of modifications per monomer, a set of defined chemical rules (Table S1), and user input. The “glycoAnnotateR” and “glycoMS2Annotate” functions annotate user data by comparison of experimental and calculated *m*/*z* values, with “glycoPredict” parameters for the latter defined by precursor annotations. Detailed documentation on all parameters and three tutorials can be found on GitHub (https://margotbligh.github.io/GlycoAnnotateR/). Briefly, users can constrain the degree of polymerization (DP) range, modifications, maximum number of modifications per monomer on average, monomer types, scan range, polarity, ionization type (ESI or MALDI), adducts, labels from derivatization, and compositional rules. There is an additional option to allow for double sulfation. Hexose, pentose, deoxyhexose and sialic acid are referred to as monomers in the naming, while all other components are considered modifications. For example, N-acetylglucosamine is referred to as “Hex1 N-Acetyl1” in our approximation of IUPAC naming.^30^ If calculated compositions are in the GlycoConnect database they are linked by ID (database downloaded in tsv format in March 2024). Benchmarking of “glycoPredict” was performed with a range of parameters using Snakemake.^31^

### Use Case 1: Commercial and Synthetic Standards

Commercial mono- and oligosaccharide standards were dissolved in Milli-Q-water (Table S2). The mixture of 16 analytes was analyzed by LC-MS/MS using hydrophilic interaction liquid chromatography (HILIC) separation (Accucore 150 Amide, Thermo Fisher) and an Orbitrap mass spectrometer (Thermo Fisher Q-Exactive Plus). Standards were first analyzed in full MS mode (positive and negative polarity), and then with data-dependent MS/MS with an inclusion list (Table S3). Preprocessing (peak picking, merging, grouping and filling) was performed with XCMS v4.0.2 in R.^26^ Resulting features were grouped by retention time to enable isotope detection with CAMERA v1.56.0.^32^ Positive and negative features were annotated at 1.5 and 2 ppm, respectively. MS/MS spectra associated with annotated features were extracted, processed with MSnbase,^33^ and then fragment ions were annotated at 3 ppm.

Defined carbohydrate structures were synthesized: (1) fucan hexasaccharide; (2) glucan pentasaccharide; (3) sulfated fucan disaccharide; (4) mannan trisaccharide; (5) sulfated mannose monosaccharide. Structures **2** and **3** had an amino-pentyl linker (Figure S1). The automated glycan assembly of structures **1**, **3** and **4** followed published protocols.^34,35^ Structure **2** was prepared with dibutyl phosphate donors.^36^ Structure **5** was synthesized using solution-phase synthesis.^37^ Purification and analysis by HPLC were performed on an Agilent 1200 series equipped with an evaporative light scattering (ELS) detector (G4260B) and a single quadrupole mass spectrometer (G6135B). Mass spectra were obtained using an Agilent 6210 ESI-TOF mass spectrometer (QTOF). QTOF data were imported into R with MSnbase.^33^ Files were filtered by retention time to a 15 s window according to the total ion chromatogram peak (Figure S2). Spectra were then averaged, normalized, and filtered at a normalized intensity of 0.8 and annotated at 15 ppm. HPLC-ELS-MS files were parsed with the python package ‘rainbow’. Ion chromatograms were extracted based on the annotations assigned to the corresponding QTOF data (±0.1 Da).

### Use Case 2: Fucoidan Enzyme Digest

 fucoidan (Carbosynth; 3 mg mL^–1^) was digested overnight with the recombinantly expressed GH107 *endo-*fucoidanase Wv323.^34^ The negative control contained heat-inactivated enzyme. Digests were desalted using silica spin columns,^38^ with loading at 95% (v/v) ethanol and elution of oligosaccharides at 85% (v/v) ethanol. Crude digests and flow-through fractions were visualized by carbohydrate polyacrylamide gel electrophoresis (C-PAGE) with Stains-All dye.^39,40^ Digests were analyzed in negative polarity by LC-MS with the same instrumentation as commercial standards. LC-MS data were processed with XCMS^26^ and GlycoAnnotateR to generate a timed inclusion list (Table S4) for LC-MS/MS with targeted selected ion monitoring and data-dependent MS/MS acquisition. MS/MS spectra were extracted and annotated with GlycoAnnotateR. Data files are available on MassIVE (doi:10.25345/C57D2QK2 V).

### Use Case 3: Publicly Available MALDI-MSI Data Set

NGlycDB v1 annotations assigned at a false discovery rate (FDR) of 5% to a mouse lung MALDI-FTICR data set (“20210401_lung_p27_1”) were downloaded in csv format from METASPACE.^41^ The *m*/*z* values in the downloaded table were reannotated by GlycoAnnnotateR, using a custom database built with glycoPredict for mammalian N-glycans. The imzML file was also downloaded from METASPACE, and preprocessed (peak picking, aligning and binning) with Cardinal v3.2.1 in R.^27^ The same custom database was used for annotation of preprocessed data. Annotated features were then segmented using spatial shrunken centroids.

## Results and Discussion

### Theoretical Compositions Calculated by GlycoAnnotateR

GlycoAnnotateR calculates all possible oligosaccharide compositions, given a set of constraining parameters, to annotate ions (Figure 1A). Composition is defined here as the number and type of monomers and modifications in an oligosaccharide. For flexibility, we consider pentoses and hexoses as the fundamental monomer blocks. All functional groups therefore become ‘modifications’ (e.g., N-acetyl, amine, carboxylic acid groups). Certain modified monomers e.g., uronic acids are typically considered monomers in their own right. However, our deconvolution allows maximum flexibility in building compositions, as modifications are not assigned to any specific monomers. The maximum number of different compositions can be obtained following eq 1

1 $$N=\overset{D}{\underset{i=d}{\sum }}{\left(i+1\right)}^{m+c-1}$$

where *N* is the number of different compositions, *d* is the lowest degree of polymerization (DP), *D* is the highest DP, *m* is the number of different modifications, and *c* is the number of different monomer types (defined by number of C in ring). Possible modifications include carboxylic acid, sialic acid, deoxy, phosphate, sulfate, N-acetyl, O-acetyl, O-methyl, anhydro-bridge and amino. GlycoAnnotateR also supports inclusion of labels or linkers added to glycans e.g., an amino-pentyl linker,^36^ and custom labels or modifications. If two types of monomers are considered, i.e., hexose and pentose (*m* = 0, *c* = 2), *N* corresponds to the summation of the number of multisubsets of size *i* from a set of size 2, as represented with a Pascal’s triangle (Figure 1B). *N* grows exponentially with the number of different modifications. With a DP of two, one type of monomer and modification (e.g., O-methyl; *i* = 2, *c* = 1, *m* = 1), three compositions are possible as (2 + 1)^1^ = 3: Hex2, Hex2 O-Methyl1, Hex2 O-Methyl2. There are nine possible compositions with two modifications (e.g., O-methyl and amino: *i* = 2, *c* = 1, *m* = 2) as (2 + 1)^2^ = 9 (Figure 1A, step 3). If pentose and hexose monomers are considered (*i* = 2, *c* = 2, *m* = 2), *N* increases to 27 ((2 + 1)^3^ = 27) (Table S5). The high compositional diversity of oligosaccharides (Figure 1D) demands the abstracted calculation approach taken here in order to include novel compositions.

**Figure 1:**
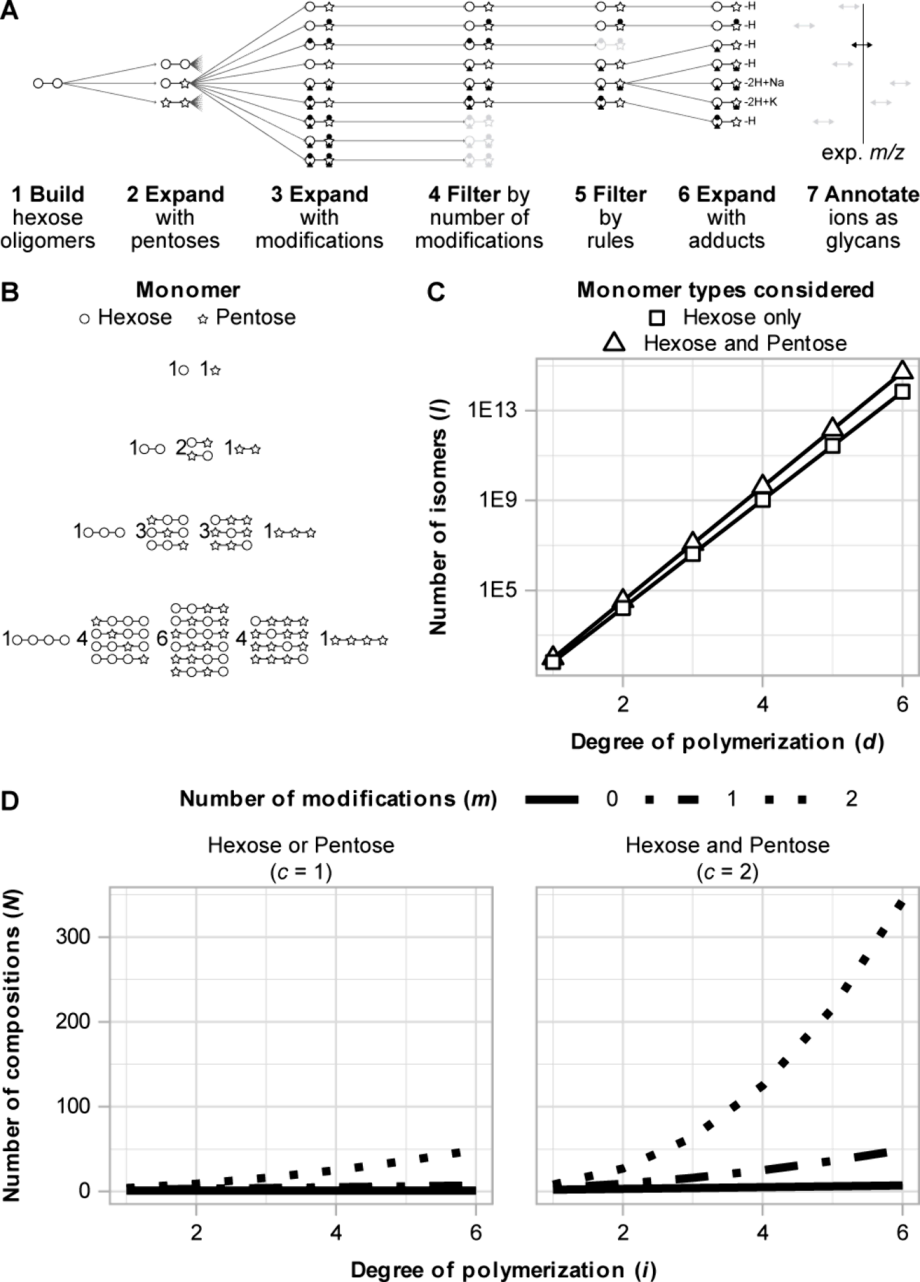
Calculations for the total number of possible compositions for modified hexose and/or pentose-based oligosaccharides. (A) Example of the basic workflow of GlycoAnnotateR. (1) Hexose monomers of the chosen degree of polymerization (DP) are “built” (here DP = 2). (2–3) Expansion to account for pentose monomers and modifications (here there are 2 possible modifications). (4) Compositions are filtered by the average number of modifications per monomer; here, the number must be ≤ 1. Grayed out compositions are removed. (5) Compositions are filtered by a set of defined chemical rules. Here, the “dot modification” cannot be on a hexose. (6) Expansion for adducts. Here, H, Na and K adducts are considered for negative MALDI ionization. The ‘triangle modification’ represents an anionic group, therefore [M-2H+Na]^−^ and [M-2H+K]^−^ are computed for compositions with a triangle modification. (7) Annotation of experimental *m*/*z* value(s) by comparison with calculated *m*/*z* values. (B) When *c* is 2 (i.e., hexose and pentose monomers are possible), the number of nonmodified compositions (*N*) equals DP plus one. Row number is equal to DP, the number of columns in each row is *N* for that row, and each number represents the total number of ordered combinations. (C) The numbers of possible isomers for linear oligosaccharides composed of only hexoses, or hexoses and pentoses, of DP1–6 (eq 2). (D) The numbers of possible compositions for oligosaccharides of DP 1–6 summarized with *c* values of 1 (left, hexose or pentose) and 2 (right, hexose and pentose) and 0–2 modifications (*m*) (eq 1).

Structural diversity is considered in eq 2

2 $$I=2^{d}\times 2^{H+3d}\times \frac{d!}{H!\left(d-H\right)!}\times \left(4^{H}\times 3^{d-H}\times \left(\frac{d-H}{3d}+\frac{H}{4d}\right)\right)\times 2^{d}$$

where *I* is the number of isomers, *d* is the DP, and *H* is the number of hexose monomers. eq 2 is a generalization of Laine’s 1994 calculations for linear structures of hexoses that account for linkage position and stereochemistry, and order, stereochemistry and ring form of monomers.^5^ Equation 2 additionally considers pentose monomers. The number of linear structures possible for a hexasaccharide increases to 518,781,583,491,072 (519 trillion) (Figure 1C). We could not account for modifications in eq 2 due to specific constraints of each functional group, which challenged summarization in combinatorial form. Equation 2 is explained in detail in the Supporting Information.

Compositions calculated in eq 1 are filtered to be meaningful. Equation 1 assumes (i) each modification can occur once per monomer, and (ii) each monomer can have all modifications at once. These assumptions are however not always true. Monomers typically carry at most 3 modifications, for example, disulfated fucose in fucoidans,^42^ but generally this number is much lower. Computed compositions are filtered by the average number of modifications per monomer, with a maximum allowed value of 3. Further filtering is applied based on a set of rules for which modifications can occur on the same monomer, and on which monomers (Table S1), which was compiled from investigation of published structures and chemical rules (see Supporting Information for details). Users can also manually limit specific elements or include the limits previously described for N- and O-linked glycans.^22^

The function “glycoPredict”, which calculates compositions as described, finishes in less than 10 min using less than 4 Gb of random-access memory (RAM) for a DP range of 1 to 22, accounting for four different modifications, and pentose and hexose monomers (Figure S3). Therefore, it will easily run on a standard computer.

### GlycoAnnotateR Accurately Annotates Simple Glycans

The accuracy of GlycoAnnotateR was validated with LC-MS/MS analysis of 16 commercial carbohydrate standards ranging from DP 1–6 and featuring alditol, deoxy, carboxylic acid, O-methyl, amino, N-acetyl, sulfate, and anhydro-bridge modifications. All 15 detectable standards were correctly annotated in positive and/or negative polarities after XCMS^26^ and CAMERA^32^ preprocessing in R (Table S6, Figure 2A, Figure S4). No galacturonic acid peak was detected due to streaking and poor ionizability (Figure S5). Annotation of multiple adducts in different polarities and isotope detection helped to resolve ambiguous annotations in MS1 data (Table S6, Figure 2A, Figure S6). Negative polarity MS/MS spectra associated with annotations were extracted and annotated with GlycoAnnotateR to confirm MS1 annotations (e.g., Figure 2C). Fourteen ions deriving from impurities and in-source fragmentation, distinguishable by retention time, were also annotated (Table S6, Figure S4). The latter were frequently annotated as “AnhydroBridge”-containing compounds, likely resulting from dehydration reactions during ionization.^43^ “AnhydroBridge”, “Unsaturated”, and “Dehydrated” modifications all result from loss of a water, but refer to different structural features (see Supporting Information). “AnhydroBridge” was selected as a possible modification in this analysis for the carrageenan standards. The detection of 14 ions likely corresponding to impurities and in-source fragmentation products even in this simple analysis highlights the need for automated annotation. Annotation of impurities and in-source fragmentation products is important to control for contaminants and reduce the size of the “dark metabolome”.^44^ Thus, GlycoAnnotateR, in combination with existing tools, allows users to harness the full sensitivity and complexity of LC-MS data.

**Figure 2:**
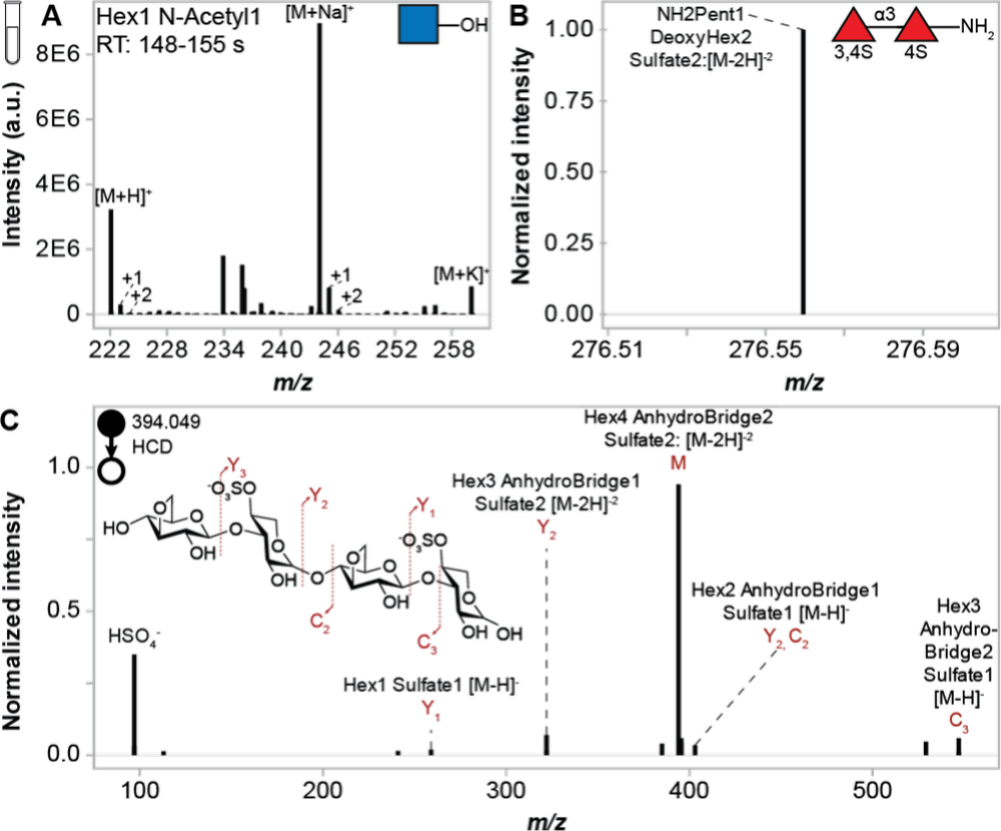
Commercial and synthetic mono- and oligosaccharide standards accurately annotated by GlycoAnnotateR. Known structures are shown in each panel. Annotations (black text) show the output format of GlycoAnnotateR. (A) Average HILIC-ESI-Orbitrap mass spectrum (retention time 148–155 s) of N-acetylglucosamine with annotations of adducts and isotopes. (B) Averaged and normalized mass spectrum of a synthetic trisulfated fucose dimer directly infused into a QTOF mass spectrometer. Only peaks with normalized intensities >0.8 are shown. One sulfate group was lost during ionization. (C) Fragment ions generated by HILIC-ESI-Orbitrap MS/MS of k-carrageenan DP 4 (Hex4 Anhydrobridge2 Sulfate2 [M-2H]^−2^, *m*/*z* 394.049) and annotated by GlycoAnnotateR. Exact fragment identities are shown in red text.

Since commercial oligosaccharides are typically derived from natural sources, impurities can generally only be avoided by chemical synthesis. To unambiguously demonstrate the accuracy of GlycoAnnotateR, annotations were performed on QTOF data from five synthetic mono- and oligosaccharides (Figure S1). All compounds were assigned a single, correct annotation by GlycoAnnoteR in one 15 ppm annotation step (Figure 2B, Figure S7).

### GlycoAnnotateR Maps Highly Sulfated Oligosaccharides from Fucoidan

To demonstrate its utility in LC-MS data exploration, GlycoAnnotateR was used to identify unknown oligosaccharides obtained by enzymatic hydrolysis of fucoidan. Fucoidans are sulfated fucose-rich polysaccharides from brown algae^46^ of growing interest due to their cosmetic,^46^ therapeutic^47^ and carbon sequestration potential.^48,49^ Combining characterization of carbohydrate-active enzymes with MS analysis of the oligosaccharides generated by these enzymes is a promising approach for structural characterization of glycans, especially complex glycans such as fucoidan.^50^ Different brown algae species synthesize fucoidans with different compositions and structures.^45,51^ Here, we examined fucoidan from *M. pyrifera*, a globally important kelp.^52^ The GH107 *endo*-fucoidanases P5AFcnA and Wv323 were recently shown to have similar but distinct activities on α-1,3 fucosyl linkages.^34,50^ Since GH107_P5AFcnA is active on *M. pyrifera* fucoidan,^50,53^ we hypothesized that GH107_Wv323 would also be active. We confirmed this new enzyme–substrate pair by C-PAGE (Figure 3A), and then proceeded to analyze the liberated oligosaccharides with HILIC separation and an FT-Orbitrap mass spectrometer.

**Figure 3:**
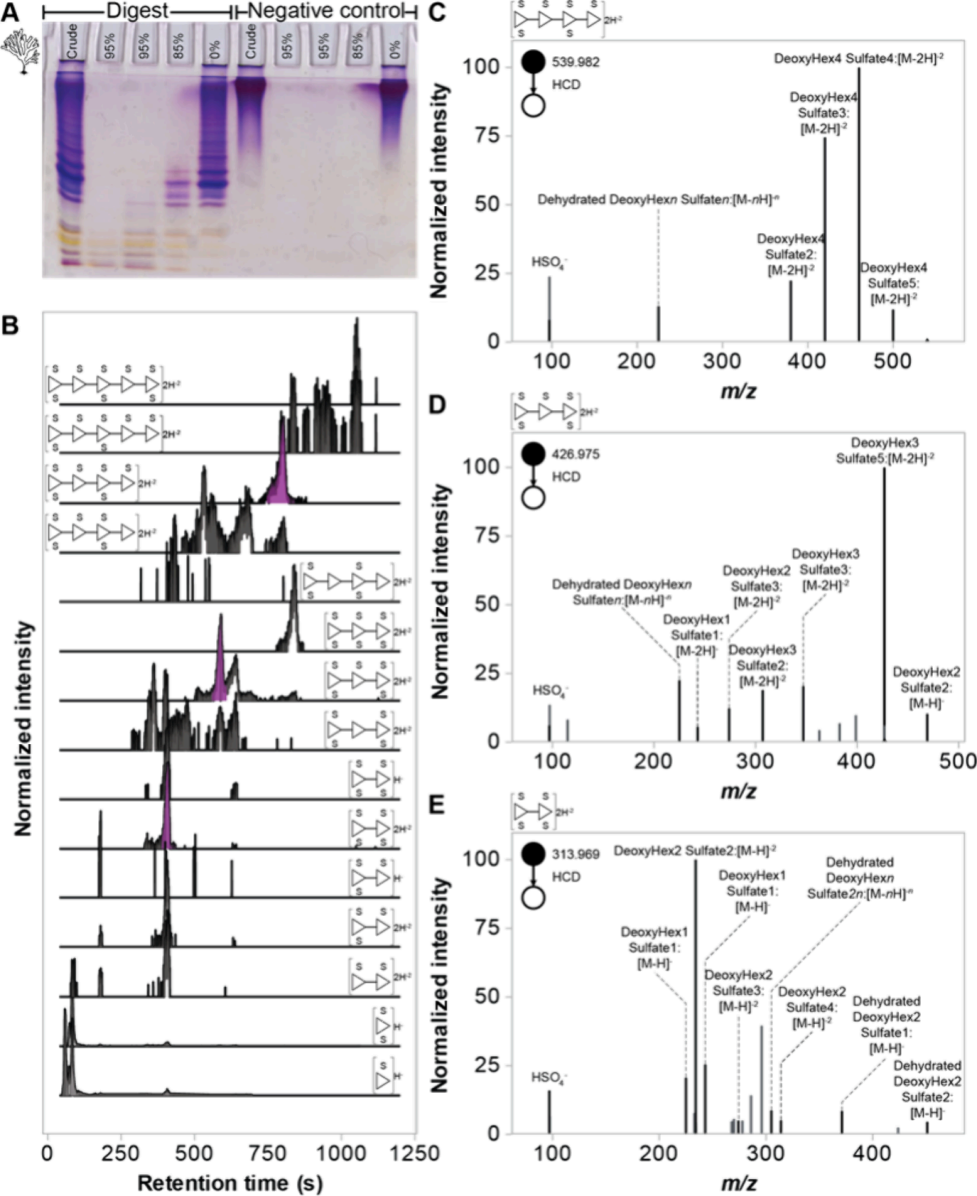
Enzymatic digestion of fucoidan with GH107_Wv323 yielded a series of sulfated oligosaccharides. (A) Sulfated oligosaccharide reaction products separated by C-PAGE according to size and charge and visualized with Stains-All dye.^35,36^ Digest: fucoidan + GH107_Wv323 enzyme. Negative control: fucoidan + inactive GH107_Wv323 enzyme. From left to right the wells were loaded with the crude digest (‘initial’) and filtrates eluted from desalting columns (percentage indicates % ethanol (v/v) with Milli-Q-water). The ladder-like pattern of bands is indicative of *endo*-acting enzyme activity. (B) Extracted ion chromatograms for oligosaccharide ions that were annotated in LC-MS data by GlycoAnnotateR. SNFG symbols indicate hypothetical structures based on the annotated compositions. Note that complete structures were not resolved in this analysis. Mean *m*/*z* values from bottom to top: 243.018, 322.975, 234.013, 273.992, 548.991, 313.969, 628.948, 386.999, 426.977, 466.956, 460.028, 500.007, 539.986, 652.994, 692.972. Purple shaded areas indicate precursor peaks for C-E. (C–E) Normalized fragmentation spectra extracted and annotated by GlycoAnnotateR from selected ion monitoring of the digest. The inclusion list was generated using the information from B. Precursor *m*/*z* values and annotations (hypothetical SNFG) are shown in the top left and above each panel, respectively. Annotation formatting represents the output of GlycoAnnotateR. Annotations with *n* values indicate peaks assigned multiple compositions. Values of *n:* (C) 1, 2, 3, 4; (D) 1, 2, 3; (E) 1, 2. Spectra were averaged across retention times: (C) 753–815 s; (D) 580–650 s; (E) 400–414 s.

The digest products, first putatively annotated in LC-MS data and then confirmed by LC-MS/MS, were identified as a series of highly sulfated oligosaccharides ranging from DP 1 to 5. All 13 unique compositions assigned to products contained only deoxyhexoses most likely representing fucose, which constitutes 50–90% of fucoidan monomers^45,54,55^ (Figure 3B). This analysis resolved only compositions, not structures, and therefore deoxyhexoses cannot be assigned identities. Two hexose-containing annotations were also assigned to peaks, but these peaks were also present in the negative control (Figure S9). After accounting for isomers, adducts and in-source fragmentation, we identified 18 distinct sulfated mono- and oligosaccharide structures in the digest that were absent from the negative control (Figure 3B, Figure S9, Table S7). We found an average deoxyhexose: sulfate ratio of 1:1.49, 3-fold lower than the reported fucose: sulfate ratio of 1:0.58.^54,55^ This disparity could indicate differences between fucoidan extracts, or heterogeneous sulfation along the polysaccharide, both of which are known features of fucoidans.^45,56^ On-the-fly analysis of LC-MS data with XCMS^26^ and GlycoAnnotateR generated an inclusion list for a targeted selected ion monitoring data-dependent MS/MS experiment (Table S4). MS/MS spectra were extracted and annotated for eight peaks with GlycoAnnotateR, which confirmed the MS1 annotations (Figure 3 **C-E**, Table S8–15). Annotations showed fragmentation of labile sulfate, hydroxyl groups (resulting in dehydrations) and glycosidic bonds, consistent with chain shortening and charge losses from the precursor ions. This experiment exemplifies the practical usefulness of GlycoAnnotateR in first assigning putative annotations to unknown, novel oligosaccharides, and then guiding and analyzing MS/MS experiments to confirm those annotations.

### GlycoAnnotateR Reproduced and Expanded MALDI-MSI Data Set Annotations

To demonstrate compatibility with MALDI-MS imaging and compare to an established tool, we reanalyzed a mouse lung imaging MALDI-FTICR data set available on METASPACE. This data set originates from the publication of NGlycDB, an N-glycan database derived from GlyConnect that is integrated into METASPACE.^41^ GlycoAnnotateR is not presented as a direct alternative to NGlycDB, but rather as a complementary tool for R-based workflows and hypothesis generation.

In a first comparison of only the annotation step, GlycoAnnotateR reproduced all NGlycDB annotations, and assigned additional annotations. The NGlycDB annotation table using the spatially informed false discovery rate (FDR) of 5% exported from METASPACE contained 88 peaks,^41^ which we annotated with GlycoAnnotateR. Chosen parameters were suitable—according to rules defined previously^22^ —for mammalian N-glycans, which do not typically contain pentose monomers.^57^ Except for four peaks that were assigned pentose-containing annotations by NGlycDB, and are therefore likely false annotations, all peaks were assigned the same annotation by NGlycDB and GlycoAnnotateR (Table S16). In addition, 25% of the peaks were assigned more than one annotation by GlycoAnnotateR, with 1.45 ± 1.03 (SD) annotations per peak on average. In some cases, the new annotations had lower mass deviations from the *m*/*z* values of the peaks than the NGlycDB annotations (Table S16). GlycoAnnotateR is therefore not only comparable to the established NGlycDB tool, but also generates novel, more diverse annotations potentially important for further investigation.

In an additional application case for GlycoAnnotateR the centroided MALDI-FTICR data were preprocessed in R with Cardinal.^27^ Here, GlycoAnnotateR assigned glycan annotations to approximately 15-fold more peaks than NGlycDB. 1,355 peaks were annotated by GlycoAnnotateR. 64 of the 88 NGlycDB annotations (5% FDR) were reproduced in the analysis, with the difference attributable to preprocessing differences between METASPACE and Cardinal. Annotated peaks were segmented into five classes based on their spatial distributions, with 78.8 peaks per class on average. Two distributions identified in the original analysis,^41^ corresponding to peaks annotated as N-glycans distributed throughout the lung tissue and at the main aerial vessel, were represented by two classes (Figure 4A–D, Figure S10). A third class contained peaks abundant at the outer edges of the tissue section, potentially the mesothelium, where sulfated glycans have been found^58^ (Figure 4F–I). The top-ranked peaks in this class were annotated as trisulfated glycans (Table S17, Figure S11), none of which are currently in GlyConnect. Many of the annotated ions in this class appear to represent fragments or adducts of each other. For example, three ions that colocalized to the outer edge have masses matching those of fragments of a putative, trisulfated hybrid N-glycan (Figure 4E–I; Table S18). Two ions in this group are annotated as adducts of the same N-glycan fragment (Figure 4G,H). While these annotated compositions require confirmation, for example by MS/MS, they suggest a potential biologically meaningful pattern of glycans with novel compositions.

**Figure 4:**
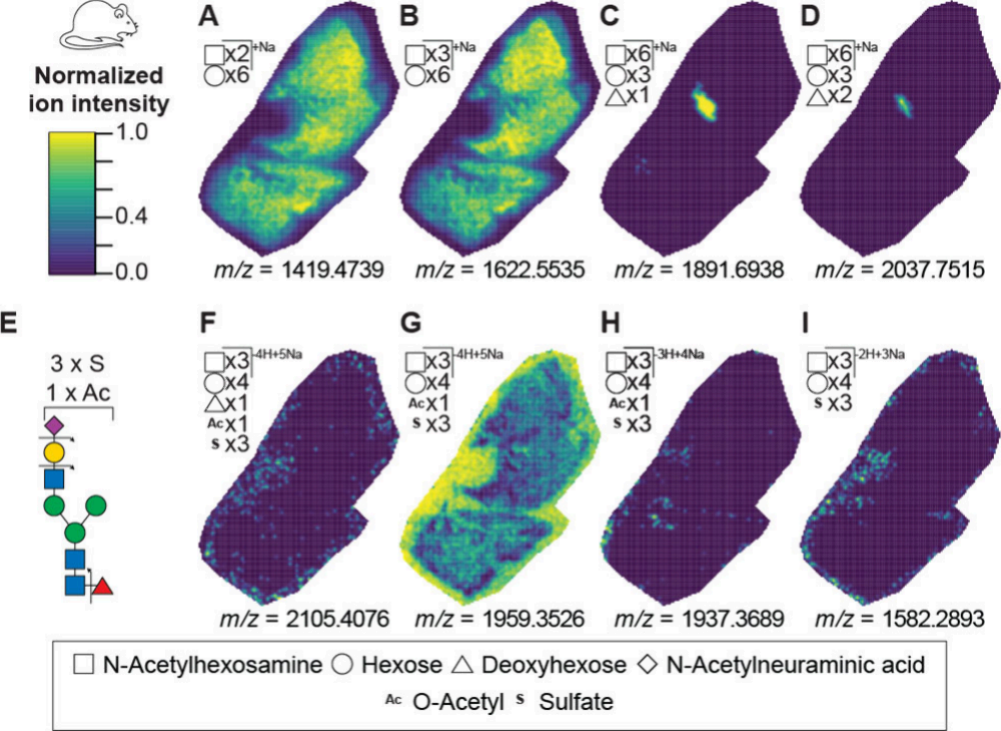
GlycoAnnotateR reveals a pattern of ions annotated as trisulfated glycans localized to the edges of a mouse lung tissue section. The mouse lung imaging MALDI-FTICR data (METASPACE: “20210401_lung_p27_1”)^37^ was preprocessed with Cardinal^23^ and annotated by GlycoAnnotateR. Example ion images are shown for peaks in different classes after segmentation analysis of annotated peaks. The *m*/*z* values are indicated below each panel (±0.01 Da). Colors indicate normalized ion intensities. Annotated compositions are represented with SNFG symbols. (A,B) Example images for a class distributed throughout the tissue: Hex8 N-Acetyl2: [M + Na]^+^ and Hex9 N-Acetyl3: [M + Na]^+^ respectively. Annotated compositions are represented with SNFG symbols. (C,D) Example images for a class colocalized with the main aerial vessel: Hex9 DeoxyHex1 N-Acetyl6: [M + Na]^+^ and Hex9 DeoxyHex2 N-Acetyl6: [M + Na]^+^ respectively. (E) Hypothetical structure of a trisulfated, hybrid N-glycan. The ion image for the theoretical *m*/*z* value of the [M-4H+5Na]^+^ ion of this structure is shown in (F). Bond cleavages shown by arrows could have generated the ions shown in (G–I), which are annotated as Hex7 N-Acetyl3 NeuAc1 O-Acetyl1 Sulfate3 [M-4H+5Na]^+^ and [M-3H+4Na]^+^, and Hex7 N-Acetyl3 Sulfate3 [M-2H+3Na]^+^ respectively.

## Conclusion

Here, we demonstrate that GlycoAnnotateR facilitates the *de novo* annotation of glycan compositions in MS data. We show the flexibility of the tool across multiple MS platforms, including with ESI and MALDI ionization, and QTOF, quadrupole, Orbitrap and FTICR mass spectrometers. GlycoAnnotateR works across different R-based mass spectrometry pipelines with different packages, allowing for integration with metabolomics data sets. Such integration will be required for systems-level glycobiology. GlycoAnnotateR allows for a diversity of monomers, modifications, and labels added by derivatization, aiding the analysis of glycans from diverse systems and prepared in diverse workflows. Utility was demonstrated here for commercial and synthetic standards, a sulfated algal glycan, and mammalian glycans. The inclusion of diagnostic fragment ions in future iterations of the tool will further increase its utility. The calculation component driving the tool means that novel glycan structures can be identified for further analysis, for example in biomarker discovery. Once validated, these structures can then populate the currently sparse glycan databases, facilitating future structure–function studies to understand the biological roles of glycans.

## Supplementary Material



## Data Availability

LC-MS data for commercial standards are available on Github (https://github.com/margotbligh/GlycoAnnotateR/tree/master/inst/extdata). LC-MS data for the fucoidan digest are available on MASSive.

## Notes

Detailed information on chemical rules, oligosaccharide synthesis, sample preparation, data acquisition, and data processing including additional tables and plots (PDF)
