# Supplementary material for: De Novo Glycan Annotation of Mass Spectrometry Data
Source: J Am Soc Mass Spectrom. 2025 Jul 3;36(8):1686–95. doi: 10.1021/jasms.5c00093 (PMC12333352; doi:10.1021/jasms.5c00093)
Supplement: Supplementary file 1 [file js5c00093_si_001.pdf]

Supporting Information

De novo glycan annotation in mass spectrometry data

Margot Bligh[1,2*], Sebastian Silva-Solar[1], Linda Biehler[1,2], Christopher C. J. Fitzgerald[1,2], Conor J. Crawford[3], Mikkel Schultz-Johansen[2], Sofie Niggemeier[1,2], Peter H. Seeberger[3,4], Manuel Liebeke[1,5], Jan-Hendrik Hehemann[1,2]

[1]Max Planck Institute for Marine Microbiology, 28359 Bremen, Germany; [2] MARUM, Center for Marine Environmental Sciences, University of Bremen, 28359 Bremen, Germany; [3]Max Planck Institute for Colloids and Interfaces, 14476 Potsdam, Germany; [4]Institute for Chemistry and Biochemistry, Freie Universität Berlin, 14195 Berlin, Germany; [5]Institute for Metabolomics, Christian-Albrecht-University Kiel, 24118 Kiel, Germany

*mbligh@mpi-bremen.de

Table of Contents

Supplementary Tables 4
Table S1 | Rules on combinations of modifications on a monomer in GlycoAnnotateR. 4
Table S2 | Commercial mono- and oligosaccharide standards used in the standard mixture. 5
Table S3 | Commercial standards DDA inclusion list 5
Table S4 | Timed inclusion list for targeted selected ion monitoring (tSIM) with ddMS2 of *M. pyrifera* fucoidan digest. 6
Table S5 | The 27 compositions that are possible with pentose and hexose monomers, amino and O-methyl modifications, and a DP of 2. 7
Table S6 | Commercial mono- and oligosaccharides standards annotated in LC-MS data. 8
Table S7 | 18 distinct mono- and oligosaccharides annotated in LC-MS data of *M. pyrifera* digest. 9
Table S8 | Annotated fragment ions from MS/MS of DeoxyHex1 Sulfate1 [M-H]- from 50-70 s. 10
Table S9 | Annotated fragment ions from MS/MS of DeoxyHex2 Sulfate [M-2H]-2 from 400-414 s. 11
Table S10 | Annotated fragment ions from MS/MS of DeoxyHex3 Sulfate5 [M-2H]-2 from 580-613 s. 12
Table S11 | Annotated fragment ions from MS/MS of DeoxyHex3 Sulfate5 [M-2H]-2 from 614-650 s. 13
Table S12 | Annotated fragment ions from MS/MS of DeoxyHex3 Sulfate6 [M-2H]-2 from 820-852 s. 13
Table S13 | Annotated fragment ions from MS/MS of DeoxyHex4 Sulfate5 [M-2H]-2 from 661-686 s. 14
Table S14 | Annotated fragment ions from MS/MS of DeoxyHex4 Sulfate6 [M-2H]-2 from 754-816 s. 14
Table S15 | Annotated fragment ions from MS/MS of DeoxyHex5 Sulfate8 $[M-2H]^{-2}$ from 1044-1045 s. 15
Table S16 | GlycoAnnotateR annotation of NGlycDB annotations for mouse lung MALDI-MSI data downloaded from METASPACE. 16
Table S17 | Top-ranked peaks in segmentation class containing peaks localised to the outside edge of the mouse lung section. 20
Table S18 | Peaks co-localised to outer edge of mouse lung section. 21

Supplementary Figures 22
Figure S1 | Synthetic compounds used in this study. 22
Figure S2 | TICs of QTOF data from synthetic oligosaccharides. 22
Figure S3 | Benchmarking of ‘glycoPredict’ function. 23
Figure S4 | Extracted ion chromatograms from LC-MS analysis of a mixture of commercial standards. 24
Figure S5 | No galacturonic acid $[M-H]^-$ peaks were picked in LC-MS data of commercial standards. 25
Figure S6 | Isotopes of negative mode 403.0546 *m/z* feature at ~1.7 min helped to resolve ambiguous annotation. 25
Figure S7 | Synthetic standards annotated in QTOF data. 26
Figure S8 | HPLC-ELS-MS traces for synthetic compounds. 27
Figure S9 | Extracted ion chromatograms from fucoidan digest negative control. 28
Figure S10 | Spatial distribution patterns of annotated glycans previously obtained with METASPACE and NGlycDB were reproduced with Cardinal and GlycoAnnotateR. 29
Figure S11 | Spatial distribution pattern of ions annotated as tri-sulfated glycans by GlycoAnnotateR. 30
Figure S12 | Solid-phase resin and thioglycoside building blocks used in this study. 31
Figure S13 | Crude HPLC of automated synthesis of glucan. ELSD Trace. 31

Supplementary Information 33

1. **Extended methods** 33
   1.1) Chemical rules 33
   1.2) Benchmarking 40
   1.3) LC-MS/MS of commercial standards 41
   1.4) Oligosaccharide synthesis and analysis 43

Table S19 | TMSOTf acidic wash (Module a) summary. 45
Table S20 | Thioglycoside glycosylation (Module b) summary. 45
Table S21 | Capping (Module c) summary. 46
Table S22 | Fmoc deprotection with NEt_3 (Module d) summary. 46
Table S23 | Levulinoyl ester deprotection (Module e) summary. 47
Table S24 | Compound synthesis summary. 49
1.5) Mouse lung MALDI-FTICR data analysis 54
1.6) *Macrocystis pyrifera* fucoidan digests in detail 55

2. Isomer calculation 57

Supplementary references 59

Supplementary Tables

Table S1 | Rules on combinations of modifications on a monomer in GlycoAnnotateR. Square matrix summarising which modifications are 'allowed' (x) together on a monomer or not (empty), according to the chemical rules outlined in the Supplementary Information. Sulfate is the only modification which can occur twice on the same monomer, using the special parameter 'double_sulfate'. Pentose and sialic acid are considered monomers, not modifications, but they are included as not all modifications are 'allowed' on these monomers.

	Pentose	Un-saturated	Sulfate	Phosphate	Carboxylic acid	Anhydro-bridge	O-Methyl	O-Acetyl	N-Acetyl	Amino	Deoxy	Dehydrated	Alditol	Sialic acid
Pentose	NA	x	x	x	x		x	x	x	x		x	x	
Un-saturated	x		x	x	x		x	x			x			
Sulfate	x	x	x	x	x	x	x	x	x	x	x	x	x	
Phosphate	x	x	x				x	x	x	x	x	x	x	
Carboxylic acid	x	x	x				x	x	x	x		x		
Anhydro-bridge			x				x		x					
O-Methyl	x	x	x	x	x	x		x	x	x	x	x	x	
O-Acetyl	x	x	x	x	x		x		x		x	x	x	
N-Acetyl	x		x	x	x	x	x	x		x	x		x	
Amino	x		x	x	x		x		x		x		x	
Deoxy		x	x	x			x	x	x	x		x		
Dehydrated	x		x	x	x		x	x			x			
Alditol	x		x	x			x	x	x	x				
Sialic acid														NA

Table S2 | Commercial mono- and oligosaccharide standards used in the standard mixture.

Common name	IUPAC composition name	Sum formula	Monoisotopic mass (Da)	Company
D-Mannitol	Hex1 Alditol1	C6H14O6	182.0790	Sigma Aldrich
L-Fucose	DeoxyHex1	C6H12O5	164.0685	Carbosynth
Galactose	Hex1	C6H12O6	180.0634	Sigma Aldrich
D-Galacturonic acid	Hex1 CarboxylicAcid1	C6H10O7	194.0427	Sigma Aldrich
D-Glucosamine	Hex1 Amino1	C6H13NO5	179.0794	Sigma Aldrich
6-O-methyl-D-Galactose	Hex1 O-Methyl1	C7H14O6	194.0790	Biosynth
N-acetylglucosamine	Hex1 N-Acetyl1	C8H15NO6	221.0899	Sigma Aldrich
Mannose-6-sulfate	Hex1 Sulfate1	C6H12O9S	260.0202	Sigma Aldrich
1,6-anhydro-glucose	Hex1 Anhydro1	C6H10O5	162.0528	Sigma Aldrich
Laminaribiose	Hex2	C12H22O11	342.1162	Megazyme
Laminaritriose	Hex3	C18H32O16	504.1690	Megazyme
Laminaritetraose	Hex4	C24H42O21	666.2218	Megazyme
Laminaripentaose	Hex5	C30H52O26	828.2746	Megazyme
Laminarihexaose	Hex6	C36H62O31	990.3275	Megazyme
κ-carrageenan DP2	Hex2 AnhydroBridge1 Sulfate1	C12H20O13S1	404.0625	DeXtra Labs
κ-carrageenan DP4	Hex4 AnhydroBridge2 Sulfate2	C24H38O25S2	790.1144	DeXtra Labs

Table S3 | Commercial standards DDA inclusion list

Mass (*m/z*)	Polarity
217.04255	Negative
199.03733	Negative
215.03225	Negative
193.03483	Negative
214.04823	Negative
229.04789	Negative
185.02168	Negative
256.05879	Negative
259.0129	Negative
197.02168	Negative
377.08507	Negative
539.13789	Negative

701.19072	Negative
863.24354	Negative
1025.29637	Negative
403.05519	Negative
394.04991	Negative
205.06347	Positive
187.05825	Positive
203.05317	Positive
217.03243	Positive
202.06914	Positive
180.0872	Positive
217.06881	Positive
173.04259	Positive
244.07971	Positive
282.00215	Positive
185.04259	Positive
365.10599	Positive
527.15881	Positive
689.21164	Positive
851.26446	Positive
1013.31729	Positive
427.05227	Positive
813.10417	Positive

Table S4 | Timed inclusion list for targeted selected ion monitoring (tSIM) with ddMS2 of *M. pyrifera* fucoidan digest.

**Mass (*m/z*)**	**Polarity**	**Start (min)**	**End (min)**
234.0127	Negative	1	2
243.0180	Negative	0.6	1.7
273.9911	Negative	2.5	3.5
313.9695	Negative	5	7
386.9985	Negative	5	9
426.9769	Negative	9	12
466.9553	Negative	13	15
500.0059	Negative	8	12
539.9843	Negative	12	14

579.9627	Negative	15	16
613.0132	Negative	12	14
652.9916	Negative	14	16
692.9700	Negative	17	18
765.9990	Negative	18	19

Table S5 | The 27 compositions that are possible with pentose and hexose monomers, amino and O-methyl modifications, and a DP of 2. Output of glycoPredict function from GlycoAnnotateR.

Monoisotopic mass (Da)	Formula	IUPAC name	GlyConnect ID	GlyTouCan ID
280.12705	C10H20N2O7	Pen2 Amino2	none	none
281.11107	C10H19N1O8	Pen2 Amino1	none	none
282.09508	C10H18O9	Pen2	108	G10326NC
294.14270	C11H22N2O7	Pen2 Amino2 O-Methyl1	none	none
295.12672	C11H21N1O8	Pen2 Amino1 O-Methyl1	none	none
296.11073	C11H20O9	Pen2 O-Methyl1	none	none
308.15835	C12H24N2O7	Pen2 Amino2 O-Methyl2	none	none
309.14237	C12H23N1O8	Pen2 Amino1 O-Methyl2	none	none
310.12638	C12H22O9	Pen2 O-Methyl2	none	none
310.13762	C11H22N2O8	Hex1 Pen1 Amino2	none	none
311.12163	C11H21N1O9	Hex1 Pen1 Amino1	none	none
312.10565	C11H20O10	Hex1 Pen1	91	G28916LJ
324.15327	C12H24N2O8	Hex1 Pen1 Amino2 O-Methyl1	none	none
325.13728	C12H23N1O9	Hex1 Pen1 Amino1 O-Methyl1	none	none
326.12130	C12H22O10	Hex1 Pen1 O-Methyl1	none	none
338.16892	C13H26N2O8	Hex1 Pen1 Amino2 O-Methyl2	none	none
339.15293	C13H25N1O9	Hex1 Pen1 Amino1 O-Methyl2	none	none
340.13695	C13H24O10	Hex1 Pen1 O-Methyl2	none	none
340.14818	C12H24N2O9	Hex2 Amino2	none	none
341.13220	C12H23N1O10	Hex2 Amino1	none	none
342.11621	C12H22O11	Hex2	136	G07133YY
354.16383	C13H26N2O9	Hex2 Amino2 O-Methyl1	none	none
355.14785	C13H25N1O10	Hex2 Amino1 O-Methyl1	none	none
356.13186	C13H24O11	Hex2 O-Methyl1	none	none
368.17948	C14H28N2O9	Hex2 Amino2 O-Methyl2	none	none
369.16350	C14H27N1O10	Hex2 Amino1 O-Methyl2	none	none
370.14751	C14H26O11	Hex2 O-Methyl2	none	none

Table S6 | Commercial mono- and oligosaccharides standards annotated in LC-MS data.

Standard molecule	Annotation	*m/z*						Mean retention time (s)	Class
		$[M+Na]^+$	$[M-H]^-$	$[M+Cl]^-$	$[M+K]^+$	$[M+H]^+$	$[M-2H]^{-2}$		
	Alditol1 DeoxyHex1	189.073						82.5	Noise or contamination
D-mannitol	Alditol1 Hex1	205.068	181.071	217.048	221.042			195.3	Standard peak
	Alditol1 Hex1 Amino1	204.087						149.5	Noise or contamination
Fucose	DeoxyHex1	187.058						240.6	Standard peak
Galactose	Hex1	203.053		215.032	219.027			215.4	Standard peak
Galactosamine	Hex1 Amino1					180.087		820.7	Standard peak
1,6-anhydro-glucose	Hex1 Anhydro1	185.042						63.3	Standard peak
N-acetylglucosamine	Hex1 N-Acetyl1	244.079			260.053	222.097		152.0	Standard peak
6-O-methylgalactose	Hex1 O-Methyl1	217.068		229.048	233.042			90.9	Standard peak
	Hex1 Sulfate1		259.013					66.4	Noise or contamination
	Hex1 Sulfate1		259.013					110.8	Insource (κ-carrageenan DP2)
Mannose-6-sulfate	Hex1 Sulfate1	283.009	259.013		298.983			189.1	Standard
	Hex2	365.105						402.6	Peak tail
	Hex2	365.105	341.108	377.085		343.123		462.8	Standard peak
Laminaribiose	Hex2	365.105						601.4	Peak tail
	Hex2 Anhydro1				363.068			104.3	Insource (κ-carrageenan DP2)
	Hex2 Anhydro1				363.068			137	Insource (κ-carrageenan DP2)
	Hex2 Anhydro1	347.094			363.089			170.8	Noise or contamination
	Hex2 Anhydro1	347.095						226.6	Noise or contamination
	Hex2 Anhydro1					325.112		462.6	Insource (Hex2)
κ-carrageenan DP2	Hex2 Anhydro1 Sulfate1		403.055					103.8	Standard peak
κ-carrageenan DP2	Hex2 Anhydro1 Sulfate1	427.051	403.01		443.025			137.4	Standard peak
	Hex2 Anhydro2					307.102		137.9	Insource (κ-carrageenan DP2)
Laminaritriose	Hex3	527.158	503.162		543.132			779.1	Standard peak
	Hex3 Anhydro1	509.147						400.9	Noise or contamination
	Hex3 Anhydro1 Sulfate1		565.109					468.9	Insource (κ-carrageenan DP4)
	Hex3 Anhydro2	491.137						137.9	Insource (κ-carrageenan DP2)
Laminaritetraose	Hex4	689.21			705.184			1059.2	Standard peak
	Hex4 Anhydro1	671.2						693	Noise or contamination
κ-carrageenan DP4	Hex4 Anhydro2 Sulfate2						394.006	487.1	Standard peak
Laminaripentaose	Hex5	851.263						1290.6	Standard peak
Laminarihexaose	Hex6	1013.316						1476.9	Standard peak

Table S7 | 18 distinct mono- and oligosaccharides annotated in LC-MS data of *M. pyrifera* digest. No GlycConnect or GlyToucan IDs were found so these columns are not shown. Molecule mass is the monoisotopic mass of neutral molecules. DP = degree of polymerization.

DP	Molecule mass (Da)	Neutral formula	IUPAC name	Ion	Predicted *m/z*	Ion formula	Ion charge	Median peak *m/z*	Median retention time (s)
1	244.025	C6H12O8S	DeoxyHex1 Sulfate1	$[M-H]^-$	243.0180	C6H11O8S	-1	243.018	59
1	323.982	C6H12O11S2	DeoxyHex1 Sulfate2	$[M-H]^-$	322.9748	C6H11O11S2	-1	322.975	83
1	323.982	C6H12O11S2	DeoxyHex1 Sulfate2	$[M-H]^-$	322.9748	C6H11O11S2	-1	322.975	405
2	470.040	C12H22O15S2	DeoxyHex2 Sulfate2	$[M-2H]^{-2}$	234.0127	C12H20O15S2	-2	234.013	90
2	549.997	C12H22O18S3	DeoxyHex2 Sulfate3	$[M-2H]^{-2}$	273.9911	C12H20O18S3	-2	273.992	180
2	629.954	C12H22O21S4	DeoxyHex2 Sulfate4	$[M-2H]^{-2}$	313.9695	C12H20O21S4	-2	313.969	409
3	776.012	C18H32O25S4	DeoxyHex3 Sulfate4	$[M-2H]^{-2}$	386.9985	C18H30O25S4	-2	386.999	402
3	776.012	C18H32O25S4	DeoxyHex3 Sulfate4	$[M-2H]^{-2}$	386.9985	C18H30O25S4	-2	386.999	360
3	855.968	C18H32O28S5	DeoxyHex3 Sulfate5	$[M-2H]^{-2}$	426.9769	C18H30O28S5	-2	426.977	641
3	855.968	C18H32O28S5	DeoxyHex3 Sulfate5	$[M-2H]^{-2}$	426.9769	C18H30O28S5	-2	426.977	590
3	935.925	C18H32O31S6	DeoxyHex3 Sulfate6	$[M-2H]^{-2}$	466.9553	C18H30O31S6	-2	466.956	839
4	922.069	C24H42O29S4	DeoxyHex4 Sulfate4	$[M-2H]^{-2}$	460.0275	C24H40O29S4	-2	460.028	433
4	1002.026	C24H42O32S5	DeoxyHex4 Sulfate5	$[M-2H]^{-2}$	500.0059	C24H40O32S5	-2	500.007	533
4	1002.026	C24H42O32S5	DeoxyHex4 Sulfate5	$[M-2H]^{-2}$	500.0059	C24H40O32S5	-2	500.007	684
4	1002.026	C24H42O32S5	DeoxyHex4 Sulfate5	$[M-2H]^{-2}$	500.0059	C24H40O32S5	-2	500.007	565
4	1081.983	C24H42O35S6	DeoxyHex4 Sulfate6	$[M-2H]^{-2}$	539.9843	C24H40O35S6	-2	539.986	801
5	1307.998	C30H52O42S7	DeoxyHex5 Sulfate7	$[M-2H]^{-2}$	652.9916	C30H50O42S7	-2	652.994	1054
5	1387.955	C30H52O45S8	DeoxyHex5 Sulfate8	$[M-2H]^{-2}$	692.9700	C30H50O45S8	-2	692.972	1045

Table S8 | Annotated fragment ions from MS/MS of DeoxyHex1 Sulfate1 [M-H]- from 50-70 s. Data was obtained from targeted selected ion monitoring with data-dependent MS/MS. Peaks were combined within spectra and then averaged across spectra. Precursor *m/z* was 243.018, isolated with a window of 0.4 *m/z*. Intensities were normalised with respect to the maximum ion intensity.

m/z	Normalised intensity (%)	Annotation
67.622	0.83	
96.9588	6.27	HSO4-
97.2079	0.82	
116.9274	1.17	
138.9696	1.05	
152.9117	0.58	
162.9091	3.03	
182.9962	3.51	
196.8765	4.08	
196.9017	0.63	
198.8638	0.62	
214.9122	0.90	
224.9502	0.90	
225.113	0.53	
225.1492	0.56	
242.7935	0.91	
242.873	2.62	
242.9604	1.51	
243.002	0.65	
243.0179	100.00	DeoxyHex1 Sulfate1:[M-H]-
243.0872	0.64	
243.1237	1.22	
243.1601	2.90	
243.1965	0.59	

Table S9 | Annotated fragment ions from MS/MS of DeoxyHex2 Sulfate [M-2H]-2 from 400-414 s. Data was obtained from targeted selected ion monitoring with data-dependent MS/MS. Peaks were combined within spectra and then averaged across spectra. Precursor *m/z* was 313.969, isolated with a window of 0.4 *m/z*. The precursor ion is not present in the average mass spectrum. Intensities were normalised with respect to the maximum ion intensity.

m/z	Normalised intensity (%)	Annotations
61.9869	100.00	
67.622	2.23	
96.9588	4.31	HSO4-
97.208	1.76	
225.0072	5.55	Dehydrated DeoxyHex1 Sulfate1:[M-H]-, Dehydrated DeoxyHex2 Sulfate2:[M-2H]-2
232.9248	2.10	
234.0014	0.72	
234.0126	26.92	DeoxyHex2 Sulfate2:[M-2H]-2
234.0248	1.12	
243.018	6.85	DeoxyHex1 Sulfate1:[M-H]-
267.8808	1.29	
269.8961	1.52	
273.9916	1.37	DeoxyHex2 Sulfate3:[M-2H]-2
277.877	1.35	
285.8907	3.82	
295.8866	10.64	
304.9642	2.34	Dehydrated DeoxyHex1 Sulfate2:[M-H]-, Dehydrated DeoxyHex2 Sulfate4:[M-2H]-2
313.8529	0.63	
313.9307	1.36	
371.0656	2.29	Dehydrated DeoxyHex2 Sulfate1:[M-H]-
423.7725	0.67	
451.023	1.24	Dehydrated DeoxyHex2 Sulfate2:[M-H]-

Table S10 | Annotated fragment ions from MS/MS of DeoxyHex3 Sulfate5 [M-2H]-2 from 580-613 s. Data was obtained from targeted selected ion monitoring with data-dependent MS/MS. Peaks were combined within spectra and then averaged across spectra. Precursor *m/z* was 426.977, isolated with a window of 0.4 *m/z*. Intensities were normalised with respect to the maximum ion intensity.

m/z	**Normalised intensity (%)**	**Annotations**
67.6219	20.622	
96.9587	11.972	HSO4-
97.208	14.195	
114.9329	9.238	
225.0071	11.265	Dehydrated DeoxyHex1 Sulfate1:[M-H]-, Dehydrated DeoxyHex2 Sulfate2:[M-2H]-2, Dehydrated DeoxyHex3 Sulfate3:[M-3H]-3
243.0179	4.983	DeoxyHex1 Sulfate1:[M-H]-
307.0411	81.628	DeoxyHex3 Sulfate2:[M-2H]-2
347.0196	86.366	DeoxyHex3 Sulfate3:[M-2H]-2
362.8761	6.743	
382.8173	5.686	
398.8119	9.219	
426.7635	5.420	
426.7881	100.000	DeoxyHex3 Sulfate5:[M-2H]-2
426.8171	5.788	
426.841	7.491	
469.0326	7.980	DeoxyHex2 Sulfate2:[M-H]-
864.8371	4.171	

Table S11 | Annotated fragment ions from MS/MS of DeoxyHex3 Sulfate5 [M-2H]-2 from 614-650 s. Data was obtained from targeted selected ion monitoring with data-dependent MS/MS. Peaks were combined within spectra and then averaged across spectra. Precursor *m/z* was 426.976, isolated with a window of 0.4 *m/z*. Intensities were normalised with respect to the maximum ion intensity.

m/z	Normalised intensity (%)	Annotations
67.622	20.84	
96.9588	6.12	HSO4-
97.208	13.62	
114.9329	8.22	
225.0071	22.54	Dehydrated DeoxyHex1 Sulfate1:[M-H]-, Dehydrated DeoxyHex2 Sulfate2:[M-2H]-2, Dehydrated DeoxyHex3 Sulfate3:[M-3H]-3
243.0179	5.55	DeoxyHex1 Sulfate1:[M-H]-
273.9913	12.30	DeoxyHex2 Sulfate3:[M-2H]-2
307.0412	18.98	DeoxyHex3 Sulfate2:[M-2H]-2
347.0195	20.43	DeoxyHex3 Sulfate3:[M-2H]-2
362.8758	4.41	
382.8169	6.89	
398.8121	9.83	
426.7638	5.93	
426.7881	100.00	DeoxyHex3 Sulfate5:[M-2H]-2
426.8408	5.93	
469.0328	10.37	DeoxyHex2 Sulfate2:[M-H]-

Table S12 | Annotated fragment ions from MS/MS of DeoxyHex3 Sulfate6 [M-2H]-2 from 820-852 s. Data was obtained from targeted selected ion monitoring with data-dependent MS/MS. Peaks were combined within spectra and then averaged across spectra. Precursor *m/z* was 466.955, isolated with a window of 0.4 *m/z*. The precursor ion is not present in the average mass spectrum. Intensities were normalised with respect to the maximum ion intensity.

m/z	Normalised intensity (%)	Annotations
67.622	49.40	
97.2079	31.29	
293.1769	10.60	
307.0408	25.19	DeoxyHex3 Sulfate2:[M-2H]-2
347.0196	100.00	DeoxyHex3 Sulfate3:[M-2H]-2
386.9982	59.92	DeoxyHex3 Sulfate4:[M-2H]-2
420.8146	12.50	
425.7625	12.45	

Table S13 | Annotated fragment ions from MS/MS of DeoxyHex4 Sulfate5 [M-2H]-2 from 661-686 s. Data was obtained from targeted selected ion monitoring with data-dependent MS/MS. Peaks were combined within spectra and then averaged across spectra. Precursor *m/z* was 500.006, isolated with a window of 0.4 *m/z*. The precursor ion is not present in the average mass spectrum. Intensities were normalised with respect to the maximum ion intensity.

m/z	Normalised intensity (%)	Annotations
97.208	100.00	
225.007	30.35	Dehydrated DeoxyHex1 Sulfate1:[M-H]-, Dehydrated DeoxyHex2 Sulfate2:[M-2H]-2, Dehydrated DeoxyHex4 Sulfate4:[M-4H]-4, Dehydrated DeoxyHex3 Sulfate3:[M-3H]-3
243.0179	44.46	DeoxyHex1 Sulfate1:[M-H]-
380.0701	56.02	DeoxyHex4 Sulfate2:[M-2H]-2
420.049	75.78	DeoxyHex4 Sulfate3:[M-2H]-2
481.8123	72.17	

Table S14 | Annotated fragment ions from MS/MS of DeoxyHex4 Sulfate6 [M-2H]-2 from 754-816 s. Data was obtained from targeted selected ion monitoring with data-dependent MS/MS. Peaks were combined within spectra and then averaged across spectra. Precursor *m/z* was 539.982, isolated with a window of 0.4 *m/z*. The precursor ion is not present in the average mass spectrum. Intensities were normalised with respect to the maximum ion intensity.

m/z	Normalised intensity (%)	Annotations
96.9588	8.07	HSO4-
97.208	23.85	
225.0071	13.07	Dehydrated DeoxyHex1 Sulfate1:[M-H]-, Dehydrated DeoxyHex2 Sulfate2:[M-2H]-2, Dehydrated DeoxyHex4 Sulfate4:[M-4H]-4, Dehydrated DeoxyHex3 Sulfate3:[M-3H]-3
380.0697	22.39	DeoxyHex4 Sulfate2:[M-2H]-2
420.0491	74.57	DeoxyHex4 Sulfate3:[M-2H]-2
460.0276	100.00	DeoxyHex4 Sulfate4:[M-2H]-2
500.0069	11.90	DeoxyHex4 Sulfate5:[M-2H]-2

Table S15 | Annotated fragment ions from MS/MS of DeoxyHex5 Sulfate8 $[M\text{-}2H]^{-2}$ from 1044-1045 s. Data was obtained from targeted selected ion monitoring with data-dependent MS/MS. Peaks were combined within spectra and then averaged across spectra. Precursor *m/z* was 692.969, isolated with a window of 0.4 *m/z*. The precursor ion is not present in the average mass spectrum. Intensities were normalised with respect to the maximum ion intensity.

m/z	Normalised intensity (%)	Annotations
97.2079	100.00	
102.8068	12.17	
105.2339	12.41	
110.3104	12.08	
143.3316	13.24	
168.4991	14.68	
171.8473	12.21	
179.3711	11.51	
208.5592	11.41	
212.5205	11.75	
215.1255	12.01	
221.4815	12.34	
262.5267	13.01	
453.4936	13.98	
526.1552	13.20	
533.0568	21.42	DeoxyHex5 Sulfate4:[M-2H]-2
573.0349	19.79	DeoxyHex5 Sulfate5:[M-2H]-2
760.0112	15.72	
779.5659	15.25	
838.8362	15.18	
862.9735	15.88	
864.8633	14.13	
1248.2856	15.94	

Table S16 | GlycoAnnotateR annotation of NGlycDB annotations for mouse lung MALDI-MSI data downloaded from METASPACE. Dataset: 20210401_lung_p27_1; NGlycDB annotations at FDR of 5%. Table with 88 annotations was downloaded from METASPACE in csv format. GlycoAnnotateR was used to annotate peaks based on the *m/z* values with a mass tolerance of 3 ppm. Where multiple annotations were assigned to the same peak by GlycoAnnotateR, the annotation that matches the NGlycDB annotation is indicated by an asteriks (*). The 4 peaks with no annotation from GlycoAnnotateR are bolded; all 4 NGlycDB annotations contain pentose, which was not accounted for in GlycoAnnotateR annotations as mammalian glycans do not contain pentose.

m/z	**NGlycDB annotation**	**GlycoAnnotateR annotation**	**Mass deviation (ppm)**
1079.3749	Hex:3 HexNAc:2 dHex:1	Hex5 DeoxyHex1 N-Acetyl2 [M+Na]+	0.009
1095.3698	Hex:4 HexNAc:2	Hex6 N-Acetyl2 [M+Na]+	0.009
1136.3964	Hex:3 HexNAc:3	Hex6 N-Acetyl3 [M+Na]+	0.009
1257.4226	Hex:5 HexNAc:2	Hex7 N-Acetyl2 [M+Na]+	0.009
1282.4543	Hex:3 HexNAc:3 dHex:1	Hex6 DeoxyHex1 N-Acetyl3 [M+Na]+	0.009
1298.4492	Hex:4 HexNAc:3	Hex7 N-Acetyl3 [M+Na]+	0.009
1339.4757	Hex:3 HexNAc:4	Hex7 N-Acetyl4 [M+Na]+	0.009
1419.4755	Hex:6 HexNAc:2	Hex8 N-Acetyl2 [M+Na]+	0.010
1444.5071	Hex:4 HexNAc:3 dHex:1	Hex7 DeoxyHex1 N-Acetyl3 [M+Na]+	0.009
1460.5020	Hex:5 HexNAc:3	Hex8 N-Acetyl3 [M+Na]+	0.009
1485.5337	Hex:3 HexNAc:4 dHex:1	Hex7 DeoxyHex1 N-Acetyl4 [M+Na]+	0.009
1501.5286	Hex:4 HexNAc:4	Hex8 N-Acetyl4 [M+Na]+	0.009
1542.5551	Hex:3 HexNAc:5	Hex8 N-Acetyl5 [M+Na]+	0.009
**1546.5388**	**Hex:3 HexNAc:3 dHex:1 Pent:2**		
1581.5283	Hex:7 HexNAc:2	Hex9 N-Acetyl2 [M+Na]+	0.010
1606.5599	Hex:5 HexNAc:3 dHex:1	Hex8 DeoxyHex1 N-Acetyl3 [M+Na]+	0.009
1622.5548	Hex:6 HexNAc:3	Hex9 N-Acetyl3 [M+Na]+	0.009
1647.5865	Hex:4 HexNAc:4 dHex:1	Hex8 DeoxyHex1 N-Acetyl4 [M+Na]+	0.009
**1648.5943**	**Hex:4 HexNAc:4 Pent:1 Me:1**		
1663.5814	Hex:5 HexNAc:4	Hex9 N-Acetyl4 [M+Na]+	0.009
**1663.6178**	**Hex:4 HexNAc:4 Pent:1 Me:2**		
1688.6130	Hex:3 HexNAc:5 dHex:1	Hex8 DeoxyHex1 N-Acetyl5 [M+Na]+	0.009
1704.6079	Hex:4 HexNAc:5	Hex9 N-Acetyl5 [M+Na]+	0.009
1743.5811	Hex:8 HexNAc:2	Hex10 N-Acetyl2 [M+Na]+	0.010
1749.6182	Hex:3 HexNAc:4 dHex:1 Pent:2	Hex7 DeoxyHex1 NeuAc1 N-Acetyl3 O-Methyl1 [M+Na]+	0.636
1768.6128	Hex:6 HexNAc:3 dHex:1	Hex9 DeoxyHex1 N-Acetyl3 [M+Na]+	0.009
1784.6077	Hex:7 HexNAc:3	Hex10 N-Acetyl3 [M+Na]+	0.009
1793.6444	Hex:4 HexNAc:4 dHex:2	Hex8 DeoxyHex2 N-Acetyl4 [M+Na]+	0.009

1794.6522	**Hex:4 HexNAc:4 dHex:1 Pent:1 Me:1**		
1809.6393	Hex:5 HexNAc:4 dHex:1	Hex9 DeoxyHex1 N-Acetyl4 [M+Na]+	0.009
1825.6342	Hex:6 HexNAc:4	Hex10 N-Acetyl4 [M+Na]+	0.009
1850.6659	Hex:4 HexNAc:5 dHex:1	Hex9 DeoxyHex1 N-Acetyl5 [M+Na]+	0.009
1866.6608	Hex:5 HexNAc:5	Hex10 N-Acetyl5 [M+Na]+	0.009
1891.6924	Hex:3 HexNAc:6 dHex:1	Hex9 DeoxyHex1 N-Acetyl6 [M+Na]+	0.008
1905.6339	Hex:9 HexNAc:2	Hex11 N-Acetyl2 [M+Na]+	0.010
1955.6972	Hex:5 HexNAc:4 dHex:2	Hex9 DeoxyHex2 N-Acetyl4 [M+Na]+	0.009
1971.6492	Hex:3 HexNAc:6 dHex:1 Su:1	Hex4 NeuAc4 N-Acetyl2 O-Methyl1 [M-H+2Na]+	2.365
		Hex9 DeoxyHex1 N-Acetyl6 Sulfate1 [M+Na]+*	0.566
1971.6921	Hex:6 HexNAc:4 dHex:1	Hex10 DeoxyHex1 N-Acetyl4 [M+Na]+	0.009
1976.6588	Hex:5 HexNAc:4 NeuAc:1 -H+Na	Hex9 NeuAc1 N-Acetyl4 [M-H+2Na]+*	2.196
		Hex7 DeoxyHex4 N-Acetyl3 O-Methyl1 Sulfate1 [M+Na]+	0.550
		Hex11 N-Acetyl1 O-Acetyl2 O-Methyl2 [M+Na]+	0.564
1987.6870	Hex:7 HexNAc:4	Hex11 N-Acetyl4 [M+Na]+	0.009
1996.7238	Hex:4 HexNAc:5 dHex:2	Hex9 DeoxyHex2 N-Acetyl5 [M+Na]+	0.009
2012.7187	Hex:5 HexNAc:5 dHex:1	Hex10 DeoxyHex1 N-Acetyl5 [M+Na]+	0.009
2028.7136	Hex:6 HexNAc:5	Hex11 N-Acetyl5 [M+Na]+	0.009
2037.7503	Hex:3 HexNAc:6 dHex:2	Hex9 DeoxyHex2 N-Acetyl6 [M+Na]+	0.008
2040.7136	Hex:5 HexNAc:4 NeuAc:1 Ac:2	Hex7 DeoxyHex1 NeuAc2 N-Acetyl3 O-Methyl1 [M+Na]+	0.546
2053.7452	Hex:4 HexNAc:6 dHex:1	Hex10 DeoxyHex1 N-Acetyl6 [M+Na]+	0.009
2067.6868	Hex:10 HexNAc:2	Hex12 N-Acetyl2 [M+Na]+	0.010
2100.7347	Hex:5 HexNAc:4 dHex:1 NeuAc:1	Hex9 DeoxyHex1 NeuAc1 N-Acetyl4 [M+Na]+	0.531
2116.7296	Hex:6 HexNAc:4 NeuAc:1	Hex10 NeuAc1 N-Acetyl4 [M+Na]+	0.527
2122.7167	Hex:5 HexNAc:4 dHex:1 NeuAc:1 -H+Na	Hex9 DeoxyHex1 NeuAc1 N-Acetyl4 [M-H+2Na]+*	2.044
		Hex7 DeoxyHex5 N-Acetyl3 O-Methyl1 Sulfate1 [M+Na]+	0.513
		Hex11 DeoxyHex1 N-Acetyl1 O-Acetyl2 O-Methyl2 [M+Na]+	0.526
2133.7449	Hex:7 HexNAc:4 dHex:1	Hex6 NeuAc3 N-Acetyl5 O-Acetyl1 [M+Na]+	0.009
		Hex11 DeoxyHex1 N-Acetyl4 [M+Na]+*	1.149
2138.7116	Hex:6 HexNAc:4 NeuAc:1 -H+Na	Hex10 NeuAc1 N-Acetyl4 [M-H+2Na]+*	2.029
		Hex8 DeoxyHex4 N-Acetyl3 O-Methyl1 Sulfate1 [M+Na]+	0.509
		Hex12 N-Acetyl1 O-Acetyl2 O-Methyl2 [M+Na]+	0.522
2158.7766	Hex:5 HexNAc:5 dHex:2	Hex10 DeoxyHex2 N-Acetyl5 [M+Na]+	0.009
2174.7286	Hex:3 HexNAc:7 dHex:1 Su:1	Hex5 NeuAc4 N-Acetyl3 O-Methyl1 [M-H+2Na]+	2.143
		Hex10 DeoxyHex1 N-Acetyl7 Sulfate1 [M+Na]+*	0.513
2174.7715	Hex:6 HexNAc:5 dHex:1	Hex11 DeoxyHex1 N-Acetyl5 [M+Na]+	0.009

2183.8082	Hex:3 HexNAc:6 dHex:3	Hex9 DeoxyHex3 N-Acetyl6 [M+Na]+	0.008
2190.7664	Hex:7 HexNAc:5	Hex12 N-Acetyl5 [M+Na]+	0.009
2215.7980	Hex:5 HexNAc:6 dHex:1	Hex11 DeoxyHex1 N-Acetyl6 [M+Na]+	0.009
2231.7930	Hex:6 HexNAc:6	Hex12 N-Acetyl6 [M+Na]+	0.009
2278.7825	Hex:7 HexNAc:4 NeuAc:1	Hex11 NeuAc1 N-Acetyl4 [M+Na]+	0.490
2300.7644	Hex:7 HexNAc:4 NeuAc:1 -H+Na	Hex11 NeuAc1 N-Acetyl4 [M-H+2Na]+*	1.885
		Hex9 DeoxyHex4 N-Acetyl3 O-Methyl1 Sulfate1 [M+Na]+	0.474
		Hex13 N-Acetyl1 O-Acetyl2 O-Methyl2 [M+Na]+	0.486
2304.8345	Hex:5 HexNAc:5 dHex:3	Hex10 DeoxyHex3 N-Acetyl5 [M+Na]+	0.009
2320.8294	Hex:6 HexNAc:5 dHex:2	Hex11 DeoxyHex2 N-Acetyl5 [M+Na]+	0.009
2336.8243	Hex:7 HexNAc:5 dHex:1	Hex7 NeuAc3 N-Acetyl6 O-Acetyl1 [M+Na]+	0.009
		Hex12 DeoxyHex1 N-Acetyl5 [M+Na]+*	1.050
2352.8192	Hex:8 HexNAc:5	Hex13 N-Acetyl5 [M+Na]+	0.009
2377.8509	Hex:6 HexNAc:6 dHex:1	Hex12 DeoxyHex1 N-Acetyl6 [M+Na]+	0.009
2393.8458	Hex:7 HexNAc:6	Hex13 N-Acetyl6 [M+Na]+	0.009
2465.8669	Hex:6 HexNAc:5 dHex:1 NeuAc:1	Hex11 DeoxyHex1 NeuAc1 N-Acetyl5 [M+Na]+	0.454
2481.8618	Hex:7 HexNAc:5 NeuAc:1	Hex12 NeuAc1 N-Acetyl5 [M+Na]+	0.451
2487.8489	Hex:6 HexNAc:5 dHex:1 NeuAc:1 -H+Na	Hex11 DeoxyHex1 NeuAc1 N-Acetyl5 [M-H+2Na]+*	1.743
		Hex9 DeoxyHex5 N-Acetyl4 O-Methyl1 Sulfate1 [M+Na]+	0.439
		Hex13 DeoxyHex1 N-Acetyl2 O-Acetyl2 O-Methyl2 [M+Na]+	0.450
2498.8771	Hex:8 HexNAc:5 dHex:1	Hex8 NeuAc3 N-Acetyl6 O-Acetyl1 [M+Na]+	0.009
		Hex13 DeoxyHex1 N-Acetyl5 [M+Na]+*	0.983
2503.8438	Hex:7 HexNAc:5 NeuAc:1 -H+Na	Hex12 NeuAc1 N-Acetyl5 [M-H+2Na]+*	1.732
		Hex10 DeoxyHex4 N-Acetyl4 O-Methyl1 Sulfate1 [M+Na]+	0.436
		Hex14 N-Acetyl2 O-Acetyl2 O-Methyl2 [M+Na]+	0.447
2522.8884	Hex:6 HexNAc:6 NeuAc:1	Hex12 NeuAc1 N-Acetyl6 [M+Na]+	0.444
2523.9088	Hex:6 HexNAc:6 dHex:2	Hex12 DeoxyHex2 N-Acetyl6 [M+Na]+	0.009
2539.9037	Hex:7 HexNAc:6 dHex:1	Hex8 NeuAc3 N-Acetyl7 O-Acetyl1 [M+Na]+	0.009
		Hex13 DeoxyHex1 N-Acetyl6 [M+Na]+*	0.967
2580.9302	Hex:6 HexNAc:7 dHex:1	Hex13 DeoxyHex1 N-Acetyl7 [M+Na]+	0.009
2660.9300	Hex:9 HexNAc:5 dHex:1	Hex6 NeuAc5 N-Acetyl4 O-Methyl2 [M+Na]+	0.009
		Hex9 NeuAc3 N-Acetyl6 O-Acetyl1 [M+Na]+	0.923
		Hex14 DeoxyHex1 N-Acetyl5 [M+Na]+*	0.923
2669.9667	Hex:6 HexNAc:6 dHex:3	Hex12 DeoxyHex3 N-Acetyl6 [M+Na]+	0.009
2701.9565	Hex:8 HexNAc:6 dHex:1	Hex9 NeuAc3 N-Acetyl7 O-Acetyl1 [M+Na]+	0.009
		Hex14 DeoxyHex1 N-Acetyl6 [M+Na]+*	0.909

2742.9831	Hex:7 HexNAc:7 dHex:1	Hex9 NeuAc3 N-Acetyl8 O-Acetyl1 [M+Na]+	0.009
		Hex14 DeoxyHex1 N-Acetyl7 [M+Na]+*	0.896
2758.9780	Hex:8 HexNAc:7	Hex15 N-Acetyl7 [M+Na]+	0.009
2864.0093	Hex:9 HexNAc:6 dHex:1	Hex7 NeuAc5 N-Acetyl5 O-Methyl2 [M+Na]+	0.009
		Hex10 NeuAc3 N-Acetyl7 O-Acetyl1 [M+Na]+	0.858
		Hex15 DeoxyHex1 N-Acetyl6 [M+Na]+*	0.858
2889.0410	Hex:7 HexNAc:7 dHex:2	Hex9 DeoxyHex1 NeuAc3 N-Acetyl8 O-Acetyl1 [M+Na]+	0.009
		Hex14 DeoxyHex2 N-Acetyl7 [M+Na]+*	0.851
2905.0359	Hex:8 HexNAc:7 dHex:1	Hex10 NeuAc3 N-Acetyl8 O-Acetyl1 [M+Na]+	0.009
		Hex15 DeoxyHex1 N-Acetyl7 [M+Na]+*	0.846
3067.0887	Hex:9 HexNAc:7 dHex:1	Hex8 NeuAc5 N-Acetyl6 O-Methyl2 [M+Na]+	2.860
		Hex7 DeoxyHex3 NeuAc4 N-Acetyl5 O-Acetyl2 [M+Na]+	0.009
		Hex11 NeuAc3 N-Acetyl8 O-Acetyl1 [M+Na]+	0.802
		Hex16 DeoxyHex1 N-Acetyl7 [M+Na]+*	0.802
3108.1153	Hex:8 HexNAc:8 dHex:1	Hex11 NeuAc3 N-Acetyl9 O-Acetyl1 [M+Na]+	0.009
		Hex16 DeoxyHex1 N-Acetyl8 [M+Na]+*	0.791
3270.1681	Hex:9 HexNAc:8 dHex:1	Hex9 NeuAc5 N-Acetyl7 O-Methyl2 [M+Na]+	2.682
		Hex8 DeoxyHex3 NeuAc4 N-Acetyl6 O-Acetyl2 [M+Na]+	0.009
		Hex12 NeuAc3 N-Acetyl9 O-Acetyl1 [M+Na]+	0.753
		Hex17 DeoxyHex1 N-Acetyl8 [M+Na]+*	0.753
3635.3003	Hex:10 HexNAc:9 dHex:1	Hex11 NeuAc5 N-Acetyl8 O-Methyl2 [M+Na]+	2.780
		Hex10 DeoxyHex3 NeuAc4 N-Acetyl7 O-Acetyl2 [M+Na]+	2.780
		Hex14 NeuAc3 N-Acetyl10 O-Acetyl1 [M+Na]+	2.412
		Hex12 DeoxyHex4 NeuAc2 N-Acetyl9 O-Acetyl1 O-Methyl2 [M-2H+3Na]+	0.009
		Hex9 DeoxyHex6 NeuAc3 N-Acetyl8 O-Methyl1 [M-2H+3Na]+	0.461
		Hex12 DeoxyHex4 NeuAc3 N-Acetyl4 O-Methyl2 [M+Na]+	0.461
		Hex15 DeoxyHex4 NeuAc1 N-Acetyl6 O-Acetyl1 [M+Na]+	0.678
		Hex19 DeoxyHex1 N-Acetyl9 [M+Na]+*	0.678

Table S17 | Top-ranked peaks in segmentation class containing peaks localised to the outside edge of the mouse lung section. All ions with statistic values > 0 are shown.

m/z	Annotations
1197.14017	Hex5 O-Acetyl2 Sulfate3:[M-H+2Na]+
1350.24252	Hex6 N-Acetyl1 O-Acetyl1 O-Methyl1 Sulfate3:[M+Na]+
1372.22391	Hex6 N-Acetyl1 O-Acetyl1 O-Methyl1 Sulfate3:[M-H+2Na]+
1386.17966	Hex5 DeoxyHex1 N-Acetyl1 O-Acetyl1 Sulfate3:[M-3H+4Na]+
1388.1912	Hex6 N-Acetyl1 O-Methyl2 Sulfate3:[M-3H+4Na]+
1394.20302	Hex6 N-Acetyl1 O-Acetyl1 O-Methyl1 Sulfate3:[M-2H+3Na]+
1416.18566	Hex6 N-Acetyl1 O-Acetyl1 O-Methyl1 Sulfate3:[M-3H+4Na]+
1433.20551	Hex4 DeoxyHex3 Sulfate3:[M-3H+4Na]+
1557.24426	Hex7 O-Acetyl2 O-Methyl1 Sulfate3:[M-2H+3Na]+
1561.26331	Hex6 DeoxyHex1 N-Acetyl2 O-Methyl1 Sulfate3:[M-3H+4Na]+
1579.22553	Hex7 O-Acetyl2 O-Methyl1 Sulfate3:[M-3H+4Na]+
1583.27733	Hex7 DeoxyHex1 Sulfate3:[M-H+2Na]+
1584.24921	Hex7 N-Acetyl1 O-Acetyl2 Sulfate3:[M-2H+3Na]+
1606.23183	Hex7 N-Acetyl1 O-Acetyl2 Sulfate3:[M-3H+4Na]+
1627.24385	Hex7 DeoxyHex1 Sulfate3:[M-3H+4Na]+
1771.3186	Hex6 DeoxyHex3 O-Methyl1 Sulfate3:[M-3H+4Na]+
1914.4121	Hex9 N-Acetyl1 O-Acetyl2 O-Methyl2 Sulfate3:[M-H+2Na]+, Hex7 NeuAc1 N-Acetyl4 Sulfate3:[M-2H+3Na]+
1959.35259	Hex7 NeuAc1 N-Acetyl3 O-Acetyl1 Sulfate3:[M-4H+5Na]+
4023.04017	Hex14 NeuAc4 N-Acetyl3 O-Acetyl2 O-Methyl1 Sulfate3:[M-4H+5Na]+, Hex10 DeoxyHex4 NeuAc4 N-Acetyl5 O-Methyl1 Sulfate3:[M-7H+8Na]+, Hex13 DeoxyHex3 NeuAc3 N-Acetyl4 O-Methyl2 Sulfate3:[M-6H+7Na]+
2558.67617	Hex13 DeoxyHex1 N-Acetyl1 O-Acetyl1 Sulfate2:[M-H+2Na]+
1751.31004	Hex7 DeoxyHex1 N-Acetyl2 O-Acetyl1 Sulfate3:[M-3H+4Na]+
4703.63853	Hex15 DeoxyHex3 NeuAc4 N-Acetyl11 O-Acetyl2 O-Methyl2:[M-3H+4Na]+, Hex12 DeoxyHex5 NeuAc5 N-Acetyl10 O-Acetyl1 O-Methyl1:[M-3H+4Na]+, Hex12 DeoxyHex5 NeuAc5 N-Acetyl10 O-Acetyl1 Sulfate1:[M+Na]+, Hex15 DeoxyHex3 NeuAc4 N-Acetyl11 O-Acetyl2 O-Methyl1 Sulfate1:[M+Na]+, Hex19 NeuAc3 N-Acetyl14 O-Acetyl1 O-Methyl1 Sulfate1:[M+Na]+, Hex16 DeoxyHex2 NeuAc4 N-Acetyl13 Sulfate1:[M+Na]+

Table S18 | Peaks co-localised to outer edge of mouse lung section. Result of co-localisation with 1416.18566 *m/z* with Cardinal. Purple shaded rows represent one potential fragmentation network.

m/z	Correlation	Annotations
1416.186	1.000	Hex6 N-Acetyl1 O-Acetyl1 O-Methyl1 Sulfate3:[M-3H+4Na]+
1627.244	0.902	Hex7 DeoxyHex1 Sulfate3:[M-3H+4Na]+
1394.203	0.896	Hex6 N-Acetyl1 O-Acetyl1 O-Methyl1 Sulfate3:[M-2H+3Na]+
1433.206	0.848	Hex4 DeoxyHex3 Sulfate3:[M-3H+4Na]+
1959.353	0.803	Hex7 NeuAc1 N-Acetyl3 O-Acetyl1 Sulfate3:[M-4H+5Na]+
1606.232	0.795	Hex7 N-Acetyl1 O-Acetyl2 Sulfate3:[M-3H+4Na]+
1557.244	0.791	Hex7 O-Acetyl2 O-Methyl1 Sulfate3:[M-2H+3Na]+
1372.224	0.765	Hex6 N-Acetyl1 O-Acetyl1 O-Methyl1 Sulfate3:[M-H+2Na]+
1584.249	0.629	Hex7 N-Acetyl1 O-Acetyl2 Sulfate3:[M-2H+3Na]+
1197.140	0.622	Hex5 O-Acetyl2 Sulfate3:[M-H+2Na]+
1388.191	0.571	Hex6 N-Acetyl1 O-Methyl2 Sulfate3:[M-3H+4Na]+
1579.226	0.553	Hex7 O-Acetyl2 O-Methyl1 Sulfate3:[M-3H+4Na]+
1350.243	0.538	Hex6 N-Acetyl1 O-Acetyl1 O-Methyl1 Sulfate3:[M+Na]+
1771.319	0.458	Hex6 DeoxyHex3 O-Methyl1 Sulfate3:[M-3H+4Na]+
1561.263	0.448	Hex6 DeoxyHex1 N-Acetyl2 O-Methyl1 Sulfate3:[M-3H+4Na]+
1751.310	0.392	Hex7 DeoxyHex1 N-Acetyl2 O-Acetyl1 Sulfate3:[M-3H+4Na]+
1562.241	0.385	Hex6 DeoxyHex1 N-Acetyl1 O-Acetyl1 O-Methyl1 Sulfate3:[M-3H+4Na]+
1583.277	0.367	Hex7 DeoxyHex1 Sulfate3:[M-H+2Na]+
1604.271	0.350	Hex7 N-Acetyl3 Sulfate3:[M-3H+4Na]+
1386.180	0.333	Hex5 DeoxyHex1 N-Acetyl1 O-Acetyl1 Sulfate3:[M-3H+4Na]+
1582.289	0.325	Hex7 N-Acetyl3 Sulfate3:[M-2H+3Na]+
1914.412	0.298	Hex9 N-Acetyl1 O-Acetyl2 O-Methyl2 Sulfate3:[M-H+2Na]+, Hex7 NeuAc1 N-Acetyl4 Sulfate3:[M-2H+3Na]+
4023.040	0.283	Hex14 NeuAc4 N-Acetyl3 O-Acetyl2 O-Methyl1 Sulfate3:[M-4H+5Na]+, Hex10 DeoxyHex4 NeuAc4 N-Acetyl5 O-Methyl1 Sulfate3:[M-7H+8Na]+, Hex13 DeoxyHex3 NeuAc3 N-Acetyl4 O-Methyl2 Sulfate3:[M-6H+7Na]+
2127.417	0.273	Hex10 DeoxyHex1 O-Methyl1 Sulfate3:[M-3H+4Na]+
1936.393	0.255	Hex9 N-Acetyl1 O-Acetyl2 O-Methyl2 Sulfate3:[M-2H+3Na]+, Hex7 NeuAc1 N-Acetyl4 Sulfate3:[M-3H+4Na]+
1535.330	0.251	Hex5 DeoxyHex3 O-Acetyl1 Sulfate2:[M-2H+3Na]+
2558.676	0.248	Hex13 DeoxyHex1 N-Acetyl1 O-Acetyl1 Sulfate2:[M-H+2Na]+
1578.236	0.235	Hex7 N-Acetyl1 O-Acetyl1 O-Methyl1 Sulfate3:[M-3H+4Na]+
1625.263	0.234	Hex6 DeoxyHex2 O-Methyl1 Sulfate3:[M-3H+4Na]+
3653.032	0.233	Hex10 DeoxyHex1 NeuAc5 N-Acetyl4 O-Methyl1 Sulfate1:[M-6H+7Na]+, Hex10 DeoxyHex1 NeuAc5 N-Acetyl4 Sulfate2:[M-3H+4Na]+, Hex8 DeoxyHex5 NeuAc4 N-Acetyl3 O-Methyl2 Sulfate2:[M-5H+6Na]+, Hex15 NeuAc2 N-Acetyl7 O-Methyl2 Sulfate3:[M-2H+3Na]+, Hex18 N-Acetyl9 O-Acetyl1 Sulfate3:[M-2H+3Na]+, Hex18 NeuAc1 N-Acetyl4 O-Methyl1 Sulfate2:[M-3H+4Na]+, Hex17 DeoxyHex2 N-Acetyl5 O-Acetyl2 O-Methyl1 Sulfate3:[M-H+2Na]+, Hex14 DeoxyHex4 NeuAc1 N-Acetyl4 O-Acetyl1 Sulfate3:[M-H+2Na]+, Hex11 DeoxyHex3 NeuAc3 N-Acetyl4 O-Acetyl1 O-Methyl2 Sulfate3:[M-2H+3Na]+, Hex8 DeoxyHex5 NeuAc4 N-Acetyl3 O-Methyl1 Sulfate3:[M-2H+3Na]+, Hex14 DeoxyHex3 NeuAc1 N-Acetyl6 O-Acetyl2 Sulfate3:[M-2H+3Na]+, Hex18 NeuAc1 N-Acetyl4 Sulfate3:[M+Na]+, Hex12 DeoxyHex1 NeuAc3 N-Acetyl8 O-Methyl1 Sulfate3:[M-3H+4Na]+, Hex16 DeoxyHex4 N-Acetyl3 O-Methyl2 Sulfate3:[M-2H+3Na]+
1937.369	0.233	Hex7 NeuAc1 N-Acetyl3 O-Acetyl1 Sulfate3:[M-3H+4Na]+
4703.639	0.223	Hex15 DeoxyHex3 NeuAc4 N-Acetyl11 O-Acetyl2 O-Methyl2:[M-3H+4Na]+, Hex12 DeoxyHex5 NeuAc5 N-Acetyl10 O-Acetyl1 O-Methyl1:[M-3H+4Na]+, Hex12 DeoxyHex5 NeuAc5 N-Acetyl10 O-Acetyl1 Sulfate1:[M+Na]+, Hex15 DeoxyHex3 NeuAc4 N-Acetyl11 O-Acetyl2 O-Methyl1 Sulfate1:[M+Na]+, Hex19 NeuAc3 N-Acetyl14 O-Acetyl1 O-Methyl1 Sulfate1:[M+Na]+, Hex16 DeoxyHex2 NeuAc4 N-Acetyl13 Sulfate1:[M+Na]+
4405.510	0.216	Hex12 DeoxyHex3 NeuAc5 N-Acetyl10 O-Methyl2:[M-4H+5Na]+, Hex21 N-Acetyl19 Sulfate2:[M-H+2Na]+, Hex14 DeoxyHex5 NeuAc3 N-Acetyl8 O-Acetyl2 O-Methyl1:[M-3H+4Na]+, Hex20 DeoxyHex2 N-Acetyl15 O-Acetyl1 O-Methyl1 Sulfate2:[M+Na]+, Hex17 DeoxyHex4 NeuAc1 N-Acetyl14 Sulfate2:[M+Na]+, Hex11 DeoxyHex5 NeuAc5 N-Acetyl6 O-Acetyl1 O-Methyl2 Sulfate1:[M+Na]+, Hex14 DeoxyHex5 NeuAc3 N-Acetyl8 O-Acetyl2 Sulfate1:[M+Na]+, Hex15 DeoxyHex1 NeuAc4 N-Acetyl11 O-Acetyl1 O-Methyl2 Sulfate1:[M-H+2Na]+, Hex12 DeoxyHex3 NeuAc5 N-Acetyl10 O-Methyl1 Sulfate1:[M-H+2Na]+, Hex18 DeoxyHex1 NeuAc2 N-Acetyl13 O-Acetyl2 Sulfate1:[M-H+2Na]+, Hex15 DeoxyHex2 NeuAc4 N-Acetyl9 O-Methyl2 Sulfate1:[M+Na]+, Hex21 NeuAc1 N-Acetyl12 O-Acetyl2 O-Methyl1 Sulfate1:[M+Na]+, Hex18 DeoxyHex2 NeuAc2 N-Acetyl11 O-Acetyl1 Sulfate1:[M+Na]+, Hex11 DeoxyHex6 NeuAc4 N-Acetyl9 O-Acetyl2:[M-4H+5Na]+, Hex18 DeoxyHex1 NeuAc3 N-Acetyl8 O-Acetyl1 O-Methyl1:[M-2H+3Na]+, Hex15 DeoxyHex3 NeuAc4 N-Acetyl7:[M-2H+3Na]+
1175.158	0.214	Hex5 O-Acetyl2 Sulfate3:[M+Na]+
2105.408	0.210	Hex7 DeoxyHex1 NeuAc1 N-Acetyl3 O-Acetyl1 Sulfate3:[M-4H+5Na]+

Supplementary Figures

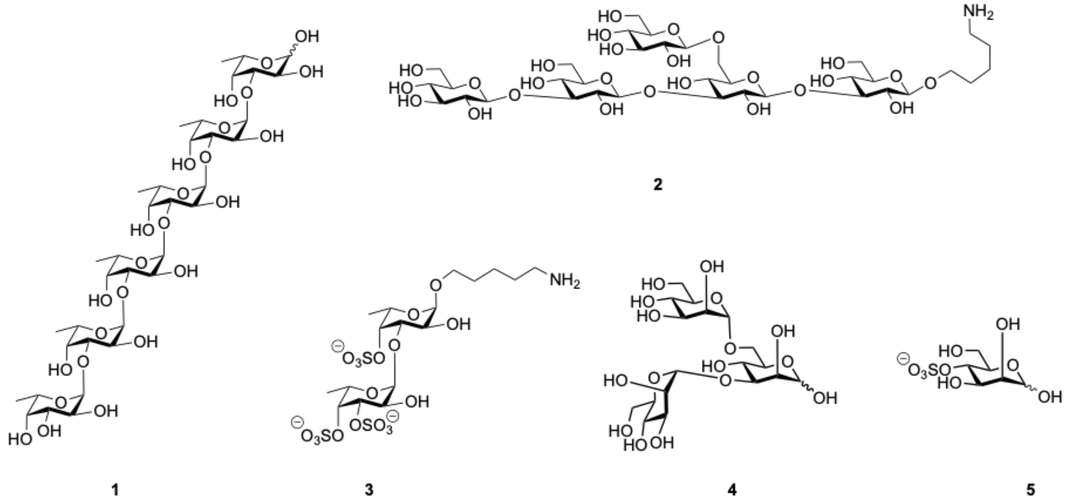

Figure S1 | Synthetic compounds used in this study.

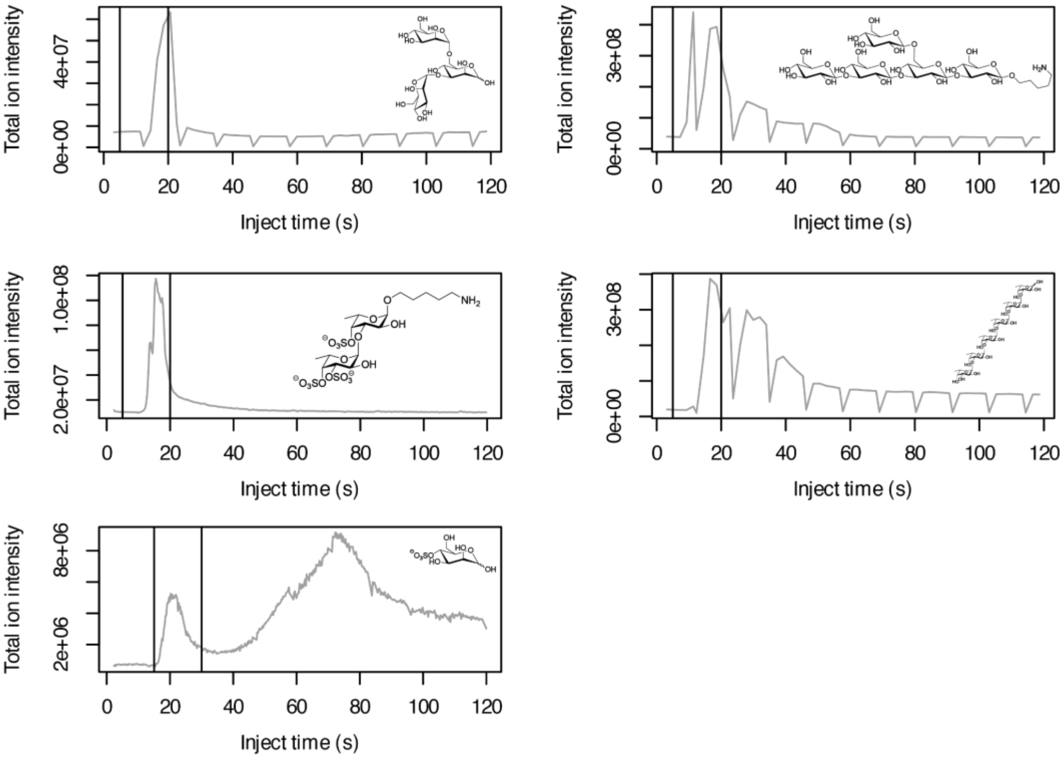

Figure S2 | TICs of QTOF data from synthetic oligosaccharides. Black vertical lines indicate the times between which the spectra were averaged. Synthetic structures are shown on each panel.

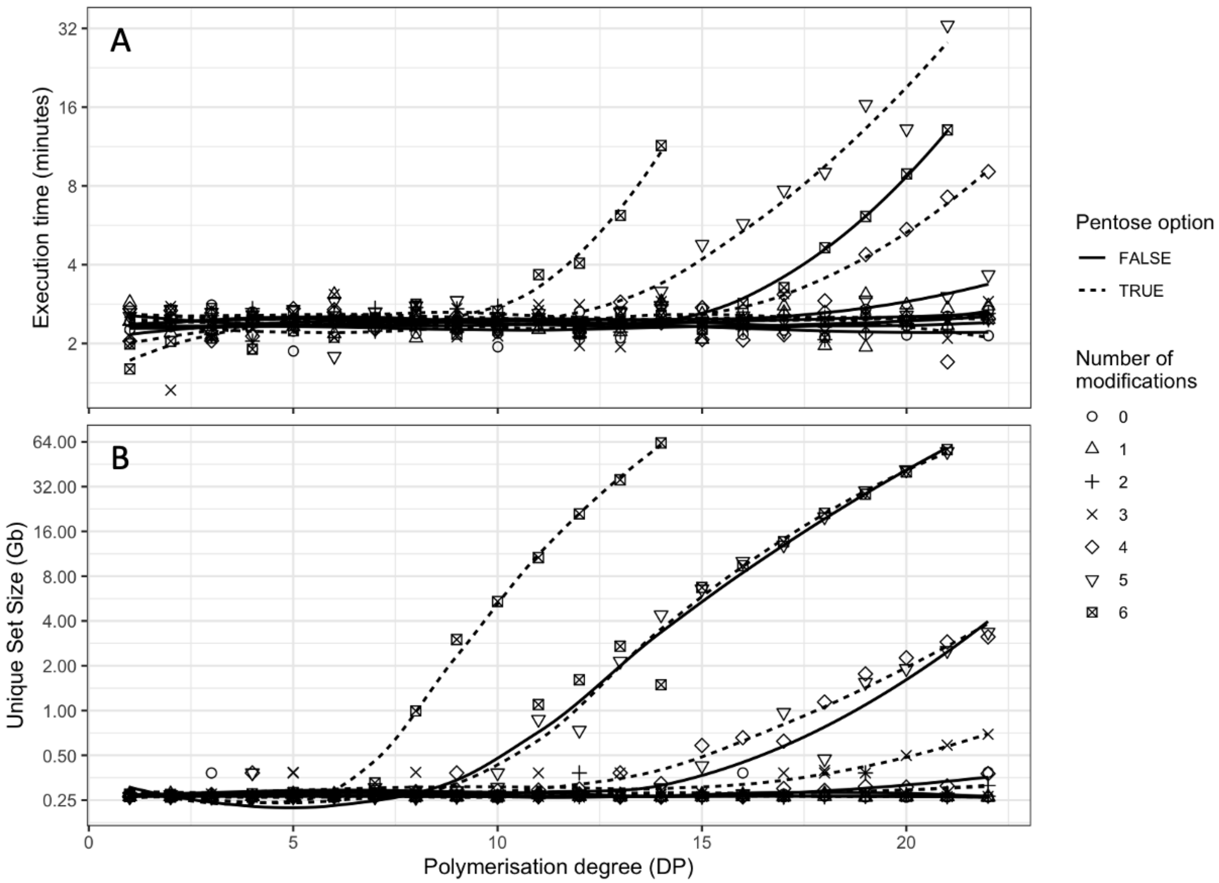

Figure S3 | Benchmarking of 'glycoPredict' function. Unique Set Size in gigabytes (A) and execution time in minutes (B) for chains with polymerisation degrees (DP) from 1 to 22, and number of modifications from 1 to 6, with and without pentoses. Only the maximum value among 10 repetitions of the same process is displayed. The y-axis has a log2 scale.

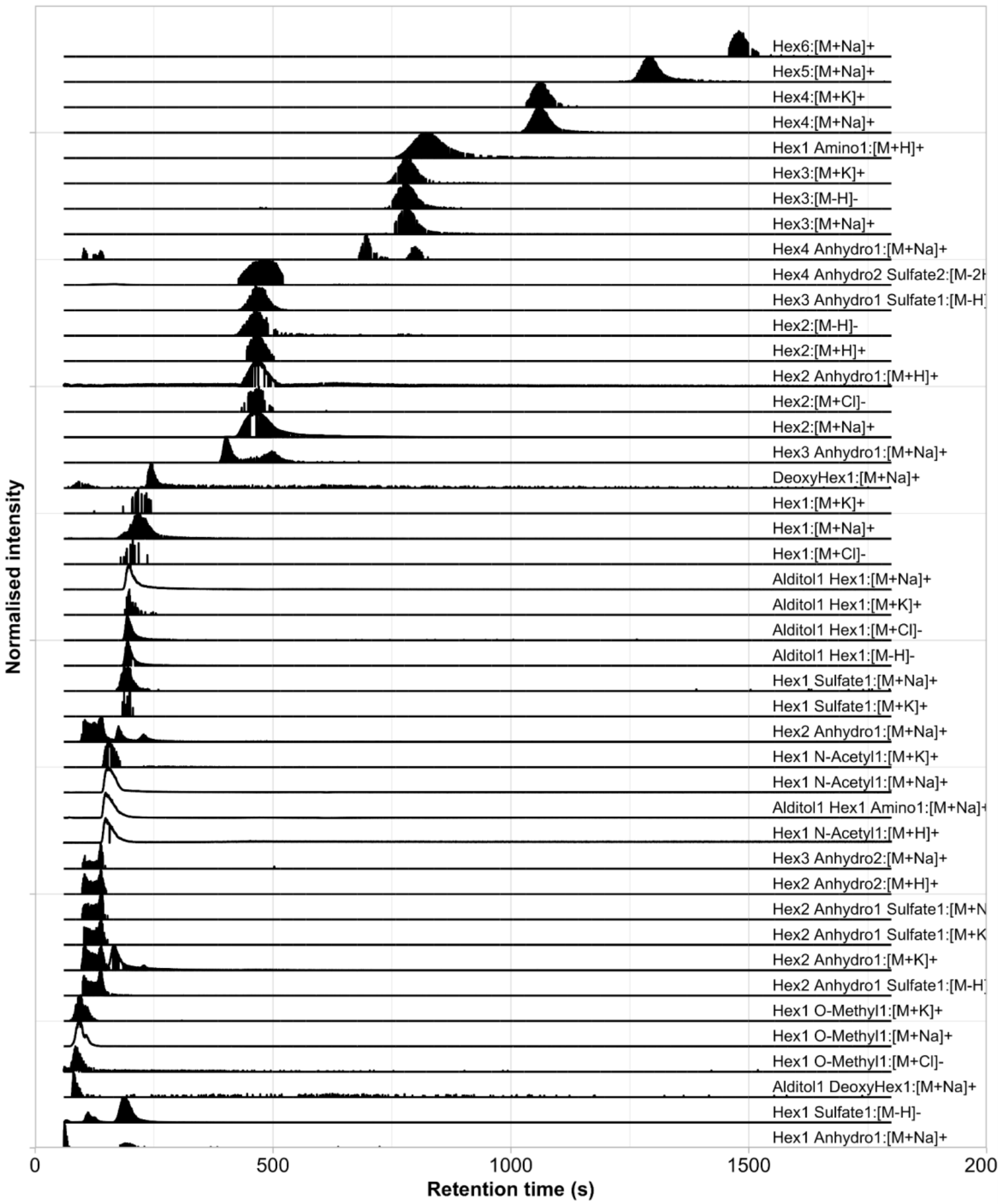

Figure S4 | Extracted ion chromatograms from LC-MS analysis of a mixture of commercial standards.

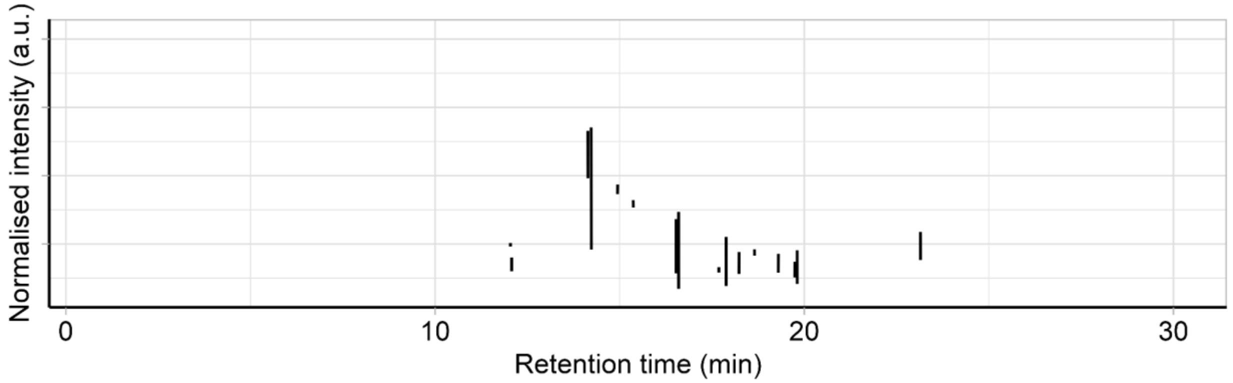

Figure S5 | No galacturonic acid [M-H]$^-$ peaks were picked in LC-MS data of commercial standards. Extracted ion chromatogram from commercial standard mixture in negative polarity (*m/z* 193.0354 +- 2 ppm).

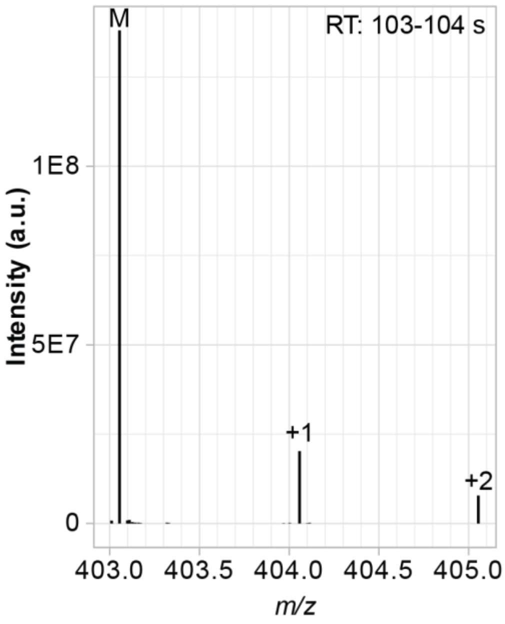

Figure S6 | Isotopes of negative mode 403.0546 *m/z* feature at ~1.7 min helped to resolve ambiguous annotation. The feature was annotated as either Hex4 Anhydro1 Sulfate2 [M-2H]-2 (403.0552 *m/z*) or Hex2 Anhydro1 Sulfate1 [M-H]- (403.0552 *m/z*). M+1 and M+2 ions support annotation of a singly charged ion.

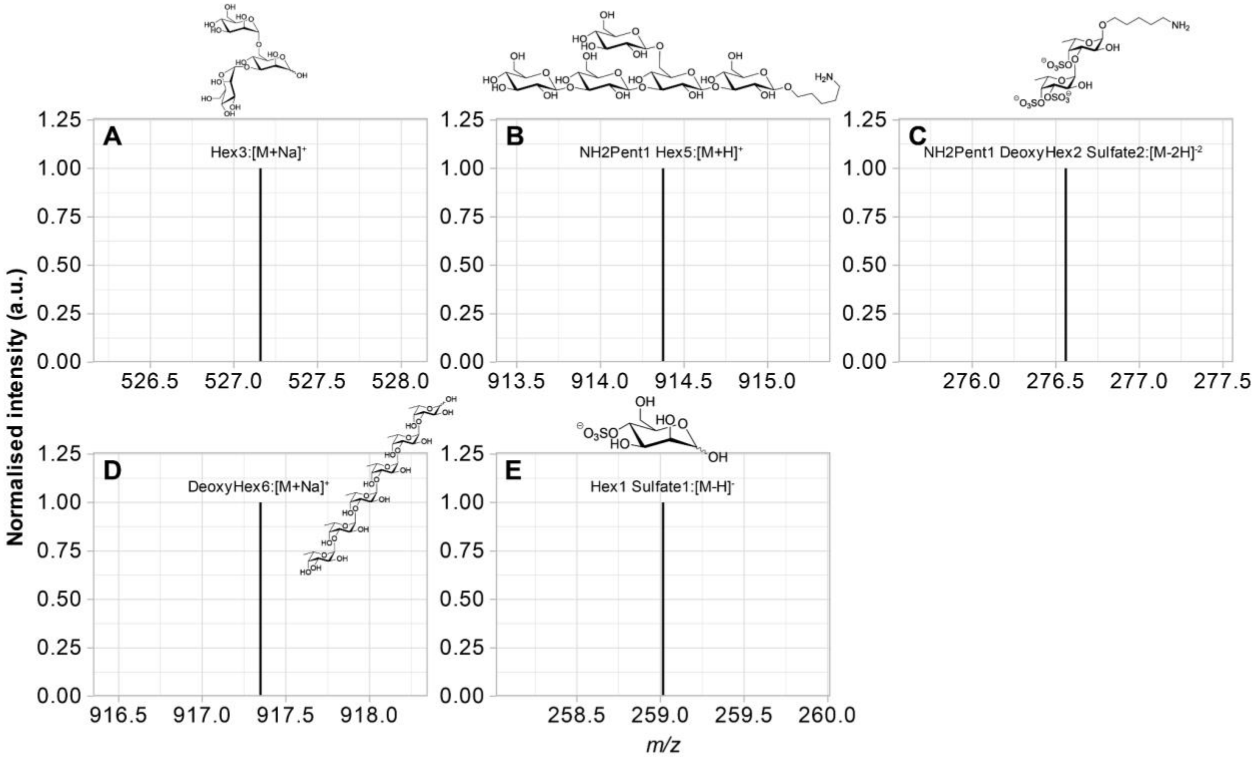

Figure S7 | Synthetic standards annotated in QTOF data. Averaged and normalised spectra from synthetic standards directly infused into a QTOF mass spectrometer. Only peaks with normalised intensities >0.8 are shown. Annotations correspond to the standards synthesised. Synthesised structures are shown above spectra. Observed *m/z* values and corresponding annotations for **A-E** are as follows: **A** = 527.16, Hex3 $[M+Na]^+$; **B** = 914.37, NH2Pent1 Hex5 $[M+H]^+$; **C** = 276.56, NH2Pent1 DeoxyHex2 Sulfate2 $[M-2H]^{-2}$; **D** = 917.35, DeoxyHex6 $[M+Na]^+$; **E** = 259.02, Hex1 Sulfate1 $[M-H]^-$.

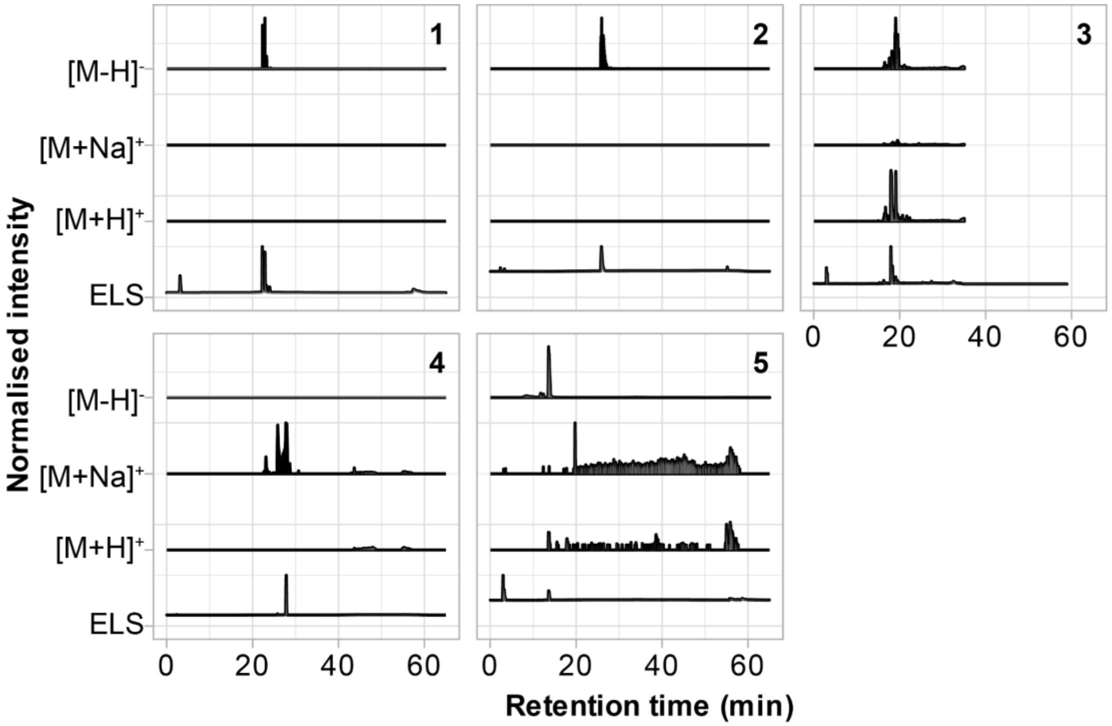

Figure S8 | HPLC-ELS-MS traces for synthetic compounds. Ion chromatograms were extracted for *m/z* values of adducts of the 5 compounds ± 0.1 Da. Intensities for extracted ion chromatograms and ELS data were normalised with respect to the maximum intensity. A porous graphitised carbon (PGC) column was used for chromatographic separation.

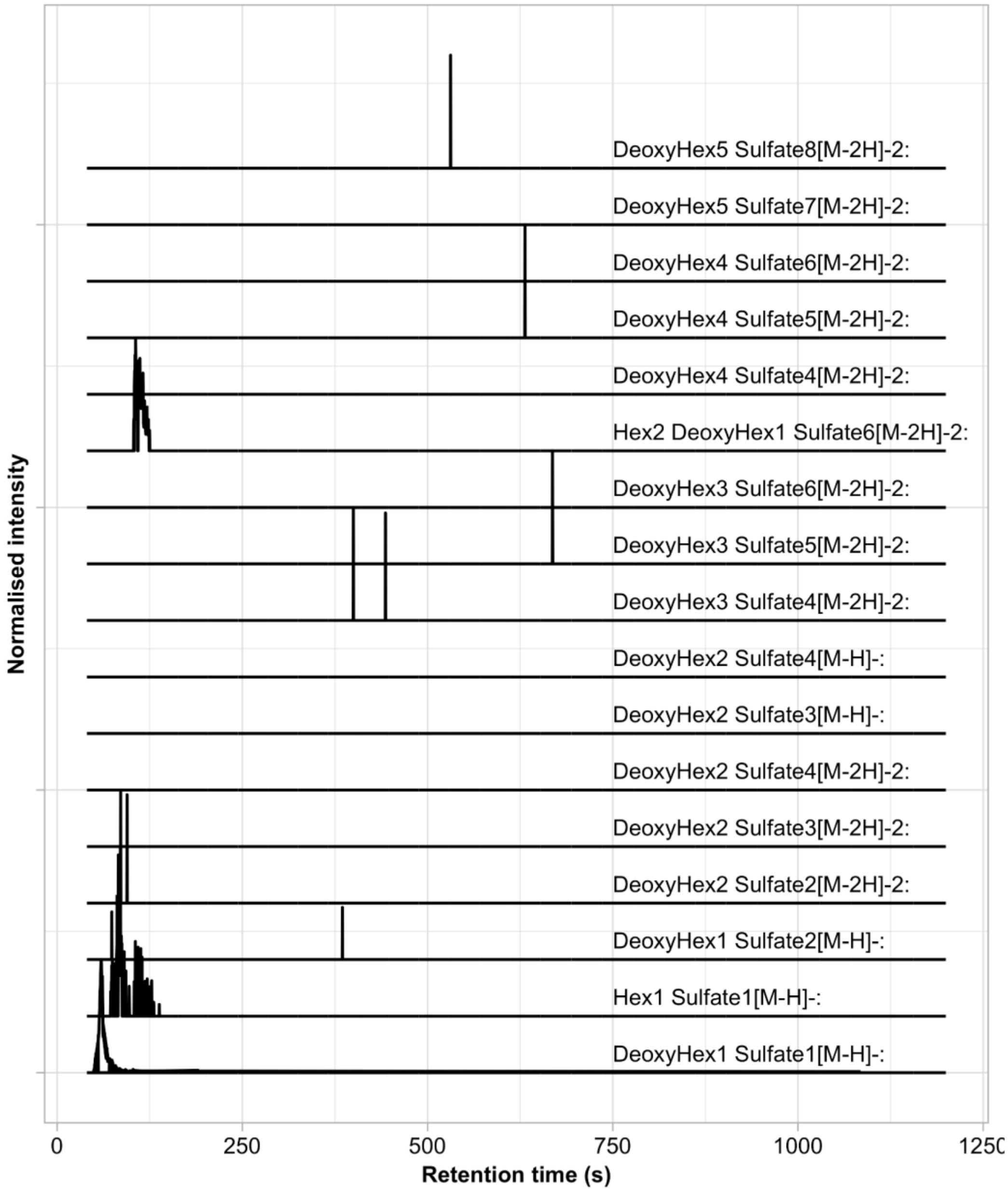

Figure S9 | Extracted ion chromatograms from fucoidan digest negative control. Extracted ion chromatograms for oligosaccharide ions that were annotated in LC-MS data of the *M. pyrifera* fucoidan digest by GlycoAnnotateR. Ion intensities were normalised for each chromatogram with respect to the maximum ion intensity. Chromatograms were extracted for the *m/z* ranges of the chromatographic peaks detected by the CentWave algorithm. Hex2 DeoxyHex1 Sulfate6 $[M\text{-}H]^-$, Hex1 Sulfate1 $[M\text{-}H]^-$ and one DeoxyHex1 Sulfate1 $[M\text{-}H]^-$ peaks were excluded from the final peak count as they likely represent mis-annotated noise and contaminant ions.

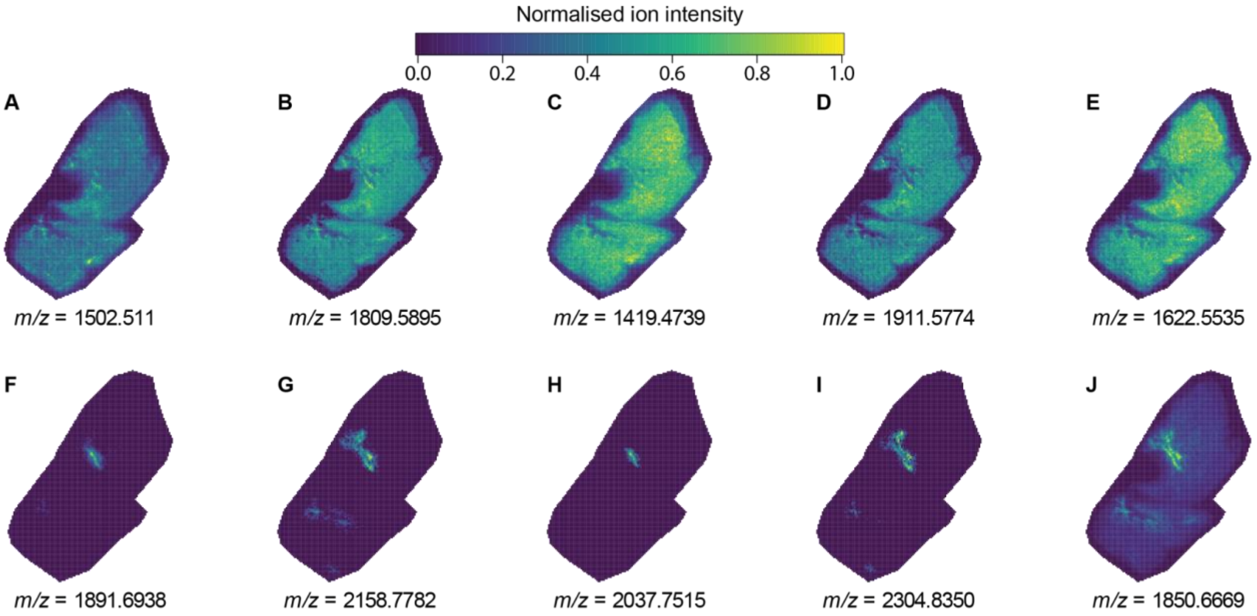

Figure S10 | Spatial distribution patterns of annotated glycans previously obtained with METASPACE and NGlycDB were reproduced with Cardinal and GlycoAnnotateR. Examples from class 3 (A-E) and class 1 (F-J) after segmentation analysis, representing uniform distribution across the section and co-localisation with the main aerial vessel respectively. Ion images were extracted with Cardinal for the *m/z* values indicated in each panel (+/- 0.005). Colours indicate normalised ion intensities. Annotations assigned to ions are as follows: **A** = Hex8 N-Acetyl3 O-Acetyl1 $[M+Na]^+$; **B** = Hex9 DeoxyHex1 N-Acetyl4 $[M+Na]^+$; **C** = Hex8 N-Acetyl2 [M+Na]+; **D** = Hex9 DeoxyHex1 N-Acetyl4 Sulfate1 $[M-H+2Na]^+$; **E** = Hex9 N-Acetyl3 $[M+Na]^+$; **F** = Hex9 DeoxyHex1 N-Acetyl6 $[M+Na]^+$; **G** = Hex10 DeoxyHex2 N-Acetyl5 $[M+Na]^+$; **H** = Hex9 DeoxyHex2 N-Acetyl6 $[M+Na]^+$; **I** = Hex10 DeoxyHex3 N-Acetyl5 $[M+Na]^+$; **J** = Hex9 DeoxyHex1 N-Acetyl5 $[M+Na]^+$.

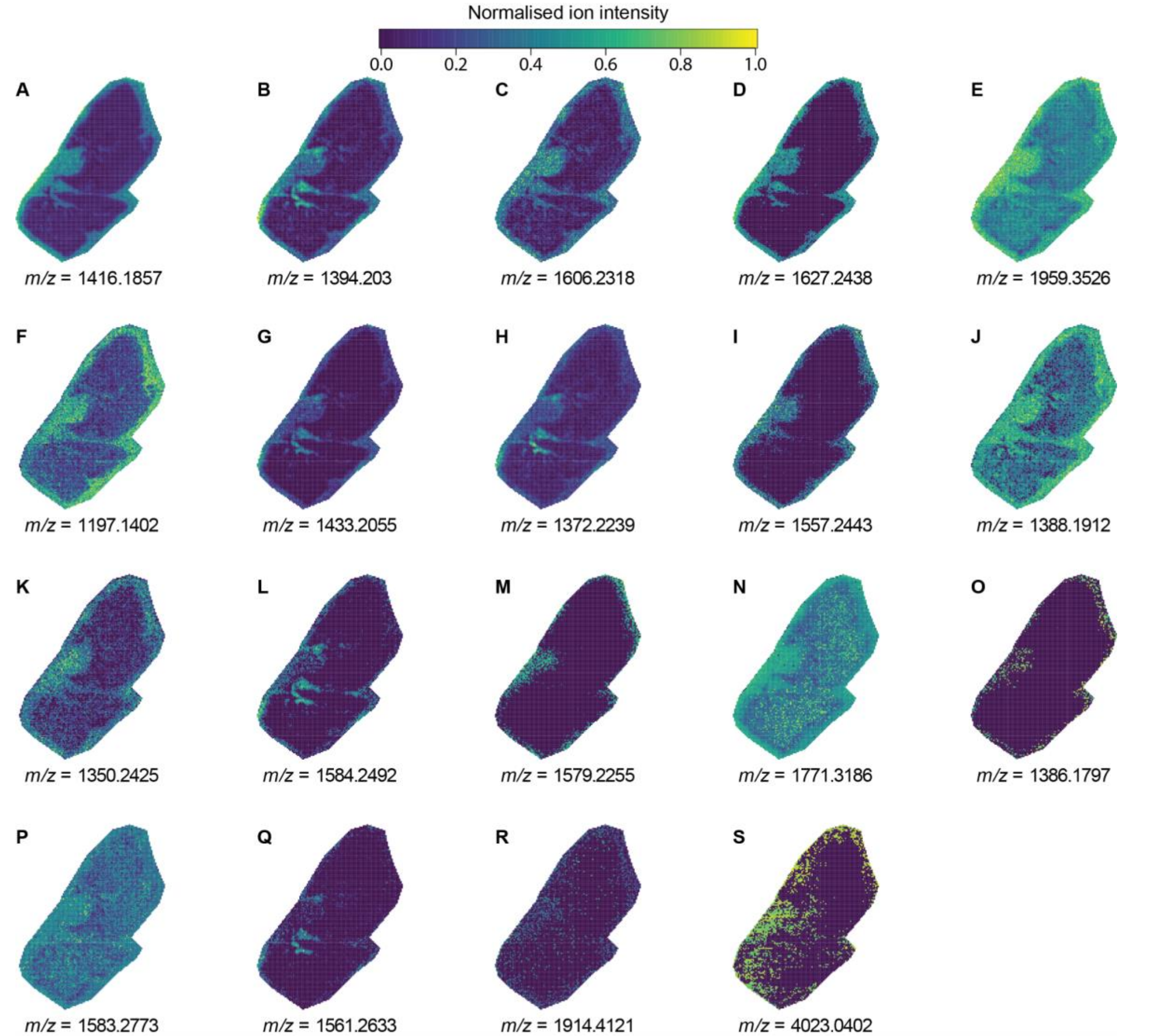

Figure S11 | Spatial distribution pattern of ions annotated as tri-sulfated glycans by GlycoAnnotateR. Top 19 ranked peaks in a class of ions from segmentation analysis that are localised to the outer edge of the tissue section after. Ion images were extracted with Cardinal for the *m/z* values indicated in each panel (+/- 0.005). Colours indicate normalised ion intensities. Annotations assigned to ions are as follows: **A** = Hex6 N-Acetyl1 O-Acetyl1 O-Methyl1 Sulfate3: [M-3H+4Na]$^+$; **B** = Hex6 N-Acetyl1 O-Acetyl1 O-Methyl1 Sulfate3: [M-2H+3Na]$^+$; **C** = Hex7 N-Acetyl1 O-Acetyl2 Sulfate3: [M-3H+4Na]$^+$; **D** = Hex7 DeoxyHex1 Sulfate3: [M-3H+4Na]$^+$; **E** = Hex7 NeuAc1 N-Acetyl3 O-Acetyl1 Sulfate3: [M-4H+5Na]$^+$; **F** = Hex5 O-Acetyl2 Sulfate3: [M-H+2Na]$^+$; **G** = Hex4 DeoxyHex3 Sulfate3: [M-3H+4Na]$^+$; **H** = Hex6 N-Acetyl1 O-Acetyl1 O-Methyl1 Sulfate3: [M-H+2Na]$^+$; **I** = Hex7 O-Acetyl2 O-Methyl1 Sulfate3: [M-2H+3Na]$^+$; **J** = Hex6 N-Acetyl1 O-Methyl2 Sulfate3: [M-3H+4Na]$^+$; **K** = Hex6 N-Acetyl1 O-Acetyl1 O-Methyl1 Sulfate3: [M+Na]$^+$; **L** = Hex7 N-Acetyl1 O-Acetyl2 Sulfate3: [M-2H+3Na]$^+$; **M** = Hex7 O-Acetyl2 O-Methyl1 Sulfate3: [M-3H+4Na]$^+$; **N** = Hex6 DeoxyHex3 O-Methyl1 Sulfate3: [M-3H+4Na]$^+$; **O** = Hex5 DeoxyHex1 N-Acetyl1 O-Acetyl1 Sulfate3: [M-3H+4Na]$^+$; **P** = Hex7 DeoxyHex1 Sulfate3: [M-H+2Na]$^+$; **Q** = Hex6 DeoxyHex1 N-Acetyl2 O-Methyl1 Sulfate3: [M-3H+4Na]$^+$; **R** = Hex9 N-Acetyl1 O-Acetyl2 O-Methyl2 Sulfate3: [M-H+2Na]$^+$, Hex7 NeuAc1 N-Acetyl4 Sulfate3: [M-2H+3Na]$^+$; **S** = Hex14 NeuAc4 N-Acetyl3 O-Acetyl2 O-Methyl1 Sulfate3: [M-4H+5Na]$^+$, Hex10 DeoxyHex4 NeuAc4 N-Acetyl5 O-Methyl1 Sulfate3: [M-7H+8Na]$^+$, Hex13 DeoxyHex3 NeuAc3 N-Acetyl4 O-Methyl2 Sulfate3: [M-6H+7Na]$^+$. R and S were assigned multiple annotations that could not be resolved.

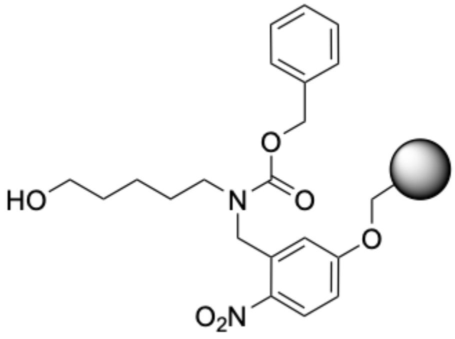

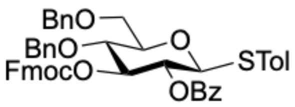

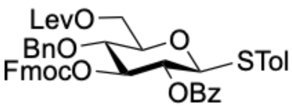

L1 **6** **7**

Figure S12 | Solid-phase resin and thioglycoside building blocks used in this study.

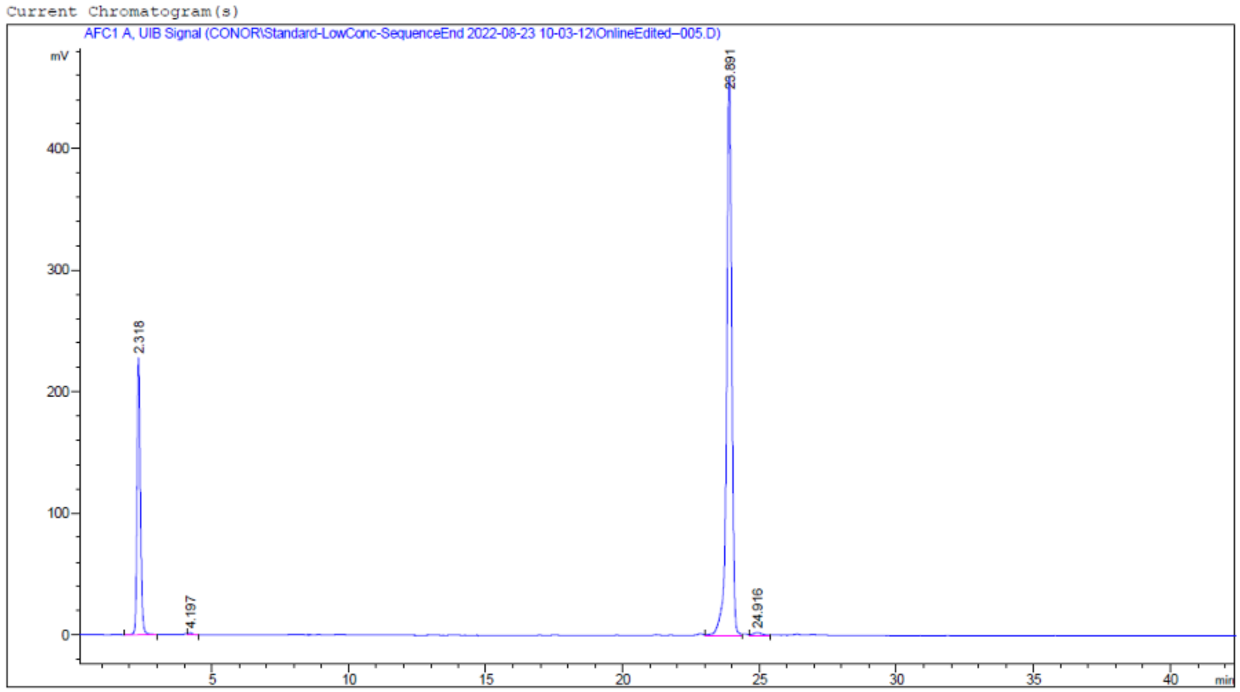

Figure S13 | Crude HPLC of automated synthesis of glucan. ELSD Trace.

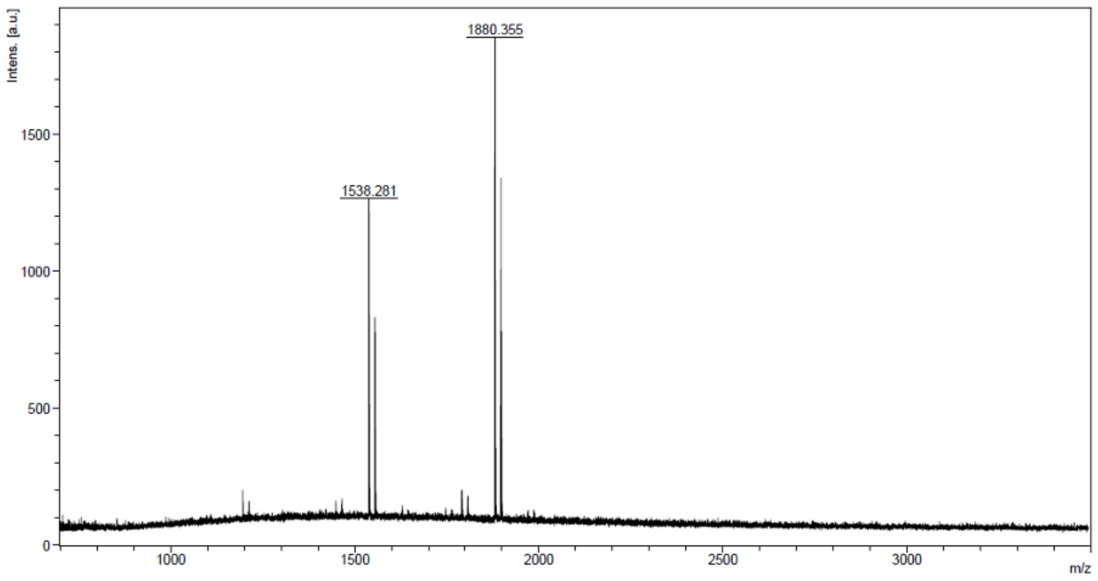

Figure S14 | MALDI-TOF of microcleavage sample of 2 following on-resin methanolysis.

Supplementary Information

1. Extended methods

1.1) Chemical rules

As GlycoAnnotateR is based on compositions only, all limits are enforced based only on the average number of modifications per composition. For example, a monomer cannot have both a carboxylic acid and an anhydro-bridge. This limit is enforced in practice by removing all compositions where the number of carboxylic acid and anhydro-bridge modifications together is equal to or less than the DP. So, a DP2 can have 1 carboxylic acid and 1 anhydro-bridge. However, the nature of the annotation as a composition intrinsically means that the annotation is not describing a molecule with a carboxylic acid-modified monomer linked to an anhydro-bridge-modified monomer (although it is assumed). Alditol, unsaturated, dehydrated, and amino-pentyl linker modifications are allowed only once per composition.

General assumptions made for this tool: To reduce the vast number of theoretical compositions for modified hexose and/or pentose-based oligosaccharides, we have defined a set of chemical rules to filter the calculated compositions to a set of 'meaningful' putative compositions, Table S1. The chemical rules defined in the following section were derived based on two main approaches. The first approach taken was a first principle chemical approach representing assumptions made regarding the likelihood of modifications occurring together on a given monomer. The second approach is based on observations from reviewing the literature for natural products of hexose and/or pentose-based oligosaccharides. These rules, therefore, only account for naturally occurring hexose and/or pentose-based oligosaccharides and cannot be applied to synthetically derived oligosaccharides, by design. It should be noted that the amino-pentose linker represents the only exception to these rules and is the only chemically synthesised modification used in this paper.

General Condition: The chemical rules applied in this tool are based on the assumption that the modifications do not cleave glycosidic linkages and that the glycan chain is not altered.

1.1.1) Modifications per monosaccharide and general assumptions

The first broad rule applied to the set of theoretically calculated confirmations is that the same chemical modification cannot occur on a single monomer unit more than once, **Table S1**. When considering the general condition that no glycosidic linkages can be broken or the glycan chain is

not altered, these rules become apparent. For example, from a first principles approach, in the case of C6- attached functional groups such as anhydro-bridges, phosphate, and carboxyl moieties, lead to mutual exclusion of the other modification due to the preclusion of available attachment sites that will not break the glycosidic linkage. Other examples of this rule are derived from observations in the literature, such as amino or N-acetyl functional groups, that almost exclusively occur at the C2 position in glycans[1]. The one notable exception to this first rule is for doubly sulfated bis-sulfated monomer units, as they have often been observed in our study of fucose-containing sulfated polysaccharides (FCSP)[2]. It should be noted that “pentose” and “sialic acid” are monomer units, however, they are included in **Table S1** as not all modifying functional groups are allowed on these monomer units. Although deoxy-pentose sugars are an essential building block in DNA, they are rarely found in natural oligosaccharides, and are therefore not included as a possible modification by this tool. The arguments and justifications for the remaining rules outlined in **Table S1** are briefly described in the following section below.

1.1.2) Rules for alditols

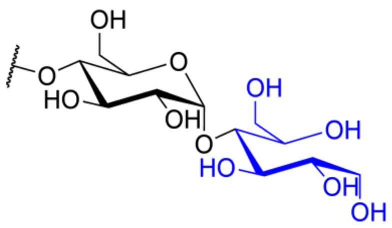

Figure S15 | Alditol derivatization of a generic dimer of glucose, with the reducing end monomer converted to an alditol (blue).

Alditols are acyclic polyalcohols. They are typically produced by reducing an aldehyde or a ketone group at the reducing end of glycans[3]. In the context of this tool, the alditol modification is only allowed once per oligosaccharide composition as only one reducing end exists. Groups that are considered as theoretically possible pairings with alditols on a single monomer are, sulphate, phosphate, carboxylic acid, O-methyl, O-acetyl, N-acetyl, and amino groups. Groups that are unlikely to be in combination with an alditol are reactive modifications, such as anhydro-bridges, carboxylic acids and unsaturation or dehydration. It is assumed that these reactive modifications would most likely be altered before forming an alditol.

1.1.3) Rules for dehydration

The modification “dehydration” refers to the removal of water, specifically the loss of a hydroxyl group and hydrogen resulting in the formation of an alkene, this directly equates to the removal

of the exact mass of water from a given monomer unit in the overall oligosaccharide composition. In this context, the mechanism of dehydration follows that of elimination. Whether the elimination is of type E1, E2 or E1cB depends on the predominant conditions of a given monomer unit, something the tool can only estimate. In this tool we consider a single hexose or pentose monomer as well as its inclusion as part of a larger oligosaccharide composition, and assume that dehydration occurs primarily through the E2 mechanism over that of the E1 and E1cB. Briefly, E2 is a concerted mechanism where the leaving group and the hydrogen that is removed are always in an anti-periplanar conformation, meaning in the context of this tool the hydroxyl group must be in an axial position[4]. In the E1 and E1cB mechanisms, intermediate carbocation or carbanion are formed respectively. Because of the carbocation, the planarity of the ring in E1 is increased, contributing to the strain making it less favoured for elimination. In E1cB the formation of the carbanion makes the intermediate highly unstable[4]. The equatorial or axial position of the hydroxyl groups is given by the type of monosaccharide; the abundance of this modification is already reduced by the probability of the suitable anti-periplanar conformation for the E2 case. Therefore, the probability of this modification depends on the glycan type. Due to the individual aspects of each modification, we have had to evaluate the combination of dehydration with other modifications case by case to estimate their likeliness to occur together, while also not breaking the general condition of the tool. These modifications are unsaturation, amine/amide, and anhydro-bridge.

Case 1: Dehydration + unsaturation

A dehydrated and unsaturated monomer unit would have an increased ring strain that likely leads to the breaking of the glycan chain going against the general condition of these rules. A combination of those two modifications is unlikely for that reason[5].

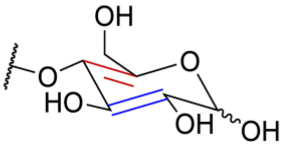

Figure S16 | Theoretical monomer building block with a dehydration (blue) and an unsaturation (red).

Case 2: Dehydration + amine/amide

The combination of dehydration with a N-acetylation or an amine seems to be possible, nevertheless, this combination did not appear in the literature of natural products to the best of our knowledge, **Fig. S17**[1]. One explanation for this is that in one of two possible conformations in

a hexose, an enamine can be formed with increased reactivity towards electrophiles, making it unstable. Consequently, the chance for this combination of motifs to occur is likely diminished.

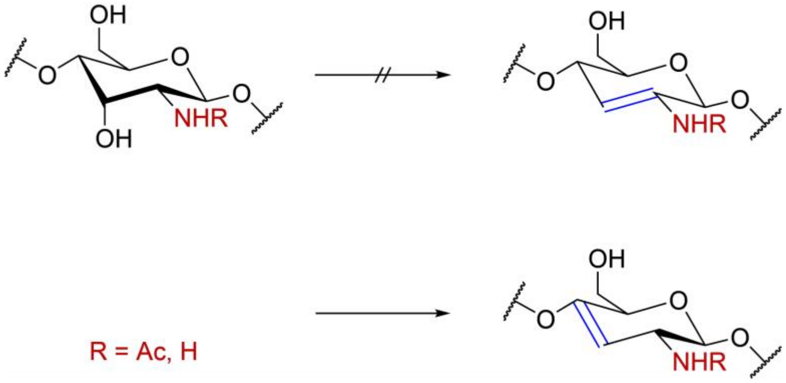

Figure S17 | Dehydration (blue) on a N-acetylated or amino (red) sugar.

Case 3: Dehydration + anhydro-bridge

A dehydrated monomer in combination with an anhydro-bridge modification is unlikely to appear, due to three major reasons. The first is the availability of hydroxyl groups given a single hexose monomer unit. Of the five available hydroxyl groups, two are used in the glycosidic linkages, and another two are required for the anhydro-bridge, one of them must be the C6 hydroxyl group. Only one free hydroxyl group is left on either the C2, C3 or C4. Additionally, for the dehydration *via* E2 mechanism the hydroxyl groups must be in axial position[4]. Secondly, the formation of an anhydro-bridge favours low sterical hindrance at the attacked carbon. Lastly, Bredt's rule states that it is highly unfavourable or impossible for double bonds to form at bridgehead carbons, this reduces the amount of possible double bonds in the bicyclic system following the formation of the anhydro-bridge[6]. It should also be noted that anti-bredt systems were not considered when designing this tool[7]. These cases are illustrated in **Fig. S17** and **S18** and describe two scenarios A and B, depending on which modification appeared first at the monomer. Case A shows a monomer with a dehydration modification before an anhydro-bridge is formed whereas Case B shows the inverse. Although some feasible pathways are suggested in **Fig. S18** and **S19**, when considering steric hindrance and the chance of getting any particular combination, it was concluded that having an anhydro-bridge on a dehydrated monomer and vice versa, was not feasible and therefore left out as a possible combination in the tool.

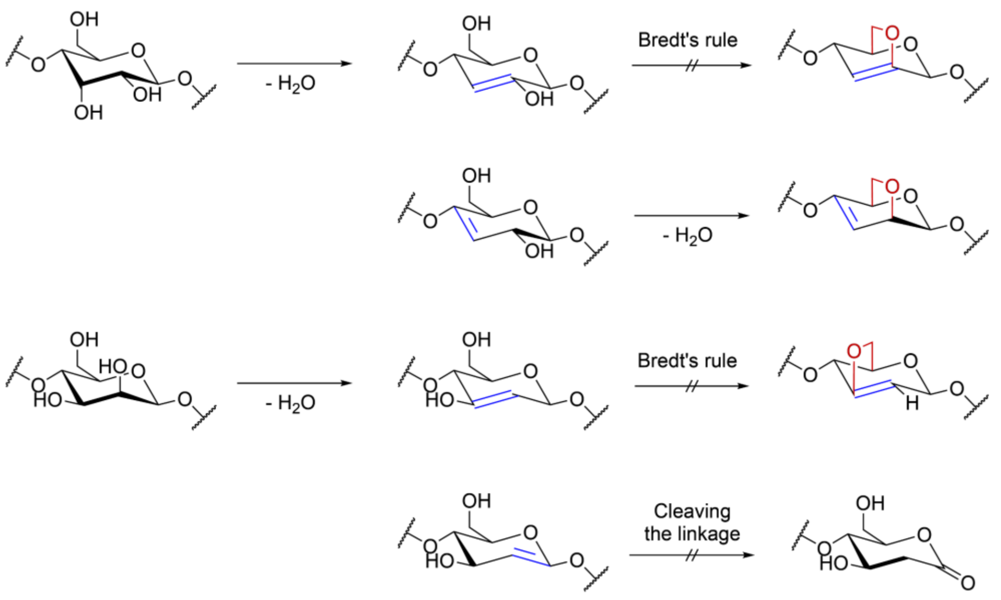

Figure S18 | Case A: First dehydration (blue) a) C3 -> C2, b) C3 -> C4, c) C2 -> C3, d) C2 -> C1, then anhydro-bridging (red).

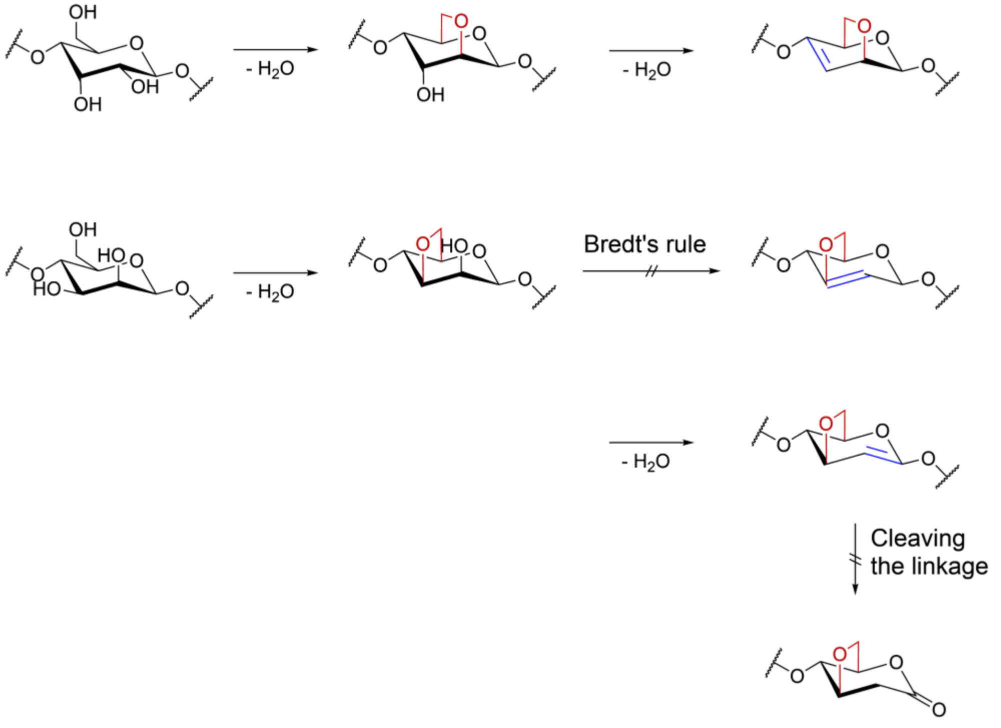

Figure S19 | Case B: First anhydro-bridge (red) a) C6 -> C2, b) C6 -> C2, then dehydration (blue).

1.1.4) Rules for deoxy modification

The deoxy modification refers to the removal of a hydroxyl group and replacement by a hydrogen atom in a given monomer unit. This has the effect of decreasing the steric hindrance of the monomer and in principle allows it to occur with most other modifications. Following an exhaustive literature search no examples could be found where a deoxy was found in combination with carboxylic acid or anhydro-bridge modifications. This was also the case for deoxy-pentose structures apart from DNA.

1.1.5) Rules for amino groups and N-acetylation

Amino/Amide groups occur preferably in positions C1, C2, and C3[8,9]. Naturally they are seldom found in combination with other modifications as amino groups are less stable than amide groups. It is unlikely to find free amine groups when an acetylation to an amide group is possible. The combination of an amine group and an amide modification was not included. In a non-exhausting literature search, N-Acetylation in combination with an unsaturated or dehydrated bond in the monomer was not found. Reasons for that were suggested in section 1.1.3 Case 2.

1.1.6) Rules for anhydro bridge

The anhydro-bridging is possible only from the C6 carbon to either C2, C3 or C1, and therefore only once per monomer. Connecting C6 with C1 would cleave the glycosidic linkage. A combination with a carboxylic acid or phosphate modification is not possible since all of them are formed at the C6 position.

Anhydro-bridge + unsaturation:

The combination of a bridged monomer, including an unsaturated bond, and other anti-bredt systems were not considered in this tool[7]. Considering Bredt's rule only two conformations can be formed. Connecting C6 and C3 leaves only the bond from C1 to C2 open for an unsaturation modification, which would also cleave the glycan chain. The only allowed possibility in this tool is an anhydro-bridge to the C2 carbon and an unsaturated bond between C4 and C5. However, the ring strain would likely be increased, destabilising the glycan. A combination of these two modifications was assessed to be unlikely. **Fig. S20** shows the only chemical possible combination in a monomer.

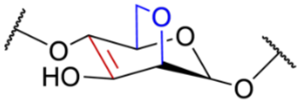

Figure S20 | Anhydro-bridge from C6 to C2 (blue) with an unsaturated bond between C4 and C5 (red).

1.1.7) Rules for O-Acetylation

O-Acetylation can occur on all free hydroxyl groups. If the C6 carbon has been acetylated it is not possible to form an anhydro-bridge anymore. Likewise, an anhydro-bridge is not stable under acetylating conditions[10]. **Fig. S21** shows the incompatibility of an anhydro-bridge with an O-acetylation within a monomer.

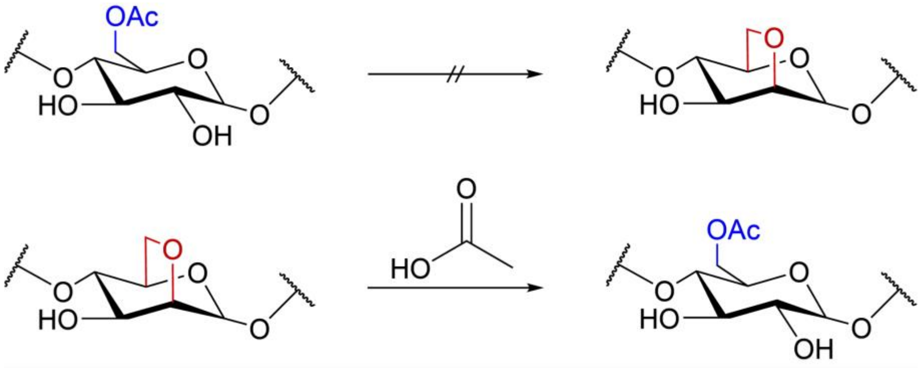

Figure S21 | Incompatibility of an anhydro-bridge (red) with an O-acetylation (blue) modification within a monomer.

1.2) Benchmarking

Benchmarking of the ‘glycoPredict’ function of GlycoAnnotateR was done using Snakemake v7.17.1[11]. Each process was benchmarked 10 times. The function ‘glycoPredict’ could finish in less than 10 minutes using less than 4 Gb of RAM for a DP value of 22, modification number of 4, and pentose option activated (**Fig. S3**), meaning it will easily run on a standard computer. Prediction of more complex chains, especially with a higher modification number and taking pentoses into account, scales quickly in resources but remains in manageable ranges (less than 64 Gb and 10-30 minutes of execution time). Changing the format of the output (‘long’ or ‘wide’) and the ion type (‘ESI’ or ‘MALDI’) had no effect on the needed memory nor on the execution time.

1.3) LC-MS/MS of commercial standards

The standard mixture was diluted 1:9 (v/v) with acetonitrile (LC-MS grade) for a final concentration of 2 µM per compound. Standards were analysed with a Thermo Fisher Vanquish Horizon UHPLC coupled to a Thermo Fisher Q-Exactive Plus MS controlled with Chromeleon software (version 7.2.10; Thermo Fisher Scientific, Waltham, USA). Samples were injected (5 µL) onto a Accucore-150-amide HILIC column (Thermo Scientific, USA) with the dimensions 100 mm long, 2.1 mm diameter and a particle size of 2.6 µm held at 60°C. Solvents A and B consisted of 10 mM ammonium formate at pH 5 and UHPLC-grade acetonitrile, respectively. The flow rate was 0.4 mL min^{-1}. Chromatographic conditions were as follows: 1 min 90% B; from 90% to 60% B over 40 min; from 60% to 40% B over 4 min; from 40% to 90% B over 3 min; 16 min 90% B.

Mono- and oligosaccharides were detected in both positive and negative mode (separate runs). Positive mode MS settings were: spray voltage of 3.9 kV; sheath, auxiliary and sweep gas flow rates, 50, 13 and 3 respectively; capillary temperature, 300°C; S-lens RF level, 60; auxiliary gas heater temperature, 320°C; resolution, 70,000; scan range, 175 to 1,400 *m/z*. Negative mode MS settings were: spray voltage of 3.9 kV; sheath, auxiliary and sweep gas flow rates, 50, 13 and 3 respectively; capillary temperature, 300°C; S-lens RF level, 60; auxiliary gas heater temperature, 320°C; resolution, 70,000; scan range, 175 to 1,400 *m/z*. MS2 data were collected in DDA mode with an inclusion list (**Table S3**). The MS2 resolution was 35,000.

Data were pre-processed with XCMS[12] and CAMERA[13] in R. Negative and positive polarity data were pre-processed separately but in parallel. Peak picking was performed with the CentWave algorithm at a ppm of 3 and peak width of 10-120[14]. Neighbouring peaks were merged with retention time expanded by 3 s and a minimum proportion of 0.5. Peaks were then grouped into features (one group with all samples) with a minimum fraction of 0.5 and bandwidth of 5. Missing peaks were filled using default parameters only. Data objects were converted to 'xcmsSet' objects, which were then used to construct 'xsAnnotate' objects with CAMERA[13]. Peaks were grouped by retention time with a percentage full-width half maximum of 0.75. Isotopes were then annotated at 3 ppm. Finally, a peak table was generated from the 'xsAnnotate' object and features were filtered to retain only those with integrated intensity values of at least 5E5 in one sample.

The filtered feature table was annotated with the glycoAnnotate function from GlycoAnnotateR. Non-default parameters for positive polarity: DP 1-6; polarity positive: alditol, deoxy, amino, N-

acetyl, sulfate, O-methyl, carboxylic acid and anhydro-bridge modifications; H, Na and K adducts; error 1.5 ppm. Non-default parameters for negative polarity: DP 1-6; polarity negative: alditol, deoxy, amino, N-acetyl, sulfate, O-methyl, carboxylic acid and anhydro-bridge modifications; H and Cl adducts; error 2 ppm. Isotopes of negative polarity 403.0546 *m/z* feature at ~1.7 min helped to resolve ambiguous annotations. The feature was annotated as either Hex4 Anhydro1 Sulfate2 $[M-2H]^{-2}$ or Hex2 Anhydro1 Sulfate1 $[M-H]^{-}$, both of which have the same theoretical *m/z* value to four decimal places (403.0552 *m/z*). M+1 and M+2 ions with mass increases of ~1 Da support annotation of a singly charged ion (**Fig. S6**).

MS2 spectra associated with annotated features were extracted from negative polarity data with glycoMS2Extract. Spectra were then processed using functions from MSnBase v2.28.1[15]. Spectra were first cleaned to remove zero intensity masses, then averaged across spectra (method = meanMzInts, mzd = 0.001) and normalised with respect to the most intense ion. After converting the combined and averaged spectra to data.frame format, precursors were annotated using the same glycoPredictParam settings as annotation of the negative polarity MS1 peaks, except at 3 ppm. Fragment ions were then annotated using the glycoMS2Annotate function at 5 ppm.

1.4) Oligosaccharide synthesis and analysis

The automated glycan assembly (AGA) of oligosaccharides **1**, **3** and **4** followed published protocols (**Fig. S1**)[16,17]. For compound **2**, dibutyl phosphate donors have been demonstrated to allow the preparation of beta-glucans[18]. Here, thioglycoside building blocks **6** and **7** (**Fig. S12**) were found to be effective using N-iodosuccinimide (NIS) and triflic acid (TfOH) with the temperature sequence of −20 °C for 15 min, followed by 0 °C for 25 min using 6.5 equivalents of the donor (CH2Cl2/dioxane, 2:1). HPLC analysis of the crude synthesis showed no deletion sequences (**Fig. S13**). Following automated assembly, on-resin methanolysis (module f) removed the benzoate esters with progress tracked using MALDI-MS (Fig. S14). LED (370 nm) promoted photocleavage (module g) released the semi-deprotected oligosaccharide from the solid-support and the crude mixture was subject to hydrogenolysis (module h). A single preparative HPLC (HyperCarb) gave 1.1 mg of 2. The synthesis of 4-O-sulfated mannose **5** was complete using solution-phase synthesis[19].

All chemicals used were reagent grade and used as supplied unless otherwise noted. The automated syntheses were performed on a home-built synthesizer developed at the Max Planck Institute of Colloids and Interfaces. Analytical thin-layer chromatography (TLC) was performed on Merck silica gel 60 F254 plates (0.25 mm). Compounds were visualized by UV irradiation or dipping the plate in a 5% H_2SO_4 ethanol solution. Flash column chromatography was carried out on an automated Grace flash chromatography system. Analysis and purification by normal and reverse phase HPLC were performed by using an Agilent 1200 series. ^{1}H, ^{13}C and HSQC NMR spectra were recorded on a Varian 400MR (400 MHz), Varian 600MR (600 MHz), or Bruker Biospin AVANCE700 (700 MHz) spectrometer. Signals are reported in terms of chemical shift [δ in parts per million (ppm)] relative to D_2O using the solvent as the internal standard in ^{1}H NMR (D2O: 4.79 ppm ^{1}H). NMR data is presented as follows: Chemical shift, multiplicity (s = singlet, d = doublet, t = triplet, dd = doublet of doublet, m = multiplet and/or multiple resonances), coupling constant in Hertz (Hz), integration. All NMR signals were assigned on the basis of ^{1}H NMR, ^{13}C NMR, COSY, TOCSY and HSQC experiments. [‡]Carbon assignments were extracted from HSQC experiment. High resolution mass spectra were obtained using a Xevo G2-XS Q-TOF mass spectrometer (Waters). Flow rate was 0.1 mL/min, the solvent for negative ion mode was acetonitrile. For positive ion mode, the solvent was acetonitrile with 0.1% formic acid. The source settings were, capillary voltage 2.5 (kV), sampling cone 40, and source offset to 80. The source temperature was 100 °C and desolvation 250 °C. Gas flow, cone gas was set to 50 L/h and desolvation gas 600 L/h. A MALDI-TOF autoflexTM (Bruker) was used to monitor the progress of

solid-phase synthesis following microcleavage, the matrix was 2,5-dihydroxy-benzoic acid (DHB). Thioglycoside building blocks **6** and **7** were purchased from GlycoUniverse.

1.4.1) Automated glycan assembly

Solvents were taken from an anhydrous solvent system (JC Meyer-solvent systems) to prepare activator, acid wash (TMSOTf), and capping solutions. The building blocks were co-evaporated once with toluene and dried under a high vacuum before use. All solutions were freshly prepared and kept under argon during the automation run. Final yields were calculated based on the resin loading. Resin loading was determined by performing a double glycosylation followed by DBU-promoted Fmoc-cleavage and determination of dibenzofulvene formation by measuring its UV absorbance.

1.4.1.1) Preparation of reagent solutions

Building block solution: Building block (0.09 mmol) was dissolved in CH_2Cl_2 (1 mL).
NIS/TfOH activator solution: Recrystallized NIS (1.56 g) was dissolved in 40 mL of a 2:1 v/v mixture of anhydrous CH_2Cl_2 and anhydrous dioxane. Then, triflic acid (55µL, 0.6 mmol) was added. The solution was kept on an ice bath for the duration of the automation run.
Fmoc deprotection solution: Either a solution of 20% piperidine in DMF (v/v) (module E1) or a solution of 20% triethylamine in DMF (v/v) was prepared (module E2).
Acid wash solution: TMSOTf (0.45 mL, 2.49 mmol) was dissolved in CH_2Cl_2 (40 mL).
Capping solution: A 50 mL solution of 10% acetic anhydride and 2% methanesulfonic acid in CH_2Cl_2 (v/v) was prepared.
Lev deprotection solution: A solution of hydrazine acetate (725 mg) in the mixture of pyridine (40 mL), acetic acid (10 mL) and water (2.5 mL) was prepared.

1.4.1.2) Modules for Automated Solid-Phase Synthesis

Resin preparation for synthesis:

1. Conjugation-ready linker (Linker 1) with a loading of 0.4 mmol/g.

40mg of resin was placed in the reaction vessel and swollen in CH_2Cl_2 for 20 min at room temperature prior to synthesis. During this time, all reagent lines needed for the synthesis were washed and primed. Before the first glycosylation, the resin was washed with the DMF, THF, and CH_2Cl_2 (three times each with 2 mL for 25 s).

TMSOTf acidic wash solution (Module a): The resin was swollen in CH_2Cl_2 (2 mL) and the temperature of the reaction vessel was adjusted to -20 °C. Upon reaching the low temperature, TMSOTf solution (1 mL, 0.06 mmol) was added dropwise to the reaction vessel. After bubbling for 3 min, the acidic solution was drained and the resin was washed with CH_2Cl_2 (2 mL) for 25 s.

Table S19 | TMSOTf acidic wash (Module a) summary.

Action	Cycles	Solution	Amount	T (°C)	Incubation time
Cooling	-	-	-	-20	-
Deliver	1	CH_2Cl_2	2 mL	-20	-
Deliver	1	TMSOTf solution	1 mL	-20	3 min
Wash	1	CH_2Cl_2	1 mL	-20	25 s

Thioglycoside glycosylation: The building block solution (0.1 mmol of BB in 1 mL of CH_2Cl_2 per glycosylation) was delivered to the reaction vessel. After the set temperature was reached, the reaction was started by dropwise addition of the activator solution (1.0 mL, excess). After completion of the reaction, the solution is drained and the resin was washed with CH_2Cl_2, CH_2Cl_2/dioxane (1:2, v/v, 2 mL for 20 s), and CH_2Cl_2 (twice, each with 2 mL for 25 s). The temperature of the reaction vessel is increased to 25°C for the next module.

Table S20 | Thioglycoside glycosylation (Module b) summary.

Action	Cycles	Solution	Amount	T (°C)	Incubation time
Cooling	-	-	-	-20	-
Deliver	1	BB solution	1 mL	-20	-
Deliver	1	activator solution	1 mL	-20	-
Reaction time	1			-20	10 min
				to 0	25 min
Wash	1	CH_2Cl_2	2 mL	0	25 sec
Wash	1	CH_2Cl_2:Dioxane	2 mL	0	20 sec
Heating	-	-	-	25	-
Wash	1	CH_2Cl_2	2 mL	>0	25 sec

Capping (Module c): The resin was washed twice with DMF (2 mL, 25 s) and the temperature of the reaction vessel was adjusted to 25 °C. Pyridine solution (2 mL, 10% in DMF) was delivered into the reaction vessel. After 1 min, the reaction solution was drained and the resin was washed with CH_2Cl_2 (three times with 3 mL for 25 s). Capping solution (4 mL) was delivered into the reaction vessel. After 20 min, the reaction solution was drained and the resin was washed with CH_2Cl_2 (three times with 3 mL for 25 s).

Table S21 | Capping (Module c) summary.

Action	Cycles	Solution	Amount	T (°C)	Incubation time
Heating	-	-	-	25	-
Wash	2	DMF	2 mL	25	25 s
Deliver	1	10% Py./DMF	2 mL	25	1 min
Wash	3	CH_2Cl_2	2 mL	25	25 s
Deliver	1	Capping solution	4 mL	25	20 min
Wash	3	CH_2Cl_2	2 mL	-20	25 s

Fmoc deprotection with NEt_3 (Module d): The resin was washed with DMF (three times with 2 mL for 25 s) and the temperature of the reaction vessel was adjusted to 25 °C. Fmoc deprotection solution was delivered to the reaction vessel and kept under Ar bubbling (three times with 2 mL). After 5 min, the reaction solution was drained and the resin was washed with DMF (three times with 2 mL for 25 s) and CH_2Cl_2 (five times each with 2 mL for 25 s). The temperature of the reaction vessel was decreased to -20 °C for the next module.

Table S22 | Fmoc deprotection with NEt_3 (Module d) summary.

Action	Cycles	Solution	Amount	T (°C)	Incubation time
Wash	3	DMF	2 mL	25	25 s
Deliver	3	Fmoc depr.	2 mL	25	5 min
Wash	3	DMF	2 mL	25	25 s
Wash	5	CH_2Cl_2	2 mL	25	25 s
Cooling	1	-	-	-20	-

Levulinoyl ester deprotection (Module e): The resin was washed with CH_2Cl_2 (three times with 2 mL for 25 s) and the temperature of the reaction vessel was adjusted to 25 °C. 2 mL of Levulinoyl ester deprotection solution was delivered to the reaction vessel and kept under Ar bubbling. After 30 min, the reaction solution was drained and the resin was washed with DMF, THF and CH_2Cl_2 (six times each with 2 mL for 25 s).

Table S23 | Levulinoyl ester deprotection (Module e) summary.

Action	Cycles	Solution	Amount	T (°C)	Incubation time
Heating	-	-	-	25	-
Wash	3	CH_2Cl_2	2 mL	25	25 s
Deliver	1	Lev solution	2 mL	25	30 min
Wash	6	CH_2Cl_2	2 mL	25	25 s
Deliver	1	Lev solution	2 mL	25	30 min
Wash	9	CH_2Cl_2	2 mL	25	25 s
Deliver	1	Lev solution	2 mL	25	30 min
Wash	3	CH_2Cl_2	2 mL	-20	25 s
Wash	6	DMF	2mL	<25	25 s
Wash	6	THF	2mL	<25	25 s
Wash	6	CH_2Cl_2	2mL	<25	25 s

1.4.2) Solid-phase synthesis

Methanolysis (Module f): The resin was suspended in anhydrous THF (4.8 mL). Then, 0.2 mL of a solution of NaOMe in MeOH (0.5 M) was added and the resin was shaken at room temperature for 16 h. The resin was then washed successively with THF, CH_2Cl_2, methanol, and CH_2Cl_2.

Photocleavage from the solid support (Module g): Glycans were cleaved from the solid support using a batch-flow photoreactor. The resin-bound glycan (~40 mg) was suspended in DMF (4 mL) under the irradiation of an LED lamp (370nm), with stirring for 24 hours. The solution was separated from the resin using a fritted syringe and concentrated under vacuum.

1.4.3) Solution-phase synthesis

Hydrogenolysis (Module h): The crude compound was dissolved in 4 mL of THF: *t*BuOH: H_2O (60:10:30). 5% Pd-C (200 mg) was added and the reaction was stirred under H_2 atmosphere for 12 h. The reaction was filtered through a pad of celite and washed with *t*BuOH and H_2O. The filtrates were concentrated *in vacuum*, and dissolved in 3.0 mL of water for RP-HPLC purification.

1.4.4) HPLC analysis and purification

Analytical traces of crude and pure compounds were collected using an analytic RP-HPLC Agilent 1200 Series (**Methods 1** and **3**). Purification of the crudes was conducted using a preparative RP-HPLC Agilent 1200 Series (**Method 2**).

Method 1, analytic RP-HPLC (non-sulfated oligosaccharide): (Hypercarb column, 150 x 4.6 mm, 3 μm) flow rate of 0.7 mL/min with ACN/H_2O (0.1% formic acid) as eluents [isocratic 100 % H_2O (0.1% formic acid) (5 min), linear gradient to 100% ACN (30 min)].

Method 2, preparatory RP-HPLC (non-sulfated oligosaccharide): (Hypercarb column, 150 x 10 mm, 5 μm), flow rate of 3.5 mL /min with H_2O (0.1% formic acid) as eluents [isocratic 100 % H_2O (0.1% formic acid) (5 min), linear gradient to 100% ACN (30 min)].

Method 3 analytic RP-HPLC (sulfated oligosaccharide): (Hypercarb column, 150 x 4.6 mm, 3 μm) flow rate of 0.7 mL/min with ACN/H_2O (0.1mM $(NH_4)_2CO_3$) as eluents [isocratic 100 % H_2O (0.1mM $(NH_4)_2CO_3$) (5 min), linear gradient to 100% ACN (30 min)].

Following purification, all products were lyophilized on a Christ Alpha 2-4 LD plus freeze dryer before characterisation.

1.4.5) Compound characterisation

1.4.5.1) Amino pentyl β-D-glucopyranosyl-(1→3)-β-D-glucopyranosyl-(1→3)-[β-D-glucopyranosyl-(1→6)]-β-D-glucopyranosyl-(1→3)-β-D-glucopyranoside (2)

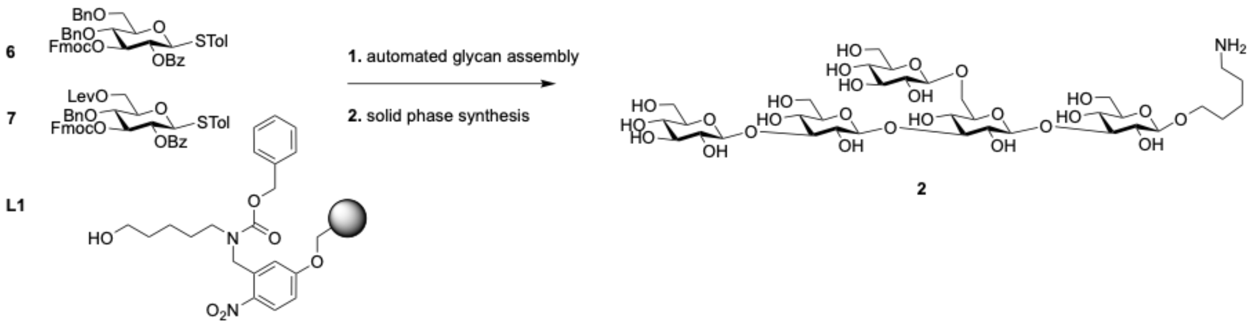

Table S24 | Compound synthesis summary.

		Modules	Notes
**1. AGA**			L1
	**6**	a, b, c, d	x1
	**7**	a, b, c, d	x2
	**6**	a, b, c, e, d	x2
	**6**	a, b, c, d	x2
	**Step**	**Module**	**Notes**
**2. Solid-phase synthesis**	Methanolysis	f	
	photocleavage	g	
	**Step**	**Module**	**Notes**
**3. Solution-phase**	Hydrogenolysis	h	
	Purification	Method 2	

The desired fractions were then collected and lyophilized to yield 1.1 mg (11%). **^{1}H NMR** (700 MHz, D_2O) δ 4.64 – 4.57 (m, 2H), 4.40 (d, *J* = 7.9 Hz, 1H), 4.36 (d, *J* = 8.1 Hz, 1H), 4.09 (d, *J* = 11.5 Hz, 1H), 3.84 – 3.73 (m, 7H), 3.69 – 3.52 (m, 9H), 3.50 – 3.39 (m, 3H), 3.39 – 3.30 (m, 8H), 3.30 – 3.20 (m, 4H), 3.17 (t, *J* = 8.7 Hz, 1H), 2.87 (t, *J* = 7.5 Hz, 2H), 1.60 – 1.51 (m, 4H), 1.38 – 1.28 (m, 2H). **‡^{13}C NMR** (176 MHz, D_2O) δ 102.7, 102.7, 102.4, 101.8, 85.0, 84.0, 83.7, 75.6, 75.5, 74.4, 73.4, 73.1, 73.1, 72.6, 70.0, 69.5, 68.6, 68.1, 67.9, 60.5, 39.2, 28.1, 26.3, 22.1. **HRMS** QTOF-MS: calcd. $C_{35}H_{64}NO_{26}$ for $[M+H]^+$ 914.3717, found 914.3712.

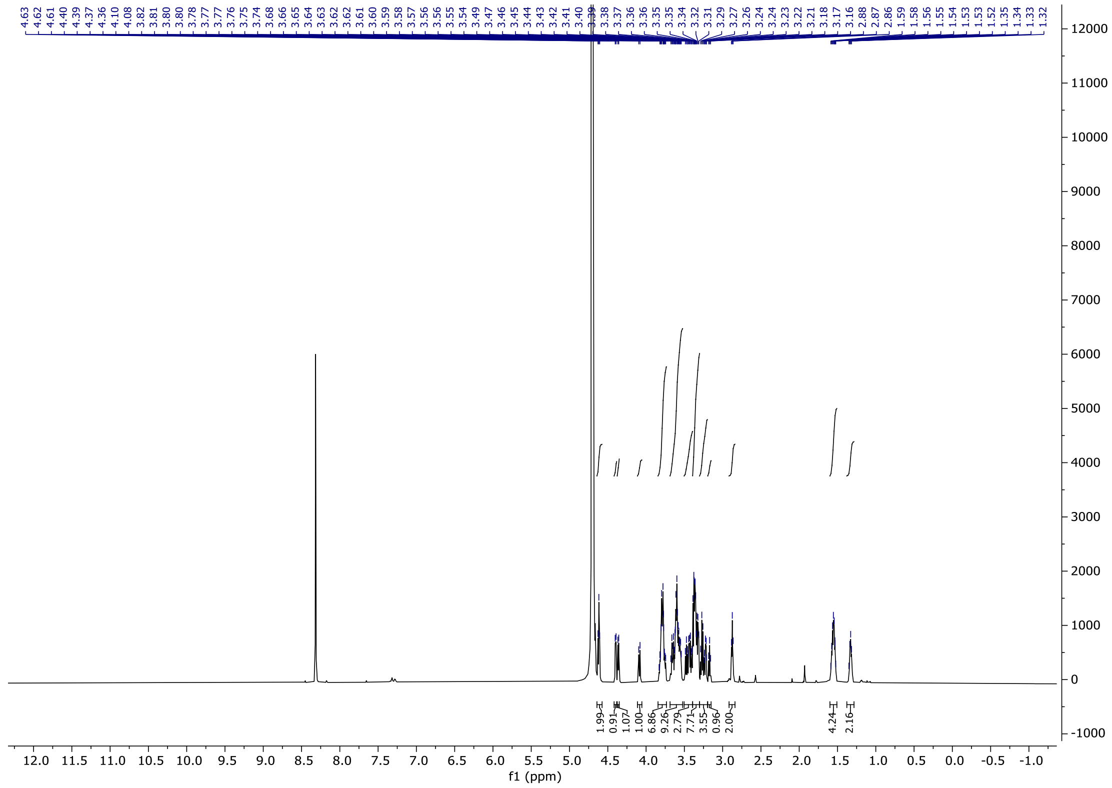

^{1}H NMR

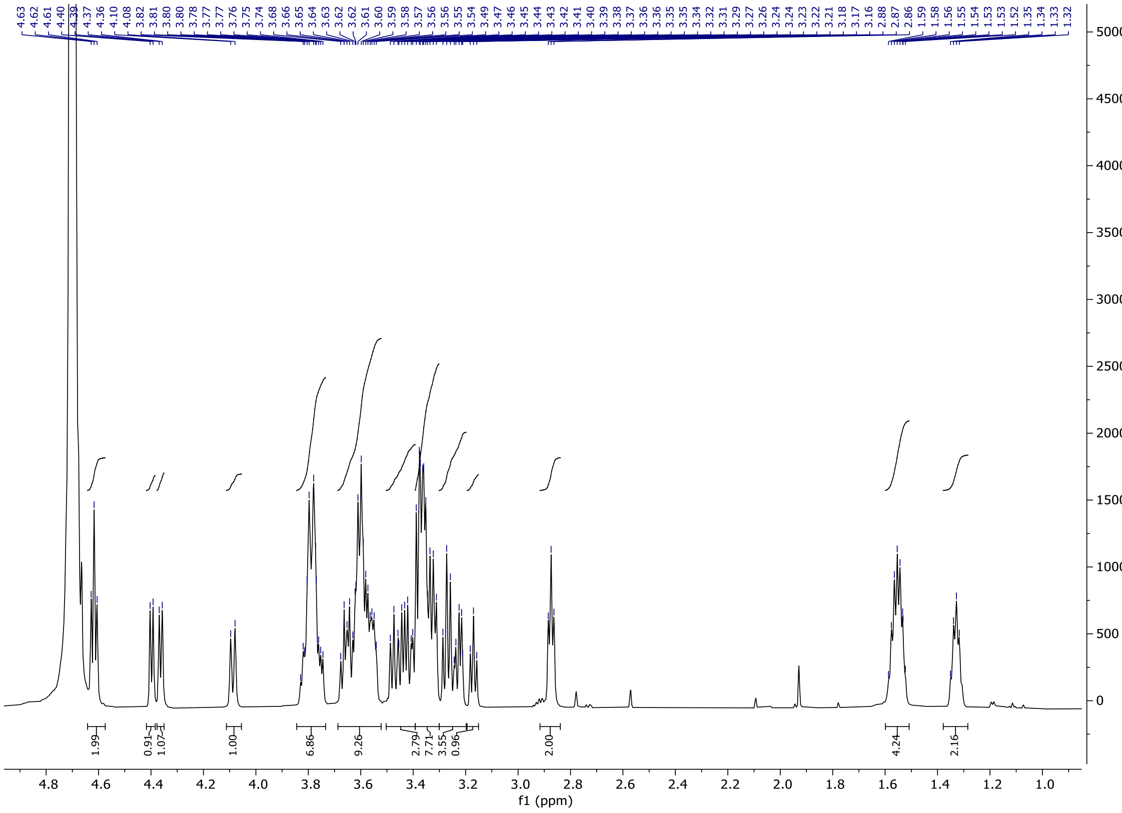

Zoomed ^{1}H NMR

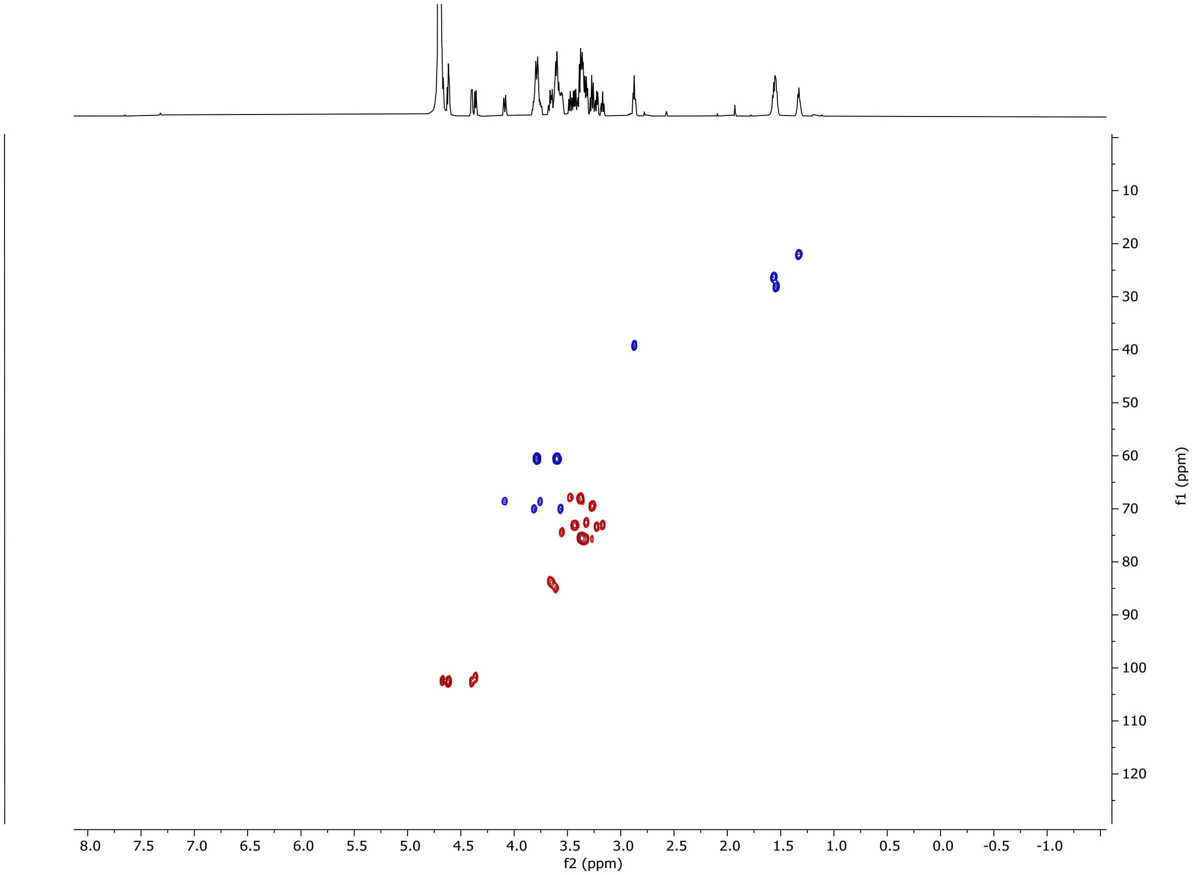

^{1}H-^{13}C HSQC

1.4.6) Data analysis

mzML files containing QTOF data were loaded into R with MSnbase[15]. Files were manually filtered by retention time to a 15 second window according to the total ion chromatogram (TIC) peak (**Fig. S2**). Several pre-processing steps were then performed with MSnbase functions. First, spectra in each filtered file were averaged (mzd = 0.001, intensity weighted, mean *m/z* and intensity). Intensities within each averaged spectrum were then normalised with respect to the maximum ion intensity, and then spectra were filtered at a normalised intensity threshold of 80%. Remaining peaks in spectra were annotated at 15 ppm by GlycoAnnotateR: DP 1-6; positive and negative polarity; amino-pentyl linker, deoxy and sulfate modifications; double sulfation possible; maximum of 2 modifications per monomer on average; H and Na adducts (singly charged only). Annotations were filtered to ensure only positive adducts were annotated in positive mode data, and only negative adducts were annotated in negative mode data.

HPLC-ELS-MS .D files were parsed with the python package ‘rainbow’ (https://github.com/evanyeyeye/rainbow) to extract ELS and MS data. Parsed data were then loaded into R. Ion chromatograms were plotted for $[M-H]^-$, $[M+H]^+$ and $[M+Na]^+$ adducts based on the QTOF data annotations (±0.1 Da), along with ELS traces.

1.5) Mouse lung MALDI-FTICR data analysis

glycoPredict from GlycoAnnotateR was used to create a custom N-glycan database using the following non-default parameters: DP 1-22; positive polarity; scan range 1-5000; sialic acid, deoxy, N-acetyl, O-acetyl, sulfate, and O-methyl modifications; MALDI-type ions; N-glycan linkage type; and upper limits for O-methyl and O-acetyl of 2. The database contained 488,647 singly charged ions from 117,966 unique compositions.

For a first check that all annotations could be reproduced, the 88 NGlycDB annotations assigned on METASPACE at a false discovery rate (FDR) of 5% were downloaded in csv format, and re-annotated at 3 ppm by GlycoAnnotateR using the custom database.

The imzML file was then downloaded from METASPACE and imported into R with Cardinal (v3.2.1)[20] at 3.03 ppm (resolution is 330,000 at 400 *m/z*[21]) to test GlycoAnnotateR in an R-based pipeline. All *m/z* values in the Cardinal object were annotated against the custom database at 3 ppm, and only annotated values were retained. This provided an initial filtering step to reduce the large size of the high mass resolution FTICR data. The data were then peak picked (MAD, SNR = 10), aligned (3 ppm), and frequency filtered (minimum frequency 0.0005) with Cardinal. Peaks were binned using the processed peak areas as references with a tolerance of 3 ppm. A feature table was extracted from the processed data, and the features were annotated with the custom database at 3 ppm. If two features received the same annotation, only the feature with the lowest mass deviation from the theoretical *m/z* value was assigned that annotation.

Processed, annotated and deisotoped data were segmented into 5 classes using the adaptive method from CARDINAL (radius 2, sparsity thresholding 20, maximum 10 clusters). The top features (ranked by t-statistic) in each class were extracted with the ‘topFeatures’ function.

1.6) *Macrocystis pyrifera* fucoidan digests in detail

Wv323 GH107 enzyme was recombinantly expressed and purified according to Crawford et al. (2024)[16]. *Macrocystis pyrifera* fucoidan (Carbosynth) was digested with 1 µM GH107_Wv323 at 3 mg m^{L-1} substrate overnight at 25°C. The buffer consisted of 25 mM bis-Tris pH 6.5, 250 mM NaCl and 1% (w/v) sea salts (Sigma-Aldrich). Digests were heat inactivated at 99°C for 10 min. The negative control contained heat-inactivated enzyme. 50 µL of the digests was mixed with ethanol for a final ethanol concentration of 95% (v/v) and desalted with silica columns (Schultz-Johansen, M. et al., *in prep*). The flow-through fractions from 95% (v/v) ethanol, 85% (v/v) ethanol and MilliQ-water were collected, and the ethanol fractions were dried by vacuum centrifugation (Eppendorf Concentrator Plus, 60°C, 4 h, V-AL mode) then reconstituted in the same volume MilliQ-water. 5 µL of each fraction was loaded on a C-PAGE gel[22] (35% polyacrylamide) with 5 µL Phenol Red loading dye. The gel was run for 30 min at 100V and 120 min at 200 V and then stained in 0.005% Stains-All solution in a 70/30 (v/v) mixture of ethanol/MilliQ-water overnight[23]. De-staining was performed in the light for ~1 hour with distilled water, and the de-stained gel was imaged with a scanner.

The 85% ethanol fractions were analysed with LC-MS/MS, using the same set-up as the commercial standards. Digests were diluted 5x with acetonitrile (LC-MS grade) for a final acetonitrile concentration of 80% (v/v). 2.5 µL were injected onto a Accucore-150-amide HILIC column (Thermo Scientific, USA) held at 60°C. Buffer A consisted of 30 mM ammonium formate, pH 5 (adjusted with formic acid) and buffer B of LC-MS grade acetonitrile with 0.1% (v/v) formic acid. The chromatography consisted of 6 steps with a flow rate of 0.4 mL min^{-1}: (1) hold for 1 min at 80% B; (2) ramp down to 60% B over 20 min; (3) ramp down from 60% to 45% B over 24 min; (4) hold for 5 min at 45% B; (5) ramp up to 80% B over 3 min; (6) equilibrate at 80% B for 14 min. Ionisation settings were: sheath gas 50, aux gas 13, sweep gas 3, spray voltage 2.5 kV, capillary temperature 263°C, S-lens RF level 55, aux gas temperature 425°C. Samples were first run in full MS mode: negative polarity, 200 - 1500 m/z, resolution 70,000, AGC target 3e6, maximum IT 100 ms. Preliminary analysis of the data using GlycoAnnotateR to calculate the *m/z* values of potential sulfated deoxy-hexose oligosaccharide ions generated a list of target ions for MS/MS. Samples were run again but with a tSIM-ddMS2 method, with a timed inclusion list (**Table S4**). SIM settings: resolution 35,000, AGC target 5e4, maximum IT 150 ms, loop count 3, isolation window 0.4 *m/z*. dd-MS2 settings: resolution 35,000, AGC target 2e5, maximum IT 150 ms, loop count 3, isolation window 0.4 *m/z*, NCE stepped 10, 20, 25. dd settings: minimum AGC target

1e2, apex trigger, charge exclusion, peptide match and dynamic exclusion all off, isotope exclusion on.

LC-MS were pre-processed in R using XCMS[12] v4.0.2. Chromatographic peaks were picked with the CentWave algorithm[14] (ppm = 10, peakwidth from 3-60, integrate = 2, snthresh = 1) and then merged (minProp = 0.5, expandRt = 20). The peak table was annotated with the glycoAnnotate function of GlycoAnnotateR with the following parameters: dp 1-10, sulfate and deoxy modifications, double_sulfate true, nmod_max 3, adducts H, filtered for charge of 1 or 2, error 3.5 ppm.

MS/MS spectra associated with the annotated peaks were extracted from the tSIM-ddMS2 data using the glycoMS2Extract function of GlycoAnnotateR (processing_level = 'peaks'). MS/MS spectra were processed using functions from the Spectra package[24] v1.12.0. Fragment ion peaks were first combined within spectra (tolerance = 0.005, ppm = 3) and then averaged across spectra, using the peak index as the grouping variable (tolerance = 0.005, ppm = 3, peaks = intersect, minProp = 0.2, intensityFun = max). After converting the combined and averaged spectra to data.frame format, precursors were annotated using the same glycoPredictParam settings as annotation of the MS1 peaks, except at 5 ppm. Fragment ions were then annotated using the glycoMS2Annotate function (error = 3 ppm, nmod_max = 3, double_sulfate = TRUE, dehydrations = TRUE).

2. Isomer calculation

We attempted to define the combinatorial space of isomers for each composition, assuming changes in linkage position and stereochemistry, monomer stereochemistry, and order. Branching and non-reducing oligosaccharides were not considered. Modifications were not considered here due to the difficulty of representing each of their conditions in combinatorial form. The number of isomers can be calculated using Equation 2.

$$I = 2^d \times 2^{H+3d} \times \frac{d!}{H!\,(d-H)!} \times (4^H \times 3^{d-H} \times (\frac{d-H}{3d} + \frac{H}{4d})) \times 2^d \quad \text{Equation 2}$$

Where I is the number of isomers, d is the degree of polymerisation, and H is the number of hexoses in the chain. This formula assumes two types of monomers: pentoses and hexoses. The first term takes into account the linkage stereochemistry, i.e., alpha or beta linkage, as well as the anomeric carbon on the reducing end. The second term takes into account the stereochemistry of the chiral carbons within each monomer, i.e., the orientation of the hydroxyl groups and sixth carbon. This term therefore includes L/D isomers. The third term accounts for the order in the sequence of the monomers, which is necessary due to the inclusion of pentoses. For example, if there is a tetrasaccharide (*d* = 4) with two hexoses and two pentoses (*H* = 2), 6 sequences are possible: Pent-Pent-Hex-Hex, Hex-Pent-Pent-Hex, Pent-Hex-Hex-Pent, Hex-Pent-Hex-Pent, Hex-Hex-Pent-Pent, or Pent-Hex-Pent-Hex. When all monomers in the chain are only hexoses or only pentoses this term is equivalent to 1. The fourth term accounts for the linkage positions, considering the frequencies of hexoses and pentoses. The last term accounts for the ring form, i.e., pyranose or furanose. Thus, for an oligomer of length two, with one pentose and one hexose, the number of isomers is 14336 without considering any modifications.

Given the same conditions, Equation 2 returns the same number as Equation A' in Laine's 1994 commentary[25] i.e., the equation for linear structures with repeating hexoses. Laine's Equation A' is represented here as Equation 3.

$$S* = E^n \times 2_a^n \times 2_r^n \times (4^{n-1}) \quad \text{Equation 3}$$

Where S* is the number of isomers, E is the number of different kinds of monomers (epimers), and n is the number of different hexoses in a string (i.e., the degree of polymerisation. E^n is the linear permutation term, 2_a^n is the term for anomeric isomers, 2_r^n is the term for ring form and 4^{n-1}

is the linkage position term. For a hexasaccharide composed of only hexoses (d = 6, H = 6), according to Equation 2 there are 70 368 744 177 664 (or 70 trillion) isomers. To be equivalent, for Equation 3 we need to consider all hexose monomers that are possible, including D and L isomers, which can be found as 2^4, or 16. With an E value of 16 and an n value of 6, Equation 3 also gives 70 368 744 177 664 isomers.

In this way, we abstracted the 1994 equations of Laine[25] so that a monomer set does not need to be pre-defined, and expanded the calculations to include pentoses. According to our calculations, the number of possible linear structures for a reducing hexasaccharide composed of pentoses and/or hexoses is approximately 5.2 x 10^{14}. Comparison of the total number of linear structures for different degrees of polymerization with hexoses only or with hexose and pentoses is shown in **Fig. 1C.**

Supplementary references

(1) Yang, J.; Xie, D.; Ma, X. Recent Advances in Chemical Synthesis of Amino Sugars. *Molecules*. MDPI June 1, 2023. https://doi.org/10.3390/molecules28124724.

(2) Bligh, M.; Nguyen, N.; Buck-Wiese, H.; Vidal-Melgosa, S.; Hehemann, J. H. Structures and Functions of Algal Glycans Shape Their Capacity to Sequester Carbon in the Ocean. *Curr Opin Chem Biol* **2022**, *71*. https://doi.org/10.1016/j.cbpa.2022.102204.

(3) McNaught, A. D. Nomenclature of Carbohydrates (IUPAC Recommendations 1996). **1996**, *68* (10), 1919–2008. https://doi.org/doi:10.1351/pac199668101919.

(4) Clayden, J.; Greeves, N.; Warren, S. *Organic Chemistry*; Oxford University Press, 2023. https://doi.org/10.1093/hesc/9780199270293.001.0001.

(5) Wiberg, K. B. The Concept of Strain in Organic Chemistry. *Angewandte Chemie International Edition in English* **1986**, *25* (4), 312–322. https://doi.org/https://doi.org/10.1002/anie.198603121.

(6) Bredt, J. Über Sterische Hinderung in Brückenringen (Bredtsche Regel) Und Über Die Meso-Trans-Stellung in Kondensierten Ringsystemen Des Hexamethylens. *Justus Liebigs Ann Chem* **1924**, *437* (1), 1–13. https://doi.org/https://doi.org/10.1002/jlac.19244370102.

(7) Mak, J. Y. W.; Pouwer, R. H.; Williams, C. M. Natural Products with Anti-Bredt and Bridgehead Double Bonds. *Angewandte Chemie International Edition* **2014**, *53* (50), 13664–13688. https://doi.org/https://doi.org/10.1002/anie.201400932.

(8) Mirabella, S.; Cardona, F.; Goti, A. From Glycals to Aminosugars: A Challenging Test for New Stereoselective Aminohydroxylation and Related Methodologies. *Org. Biomol. Chem.* **2016**, *14* (23), 5186–5204. https://doi.org/10.1039/C6OB00649C.

(9) Ali, M. M. N.; Aich, U.; Varghese, B.; Serge, P.; Imberty, A.; Duraikkannu, L. Conformational Preferences of the Aglycon Moiety in Models and Analogs of GlcNAc-Asn Linkage: Crystal Structures and Ab Initio Quantum Chemical Calculations of N-(β-d-Glycopyranosyl)Haloacetamides. *J Am Chem Soc* **2008**, *130* (26), 8317–8325. https://doi.org/10.1021/ja800335m.

(10) Hazelard, D.; Compain, P. Nucleophilic Ring-Opening of 1,6-Anhydrosugars: Recent Advances and Applications in Organic Synthesis. *European J Org Chem* **2021**, *2021* (24), 3501–3515. https://doi.org/https://doi.org/10.1002/ejoc.202100403.

(11) Mölder, F.; Jablonski, K. P.; Letcher, B.; Hall, M. B.; Tomkins-Tinch, C. H.; Sochat, V.; Forster, J.; Lee, S.; Twardziok, S. O.; Kanitz, A.; Wilm, A.; Holtgrewe, M.; Rahmann, S.; Nahnsen, S.; Köster, J. Sustainable Data Analysis with Snakemake. *F1000Res* **2021**, *10*, 33. https://doi.org/10.12688/f1000research.29032.1.

(12) Smith, C. A.; Want, E. J.; O'Maille, G.; Abagyan, R.; Siuzdak, G. XCMS: Processing Mass Spectrometry Data for Metabolite Profiling Using Nonlinear Peak Alignment, Matching, and Identification. *Anal Chem* **2006**, *78* (3), 779–787. https://doi.org/10.1021/ac051437y.

(13) Kuhl, C.; Tautenhahn, R.; Böttcher, C.; Larson, T. R.; Neumann, S. CAMERA: An Integrated Strategy for Compound Spectra Extraction and Annotation of Liquid Chromatography/Mass Spectrometry Data Sets. *Anal Chem* **2012**, *84* (1), 283–289. https://doi.org/10.1021/ac202450g.

(14) Tautenhahn, R.; Bottcher, C.; Neumann, S. Highly Sensitive Feature Detection for High Resolution LC/MS. *BMC Bioinformatics* **2008**, *9*, 1–16. https://doi.org/10.1186/1471-2105-9-504.

(15) Gatto, L.; Gibb, S.; Rainer, J. MSnbase, Efficient and Elegant R-Based Processing and Visualization of Raw Mass Spectrometry Data. *J Proteome Res* **2021**, *20* (1), 1063–1069. https://doi.org/10.1021/acs.jproteome.0c00313.

(16) Crawford, C. J.; Schultz-Johansen, M.; Luong, P.; Vidal-Melgosa, S.; Hehemann, J.-H.; Seeberger, P. H. Automated Synthesis of Algal Fucoidan Oligosaccharides. *J Am Chem Soc* **2024**. https://doi.org/10.1021/jacs.4c02348.

(17) Danglad-Flores, J.; Leichnitz, S.; Sletten, E. T.; Abragam Joseph, A.; Bienert, K.; Le Mai Hoang, K.; Seeberger, P. H. Microwave-Assisted Automated Glycan Assembly. *J Am Chem Soc* **2021**, *143* (23), 8893–8901. https://doi.org/10.1021/jacs.1c03851.

(18) Weishaupt, M. W.; Hahm, H. S.; Geissner, A.; Seeberger, P. H. Automated Glycan Assembly of Branched β-(1,3)-Glucans to Identify Antibody Epitopes. *Chemical Communications* **2017**, *53* (25), 3591–3594. https://doi.org/10.1039/c7cc00520b.

(19) Krull, J.; Crawford, C. J.; Sidhu, C.; Solanki, V.; Bligh, M.; Rößler, L.; Singh, R. K.; Huang, G.; Robb, C. S.; Teeling, H.; Seeberger, P. H.; Schweder, T.; Hehemann, J.-H. Polyelectrolyte Mannan from Diatoms Reshapes Sunlit Ocean Microbiome. *bioRxiv* **2024**. https://doi.org/10.1101/2024.07.03.601839.

(20) Bemis, K. A.; Föll, M. C.; Guo, D.; Lakkimsetty, S. S.; Vitek, O. Cardinal v.3: A Versatile Open-Source Software for Mass Spectrometry Imaging Analysis. *Nat Methods* **2023**, *20* (12), 1883–1886. https://doi.org/10.1038/s41592-023-02070-z.

(21) Veličković, D.; Bečejac, T.; Mamedov, S.; Sharma, K.; Ambalavanan, N.; Alexandrov, T.; Anderton, C. R. Rapid Automated Annotation and Analysis of N-Glycan Mass Spectrometry Imaging Data Sets Using NGlycDB in METASPACE. *Anal Chem* **2021**, *93* (40), 13421–13425. https://doi.org/10.1021/acs.analchem.1c02347.

(22) Robb, M.; Hobbs, J. K.; Boraston, A. B. Separation and Visualization of Glycans by Fluorophore-Assisted Carbohydrate Electrophoresis. In *Protein-Carbohydrate Interactions. Methods in Molecular Biology*; Abbot, D., Lammerts van Bueren, A., Eds.; Humana Press: New York, NY, 2017; Vol. 1588, pp 215–221. https://doi.org/10.1007/978-1-4939-6899-2_17.

(23) Andrade, J. P. S.; Oliveira, C. P.; Tovar, A. M. F.; Mourão, P. A. de S.; Vilanova, E. A Color-Code for Glycosaminoglycans Identification by Means of Polyacrylamide Gel Electrophoresis Stained with the Cationic Carbocyanine Dye Stains-All. *Electrophoresis* **2018**, *39* (4), 666–669. https://doi.org/10.1002/elps.201700391.

(24) Rainer, J.; Vicini, A.; Salzer, L.; Stanstrup, J.; Badia, J. M.; Neumann, S.; Stravs, M. A.; Hernandes, V. V.; Gatto, L.; Gibb, S.; Witting, M. A Modular and Expandable Ecosystem for Metabolomics Data Annotation in R. *Metabolites* **2022**, *12* (2). https://doi.org/10.3390/metabo12020173.

(25) Laine, R. A Calculation of All Possible Oligosaccharide Isomers Both Branched and Linear Yields 1.05 × 1012 Structures for a Reducing Hexasaccharide: The Isomer Barrier to Development of Single-Method Saccharide Sequencing or Synthesis Systems. *Glycobiology* **1994**, *4*, 759–767.
